# Use of Different Food Image Recognition Platforms in Dietary Assessment: Comparison Study

**DOI:** 10.2196/15602

**Published:** 2020-12-07

**Authors:** Stephanie Van Asbroeck, Christophe Matthys

**Affiliations:** 1 Department of Chronic Diseases and Metabolism Clinical and Experimental Endocrinology KU Leuven Leuven Belgium

**Keywords:** image recognition, dietary assessment, automated food recognition, accuracy

## Abstract

**Background:**

In the domain of dietary assessment, there has been an increasing amount of criticism of memory-based techniques such as food frequency questionnaires or 24 hour recalls. One alternative is logging pictures of consumed food followed by an automatic image recognition analysis that provides information on type and amount of food in the picture. However, it is currently unknown how well commercial image recognition platforms perform and whether they could indeed be used for dietary assessment.

**Objective:**

This is a comparative performance study of commercial image recognition platforms.

**Methods:**

A variety of foods and beverages were photographed in a range of standardized settings. All pictures (n=185) were uploaded to selected recognition platforms (n=7), and estimates were saved. Accuracy was determined along with totality of the estimate in the case of multiple component dishes.

**Results:**

Top 1 accuracies ranged from 63% for the application programming interface (API) of the Calorie Mama app to 9% for the Google Vision API. None of the platforms were capable of estimating the amount of food. These results demonstrate that certain platforms perform poorly while others perform decently.

**Conclusions:**

Important obstacles to the accurate estimation of food quantity need to be overcome before these commercial platforms can be used as a real alternative for traditional dietary assessment methods.

## Introduction

For many years, research has been conducted in order to elucidate the relationship between diet and health outcomes [1], and associations between diet and noncommunicable diseases like type 2 diabetes, cancer, and obesity have been observed [2-4]. These findings have recently been questioned due to criticism of the dietary assessment methods used [5-7]. In epidemiological research, diet is most often assessed via food frequency questionnaires or 24-hour dietary recalls [8-10]. These assessment methods have long been known to be sensitive to both random and systematic error because they are based on self-reporting and memory of the participant [10,11]. However, better alternatives are not readily available. To assess diet in a research context, cost and time obviously play a role [11]. Additionally, the burden for participants needs to be reduced as much as possible [11,12]. Furthermore, cognition of the participant is another factor to take into account. Here, especially, the estimation of food amounts has proven to be a difficult task for most [8,11].

Therefore, the accurate assessment of diet currently remains a problem [13]. One suggested alternative is the automated analysis of pictures of the participant’s food via image recognition [8,13,14]. The automated analysis would not require a high level of literacy or the recall of what was eaten in the past [15]. Additionally, it would reduce the systematic bias that is almost inherent to self-reporting of dietary intake toward what is socially preferred [11].

Nonetheless, automated image recognition of food along with the estimation of the amount of food shown in the image is not an easy task. Food items usually do not have a set shape or structure. Instead, they are deformable in nature. Additionally, similar foods can look very different (high intraclass variance) while different foods can look quite similar (interclass variance). Beverages are especially difficult to assess because only a limited amount of information can be acquired visually [16].

Multiple groups have tried to tackle the challenge that image recognition of food poses. Usually these groups also conduct tests on the performance of their final product. Often they report quite good results with accuracies ranging from 44% to 97.2% [8,13,16-21]. However, they often test their system in ideal conditions (eg, a well-lit room, food on a white plate nicely photographed in the frame) [13]. Another option is that they test their system on pictures from a food dataset, sometimes the same dataset that was used for training of the recognition system, so called cross-validation of machine learning [16,18,20,21]. Several food image recognition platforms have been created, but it is important to choose or create a dataset representative of food consumed in real-life situations [18]. The latter is key in the future development of mobile health apps that would assist patients in the context of dietary management of specific pathologies. A recent paper reviewed the possibilities of different food object recognition systems for use on a mobile phone. Based on their findings, they provided a categorization of mobile food recognition systems but reported that it is difficult to conduct an objective comparison between different systems as they all use different testing scenarios [22]. Therefore, a comparative study was set up to determine how well commercially available food image recognition systems currently perform in different settings and what the biggest challenges are when striving for accurate identifications.

## Methods

### Study Design

A comparative study on the accuracy of image recognition platforms for the recognition of food and beverages was conducted. Relevant application programming interfaces (APIs) and apps were searched for and all were submitted for pretesting. The tested platforms are described in Table 1.

Amazon Rekognition was not tested because this platform does not offer a demo version. Additionally, Snappy Meal was incompatible with our system and could therefore not be tested. All pictures used in this study were taken on the same device, a Galaxy Tab A6 (Samsung Electronics Co Ltd) tablet.

**Table 1:** Selected platforms identifying food images.

Platform	Version	Specifically developed for food
Google Vision API^a^	Unknown	No
IBM Watson Recognition	Unknown	No; employs a food module when estimating food in general model
Amazon Rekognition	Unknown	No
LogMeal	Unknown	Yes
FoodAI	Unknown	Yes
Clarifai	1	No; we used the included module developed specifically for food
Snappy Meal	0.0.1	Yes
Lose It	9.6.14	Yes
Bitesnap	1.5.6	Yes
Foodvisor	2.3	Yes
Calorie Mama API	Unknown	Yes

^a^API: application program interface.

### Pretesting

Pretesting was done in order to select those platforms that could recognize 3 well-known food items considered easy to recognize (a whole banana, one slice of white bread, one round cookie); a clementine was also included in the test. These food items were photographed with a tablet by the same researcher. Foods were presented on a white round plate in a well-lit room on a table free of clutter. The whole plate was always in frame. Pictures were taken in 3264×2448 pixel size. The same picture of each food item was fed into all recognition platforms in random order on 1 day. Calorie Mama API required pictures to be resized to 544×544 pixels. Hence, for Calorie Mama API, the pictures were first resized using Pixlr Express web tool. Identifications of the image recognition process were gathered.

If a recognition platform succeeded in the correct top 1 identifications of at least 1 out of 3 food items combined with at least some form of recognition of the clementine as being a citrus, orange, or tangerine, it would be selected for more thorough testing.

### Photographing Foods and Beverages

**Figure 1:**
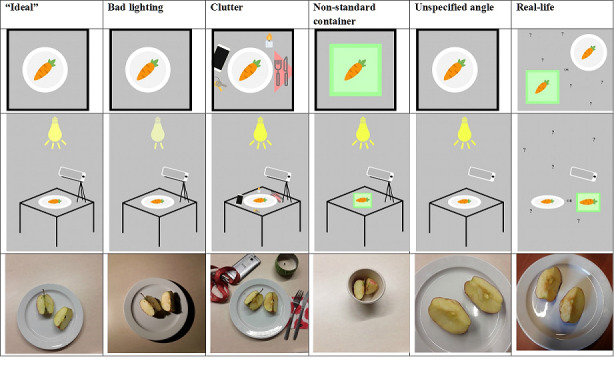
Scheme and example pictures of the photographing settings used.

For further detailed testing of the image recognition platforms, pictures of a wide range of foods and beverages were taken in different settings. The ideal setting is in line with the guidelines defined by Rhyner et al [23]. As illumination and occlusion can play a role in the quality of the picture and consequently in the possibility of recognizing a food image, different conditions have been created. Both settings (eg, light, angle, height) and circumstances (eg, other objects on a table) can decrease the quality of the food image recognition. Using a stepwise approach, we have created different conditions. The settings used are defined in Table 2, and examples are shown in Figure 1. The use of different settings allowed us to compare the performance of the image recognition platforms in specific circumstances.

The original size of all pictures was 1920×1920 pixels, and all were resized to 544×544 pixels. Selection of food items, meals, and drinks was done to achieve a set of widely varying dishes in terms of components and difficulty while also keeping in mind selecting well-known, regularly consumed foods and beverages. The selection of the foods followed a stepwise approach. First, 5 simple, plain food items were selected. These were defined as single, unprocessed food items that can be eaten on their own. Next, we selected 5 plain, processed food items. Again, these are single food items that can be eaten on their own. Additionally, we selected 7 common hot and cold drinks. Last, 12 mixed dishes were selected with widely varying key components. The selected foods, beverages, and meals reflect the Belgian dietary pattern. All selected items are listed in Multimedia Appendix 1. Each food, beverage, or meal was photographed 6 times, each time in a different setting. All pictures were taken in the same room on the same solid colored table except for the real-life setting. All pictures were taken by the same researcher unless specified otherwise. All pictures are available in Multimedia Appendix 2.

**Table 2:** Predefined settings and circumstances pictures were taken.

Setting	Circumstances			
	Lighting	Other objects on table	Container	Angle and height of tablet
Ideal	Well lit	None	Standard^a^	Standardized height (whole plate needed to be in frame) Standardized angle (15±3°) [23]
Bad lighting	Poorly lit	None	Standard	Standardized height (whole plate needed to be in frame) Standardized angle (15±3°) [23]
Clutter	Well lit	Fork, knife, napkin, keys, candle, and smartphone	Standard	Standardized height (whole plate needed to be in frame) Standardized angle (15±3°) [23]
Nonstandard container	Well lit	None	Nonstandard^b^	Standardized height (whole plate needed to be in frame) Standardized angle (15±3°) [23]
Unspecified angle	Well lit	None	Standard	Undefined: coworkers were asked to take the picture without guidance on how to hold the tablet; no specification about height and angle
Real-life	Undefined	Undefined	About one-half on standard container and one-half on nonstandard container	Undefined: coworkers were asked to take the picture without guidance on how to hold the tablet; no specification about height and angle

^a^Standard containers: white, round plate and translucent glass or white mug.

^a^Nonstandard containers: colored glasses, plates with designs and different shapes, colored mugs, bowls made out of different materials and colors.

### Automatic Image Recognition

All images were entered into the selected image recognition platforms (Table 1). This procedure was done in random order within 2 consecutive days during mid-December 2018 to avoid any automatic learning curve of any of the platforms. The top 1 and top 5 best identifications were saved.

### Calculations

#### Reference Food Items for Identification

For each image, it was assessed whether the top 1 and top 5 identifications were correct; this was deemed correct when the key food item was identified correctly. More specifically, meats and poultry had to be identified to the level of species or specific dish (eg, stew, curry). Fruits and vegetables had to be identified at least to the level of a core term as defined in FoodEx2, the food classification system used by the European Food Safety Authority to standardize nutrient and exposure assessment. Carbohydrate sources had to be identified to the level of the key ingredient (eg, pasta, rice, or potatoes). Pancakes had to be identified as such. Cornflakes had to be identified as cereal, cornflakes, granola, or muesli. A Snickers bar could be identified as being Snickers, a candy bar, chocolate bar, or chocolate (fudge). Chocolate spread could be identified as such, as Nutella, or as chocolate. Milk had to be identified as such. Detail on fat content was not necessary or expected, but soy milk had to be identified specifically as soy milk to be deemed correct. Vanilla pudding had to be identified as custard, pudding, or a term describing a highly similar dish such as crème caramel or crème Catalan. Different sauces that were present in the picture needed to be identified at least in general as being a sauce. Water, coffee, tea, and beer needed to be identified as such, while for Coca Cola an identification of soda, soft drink, or Coke was deemed correct. For mixed dishes, an identification was deemed correct if at least one of the key components was identified correctly. Totality of the identification was also assessed for mixed dishes (see below). The top 1 and top 5 accuracy were calculated as described below.

#### Accuracy

Accuracy was calculated as the number of images correctly classified (top 1 and top 5) divided by the total number of images for each image recognition platform, each setting, and each of the predefined food categories (simple plain, simple processed, drinks, mixed dishes). To measure the accuracy of the different platforms, we used the food terms proposed by the platforms. These food terms are compared with the descriptions of the selected food items.

In addition, the image recognition tool should recognize all the individual components or use a term that describes these multiple components (eg, Quiche Lorraine) when provided with an image of a mixed dish. Hence, for mixed dishes, the proportion of components that were correctly classified within the top 5 identifications was also calculated.

#### Totality

Totality was calculated as the number of dish components correctly classified divided by the total number of dish components. A list of all mixed dishes along with their respective key components can be found in Multimedia Appendix 3. None of the mixed dishes had more than 5 key components.

### Statistical Analysis

Accuracy was compared among the different image recognition platforms as well as among the different settings and dish categories using chi-square tests. All tests were considered significant when *P*<.05. Tests were performed using SPSS Statistics 25 (IBM Corporation).

## Results

### Pretesting

Based on the platforms’ performances during pretesting, Bitesnap, Foodvisor, LogMeal, Clarifai, IBM Watson Recognition, Google Vision API, and Calorie Mama API were selected for further testing. Snappy Meal did not return any results during pretesting and was thus deemed to malfunction.

### Accuracy

In total, 185 pictures were uploaded on the different platforms. This number consists of 30 images of simple, plain foods (5 foods × 6 settings), 36 images of simple, processed foods (6 foods × 6 settings), 47 images of beverages (8 drinks × 6 settings plus 1 for Coca Cola in a can, which cannot be photographed in a nonstandard container), and 72 images of mixed dishes (12 dishes × 6 settings). Recognition accuracy based on the top 1 and top 5 best identifications was compared among the different recognition platforms and varied widely. Both for top 1 and top 5 identifications, Calorie Mama API was most accurate with 62.9% (117/185) and 87.6% (163/185), respectively. Bitesnap also performed well with a top 1 and top 5 accuracy of 48.9% (91/185) and 71.0% (132/185), respectively. Foodvisor performed at a similar level with a top 1 and top 5 accuracy of 46.2% (86/185) and 71.5% (133/185), respectively. The results of all platforms among all settings are shown in Table 3.

**Table 3:** Accuracy of food recognition among different platforms and different settings (n=31/setting).

Platform		Ideal, n (%)	Bad lighting, n (%)	Nonstandard container, n (%)	Clutter, n (%)	Unspecified angle, n (%)	Real-life, n (%)	Total, n (%)
**Top 1 accuracy**								
	LogMeal	8 (25.8)	6 (19.4)	5 (16.1)	6 (19.4)	12 (38.7)	8 (25.8)	45 (24.2)
	Clarifai	12 (38.7)	9 (29.0)	12 (38.7)	8 (25.8)	17 (54.8)	13 (41.9)	71 (38.2)
	Google Vision API^a^	2 (6.5)	3 (9.7)	1 (3.2)	0 (0)	7 (22.6)	4 (12.9)	17 (9.1)
	IBM Watson Recognition	10 (32.3)	5 (16.1)	5 (16.1)	7 (226)	8 (25.8)	12 (38.7)	47 (25.3)
	Calorie Mama API	20 (64.5)	16 (51.6)	18 (58.1)	19 (61.3)	24 (77.4)	20 (64.5)	117 (62.9)
	Bitesnap	16 (51.6)	11 (35.5)	13 (41.9)	15 (45.2)	21 (67.7)	16 (51.6)	91 (48.9)
	Foodvisor	10 (32.3)	13 (41.9)	15 (48.4)	16 (51.6)	19 (61.3)	13 (41.9)	86 (46.2)
	Total	78 (35.9)	63 (29.0)	69 (32.9)	70 (32.3)	108 (49.8)	86 (39.6)	—^b^
**Top 5 accuracy**								
	LogMeal	14 (45.2)	13 (41.9)	10 (32.3)	14 (45.2)	17 (54.8)	14 (45.2)	82 (44.1)
	Clarifai	21 (67.7)	19 (61.3)	18 (58.1)	18 (58.1)	23 (74.2)	20 (64.5)	119 (64.0)
	Google Vision API	7 (22.6)	7 (22.6)	2 (6.5)	3 (9.7)	16 (51.6)	10 (32.3)	45 (24.2)
	IBM Watson Recognition	15 (48.4)	10 (32.3)	10 (32.3)	10 (32.3)	20 (64.5)	16 (51.6)	81 (43.5)
	Calorie Mama API	27 (87.1)	28 (90.3)	25 (80.6)	26 (83.9)	29 (93.5)	28 (90.3)	163 (87.6)
	Bitesnap	23 (74.2)	20 (64.5)	20 (64.5)	21 (67.7)	28 (90.3)	20 (64.5)	132 (71.0)
	Foodvisor	19 (61.3)	19 (61.3)	24 (77.4)	21 (67.7)	26 (83.9)	24 (77.4)	133 (71.5)
	Total	126 (58.1)	116 (53.5)	109 (51.9)	113 (52.1)	159 (73.3)	132 (60.8)	—

^a^API: application programming interface.

^b^Not applicable.

When comparing the different settings, the unspecified angle setting resulted in a better recognition accuracy compared with all other settings. A comparison of the recognition accuracy among the different settings is shown in Table 3.

The accuracy was also compared among different food and beverage categories (eg, simple plain foods, simple processed foods, beverages, and mixed dishes, Table 4). Simple, plain foods, simple, processed foods, and beverages were all recognized less accurately than mixed dish components at top 1 level (*P*<.001, *P*=.02, and *P*=.004, respectively). At the top 5 level, mixed dish components were recognized significantly more accurately than drinks (*P*=.01). Additionally, simple, plain foods were recognized significantly less accurately than simple, processed foods, and drinks (*P*=.02 and *P*=.04, respectively) at the top 1 level.

**Table 4:** Accuracy of image recognition among different food categories.

Platform	Food category n (%)								
	Simple plain		Simple processed		Beverages		Mixed dishes		
	Top 1	Top 5	Top 1	Top 5	Top 1	Top 5		Top 1	Top 5
Bitesnap	11 (36.6)	25 (83.3)	14 (46.6)	18 (60.0)	22 (53.6)	33 (80.4)		44 (61.1)	56 (77.7)
Calorie Mama API^a^	22 (73.3)	29 (96.6)	23 (76.6)	31 (100)	19 (46.3)	29 (70.7)		50 (69.4)	70 (97.2)
Clarifai	4 (13.3)	13 (43.3)	14 (46.6)	27 (90.0)	17 (41.4)	30 (73.1)		36 (50.0)	49 (68.1)
Foodvisor	3 (10.0)	15 (50.0)	16 (53.3)	22 (73.3)	23 (56.1)	32 (78.1)		44 (61.1)	64 (88.8)
Google Vision API	0 (0)	0 (0)	12 (40.0)	17 (56.6)	1 (2.4)	9 (21.9)		4 (5.5)	19 (26.4)
IBM Watson Recognition	5 (16.6)	16 (53.3)	9 (30.0)	16 (53.3)	4 (9.7)	12 (29.2)		29 (40.2)	37 (51.3)
LogMeal	9 (30.0)	23 (76.6)	3 (10.0)	13 (43.3)	13 (31.7)	15 (36.5)		20 (27.7)	31 (43.1)
All	54 (25.7)	121 (57.6)	91 (43.3)	148 (70.5)	99 (34.5)	160 (55.7)		227 (45.1)	326 (64.6)

^a^API: application programming interface.

### Totality

**Figure 2:**
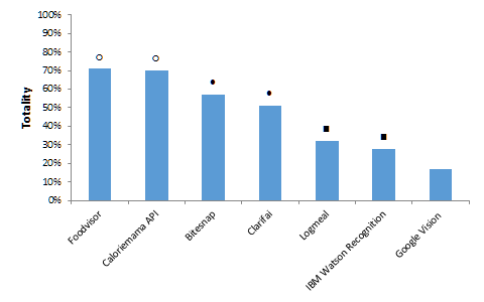
Totality of estimates on mixed dishes among different recognition platforms. Non-significant differences are denoted with a symbol placed with both platforms. All other platforms estimated mixed dish content to a significantly different level of totality (all *P* values ≤.001).

Totality of identifications for each platform are shown in Figure 2. Foodvisor and Calorie Mama API succeeded in the estimation of most dish components in their top 5 identifications with a totality of 70.8% (119/168) and 69.6% (117/168), respectively. Bitesnap and Clarifai identified mixed dish components with a totality of 56.8% (96/168) and 50.6% (85/168), respectively.

## Discussion

### Principal Findings

A series of currently available image recognition platforms were tested for their performance on the recognition of foods, beverages, and mixed dishes conducted in a number of different standardized settings and in a real-life setting. Performance in terms of accuracy was found to vary widely between platforms ranging from 9% to 63% for top 1 accuracy. Top 5 accuracy ranged from 24% to 88% over all tested platforms. For mixed dishes, a high variance in the number of recognized dish components among the platforms was found. Foodvisor and Calorie Mama API succeeded in recognition of most of the components of the mixed dish (71% and 70%, respectively) while Google Vision API only succeeded in the recognition of 17% of the key components of the mixed dishes. None of the platforms was able to identify the respective portion size of the different foods and beverages.

### Comparison of Platforms

The different tested platforms showed large differences in their performance. Calorie Mama API was the most accurate platform with a top 1 accuracy of 63% and a top 5 accuracy of 88%. Bitesnap and Foodvisor came in second place with a top 1 accuracy of 49% and 46%, respectively, and a top 5 accuracy of 71% and 72%, respectively. Third, Clarifai achieved a top 1 accuracy of 38% and a top 5 accuracy of 64%. Furthermore, IBM Watson Recognition and LogMeal were less accurate than all previously mentioned platforms. Google Vision API achieved the least accurate results, with a top 1 and top 5 accuracy of 9% and 24%, respectively. For Google Vision API, it seemed the recognition was influenced by how well the food or beverage filled the frame. This seems to be the reason Google Vision API appears to perform better under the unspecified angle setting (top 1 accuracy 23%; top 5 accuracy 52%). Under this setting, the majority of the pictures were taken closer to the food or beverage compared with the standard settings. When a food item was relatively small in volume, lying on a plate that was fully in frame, this would result in the food itself filling a small part of the full frame. The latter seemed to be a difficulty for Google Vision API. Among the tested platforms, Google Vision API is not specifically designed for food recognition, contrary to many other APIs and apps tested in this study. Google Vision API would often recognize a plate or dish in the picture but would give no indication as to what was on the plate. IBM Watson Recognition is also not specifically developed for the recognition of food, but they do provide a specific food module if the platform’s general model identifies the picture as a food. Overall, the tested platforms vary hugely in their performance. Due to the variation it remains difficult to recommend a specific platform in the context of dietary assessment.

### Comparison of Settings

Based on the comparison of the different settings among all platforms, the setting influenced both the accuracy and totality. The accuracy achieved in the ideal setting was only slightly higher compared with the ones reached in the nonstandardized containers setting, the setting with clutter in frame, and the badly lit setting. Moreover, the unspecified angle setting resulted in a significantly higher recognition accuracy compared with all other settings. Although unexpected, this is actually a promising result since it means that recognition platforms perform better when supplied with pictures taken at angles and distances that feel natural to the consumer or participant compared with pictures that are taken at a standardized angle and height.

Furthermore, specific differences in recognition accuracy were noticed between platforms and settings. Google Vision API seemed unable to perform well when there was clutter in the frame. Additionally, the nonstandard container setting appeared to pose quite a problem for Google Vision API and LogMeal. On the other hand, Clarifai appeared to struggle most with bad lighting and having clutter in the frame. IBM Watson Recognition had the most difficulties when the lighting was bad or when the food or drink was in a nonstandard container. Bitesnap also appeared to have some problems with bad lighting. Foodvisor seemed to perform the worst in the ideal setting. It is clear that the performance of each platform is influenced by different factors in different ways; potentially the algorithms performing in the back end of these platforms play a key role.

### Comparison of Food Categories

When comparing the recognition accuracy over the different food categories, mixed dish components were found to be recognized with the highest accuracy at the top 1 level. This implies that when multiple dish components are in the frame, often at least one of them can be identified correctly. However, it does not imply that the platforms succeeded in recognizing all dish components. When comparing how well the different platforms could recognize all different components of mixed dishes (totality), Foodvisor and Calorie Mama API were clearly best at recognizing a dish in its totality. Simple processed foods and beverages were recognized better than simple plain foods at the top 1 level but not at top 5 level. Simple processed foods seem to be less variable visually than simple plain foods and are consequently better recognized. Further, it appeared that some platforms simply could not properly recognize certain simple, plain foods, possibly because the food term was not in their system. Another explanation for this difference could be that pictures of simple, processed foods are just more common than pictures of simple, plain foods. However, this is just a hypothesis since it is unknown how each of the image recognition platforms were trained. For beverages, the accuracy remained low.

### Limitations

The main limitation of this study is the use of demo versions of the recognition platforms. Demo versions do show the general capabilities of the platform but could potentially be improved to allow proper dietary assessment. Apart from the technological aspects of the different platforms, there are no specific guidelines for the creation of different settings and circumstances; however, benchmark datasets (eg, ETHZ-Food-101) are quite close to real-life images. As our results show, the setting determines the accuracy of the different platforms. Further, the use of large public available datasets (eg, FoodX-251 or Food-5K) would allow evaluation and testing of the external validity of our findings. Pictures from real-life settings, especially, would be of added value. Our real-life setting tried to assess the real-life recognition accuracy but since we used specifically selected foods and beverages, it can only serve as a surrogate for the real-life situation. However, we had to use this research approach in order to allow for a fair comparison between the different settings. Previously, He et al [17] tested their own image recognition system on 1453 real-life food images taken by 45 free-living or community-dwelling individuals and reached a recognition accuracy of 34%. When they also included contextual information such as food co-occurrence patterns and personalized learning models in the recognition process, the recognition accuracy increased to 44%.

We did not correct for multiple testing because this study was conducted as an exploratory study of the current recognition accuracy of commercial available image recognition platforms.

To our knowledge, this is the first study that compared performances of currently available image recognition platforms with a focus on consumer use. Results were highly variable but promising nonetheless. However, currently there is no freely available recognition system that is already useable for dietary assessment in the context of research. To obtain nutrient intake assessments, food images could be coupled to a food composition database, which translates the food weight into nutritional values. Previously, the lack of a comprehensive food density database was limiting, but this has now been improved [15,24]. Multiple groups are working toward the goal of successful estimation of food amounts [13,18,23]. For example, Rhyner et al [23] worked specifically on a system for automated estimation of carbohydrate content of a meal via image recognition. They tested whether their system could help type 1 diabetes patients in estimating the carbohydrate content of their meals. When patients were asked to calculate the carbohydrate content on their own as they normally would, the authors found a mean absolute error of 27.89 (SD 38.20) g. When the patients were asked to use their system for automated estimation of the carbohydrate content, the mean absolute error was reduced to 12.28 (SD 9.56) g [23]. Furthermore, Zhu et al [13] developed and tested an image recognition system. For weight estimation of the food, they reported mean percentage error rates of 3.4% to 56.4% when tested on pictures of garlic bread and yellow cake. Therefore, we expect that the accurate estimation of food amount is a hurdle that can and will be overcome in the future. Combining image recognition with other sources of information (eg, time of day, container in which the food is in, and location) could be used and gathered using a smartphone. We expect that certain foods or beverages will never be distinguished via image recognition. Hence, when the recognition system identifies multiple foods or beverages with a similar high likelihood (eg, soy milk versus cow’s milk), simply asking the participant which one it is could be the best solution, as suggested by Eldridge et al [25] in their stepwise approach for reporting dietary assessment using different technologies. When required, additional questions could also be asked of the participant (eg, asking the fat content when the system recognizes a drink as being milk). By using a blended assessment form like this, it is expected that the use of image recognition technology for dietary assessment could not only be possible but could solve some of the problems faced by traditional dietary assessment methods.

### Conclusion

To our knowledge, this is the first comparative study investigating the recognition accuracy of currently available image recognition platforms on food and drinks. We found the recognition accuracy to vary widely between different platforms ranging from poor to excellent. The estimation of portion sizes of foods or beverages is lacking in the tested platforms. A blended form of assessment, automated image recognition, and asking questions using chatbot options could improve the overall dietary assessment.

## Appendix

Multimedia Appendix 1 Food and drink items to be used.

Multimedia Appendix 2 Food images in different settings.

Multimedia Appendix 3 Mixed dishes composition.

## Abbreviations

API: application programming interface
EIT: European Institute of Innovation and Technology
