# Supplementary material for: Use of Different Food Image Recognition Platforms in Dietary Assessment: Comparison Study
Source: JMIR Form Res. 2020 Dec 7;4(12):e15602. doi: 10.2196/15602 (PMC7752530; doi:10.2196/15602)
Supplement: Multimedia Appendix 1 [file formative_v4i12e15602_app1.docx]

**Multimedia Appendix 1.** Food and drink items to be used.

5 simple plain foods:

- A carrot, whole
- An apple cut into chunks
- A mango, whole
- Cauliflower florets
- A celery stick

5 simple, processed foods:

- A snickers bar (with and without the wrapping)
- Vanilla pudding
- Tortilla chips
- A drumstick
- A croissant

drinks

- Soy milk
- Cow’s milk
- Coke (regular; in a glass and in its can)
- Water
- Black coffee
- Tea
- Beer

12 mixed dishes:

- Beef stew with mashed potatoes
- Pork ham with green beans, boiled potatoes and mustard sauce
- Chicken tikka masala with rice
- Caesar salad
- Tomato soup
- Pancakes with syrup
- Quiche Lorraine
- Ravioli in tomato sauce
- Linguini with ham, mushrooms and cream sauce
- Pizza Quattro Formaggi
- Breakfast cereal with milk
- Sandwich with chocolate spread
