# Supplementary figures and images for: Use of Different Food Image Recognition Platforms in Dietary Assessment: Comparison Study (part 2 of 2)
Source: JMIR Form Res. 2020 Dec 7;4(12):e15602. doi: 10.2196/15602 (PMC7752530; doi:10.2196/15602)

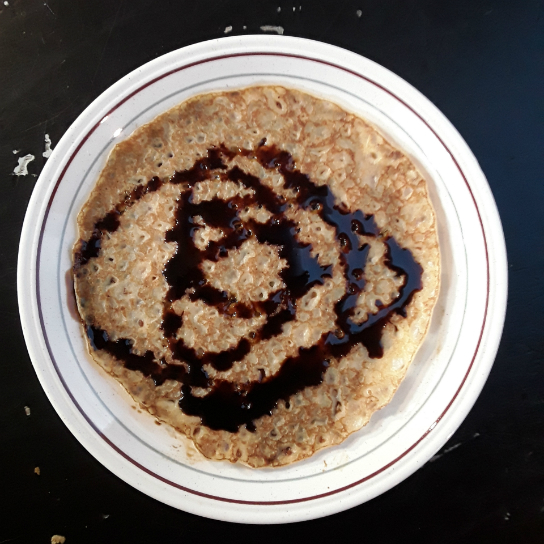

Supplement: Multimedia Appendix 2 [file formative_v4i12e15602_app2.zip › R Pancakes real life.jpg]

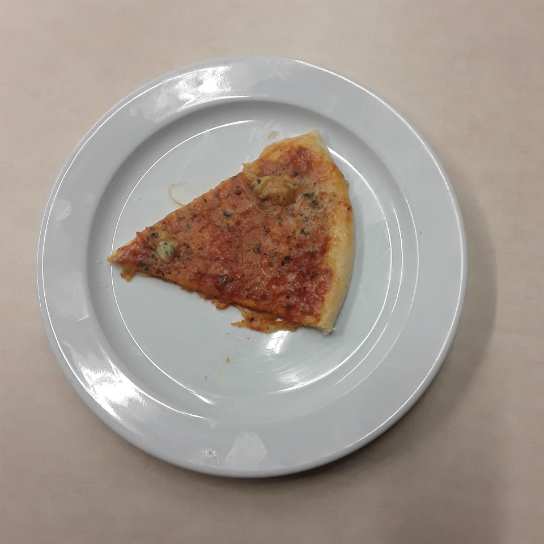

Supplement: Multimedia Appendix 2 [file formative_v4i12e15602_app2.zip › R Pizza angle.jpg]

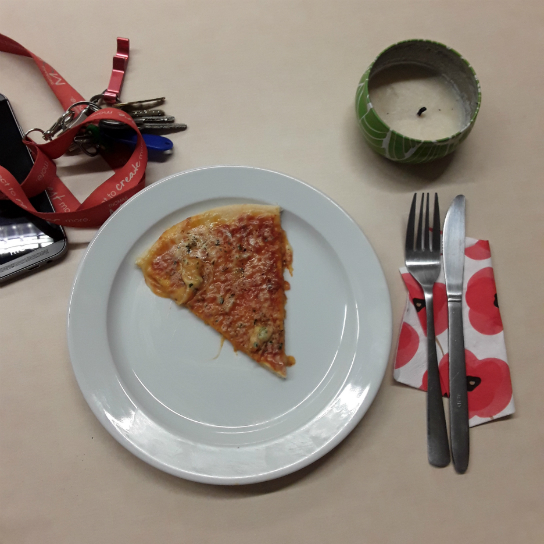

Supplement: Multimedia Appendix 2 [file formative_v4i12e15602_app2.zip › R Pizza clutter.jpg]

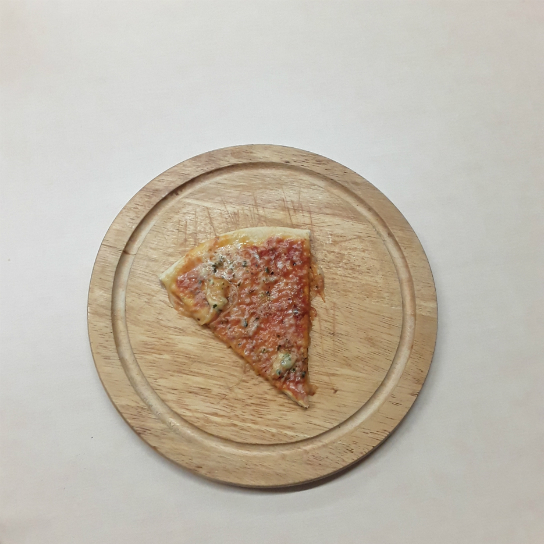

Supplement: Multimedia Appendix 2 [file formative_v4i12e15602_app2.zip › R Pizza container.jpg]

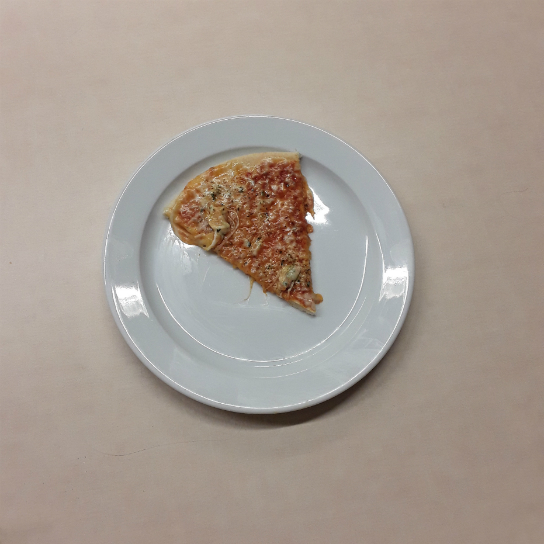

Supplement: Multimedia Appendix 2 [file formative_v4i12e15602_app2.zip › R Pizza ideal.jpg]

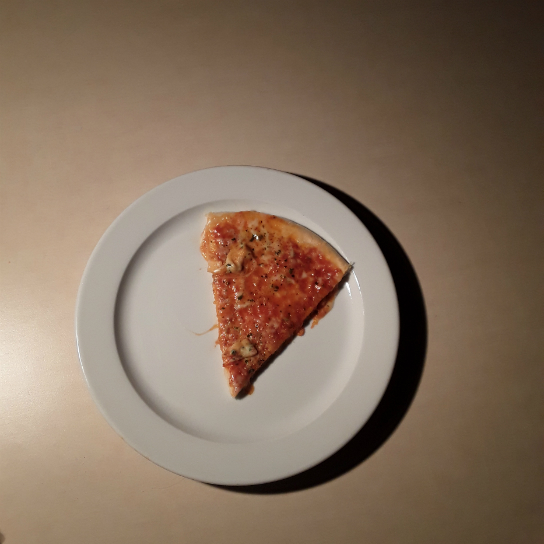

Supplement: Multimedia Appendix 2 [file formative_v4i12e15602_app2.zip › R Pizza light.jpg]

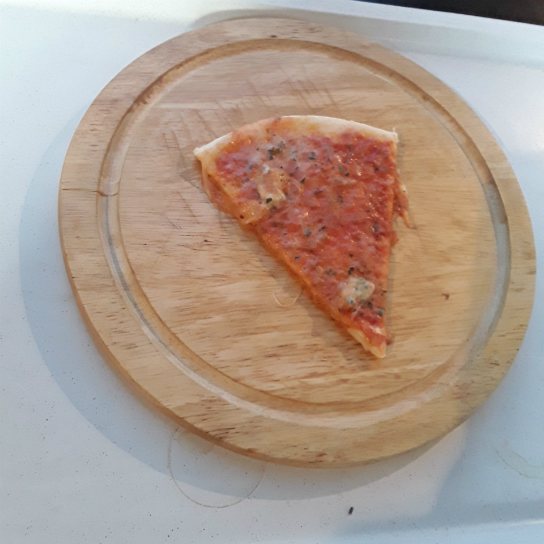

Supplement: Multimedia Appendix 2 [file formative_v4i12e15602_app2.zip › R Pizza real life.jpg]

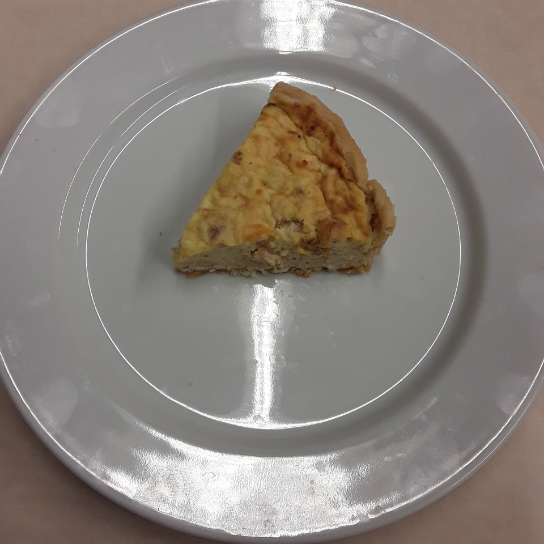

Supplement: Multimedia Appendix 2 [file formative_v4i12e15602_app2.zip › R Quiche angle.jpg]

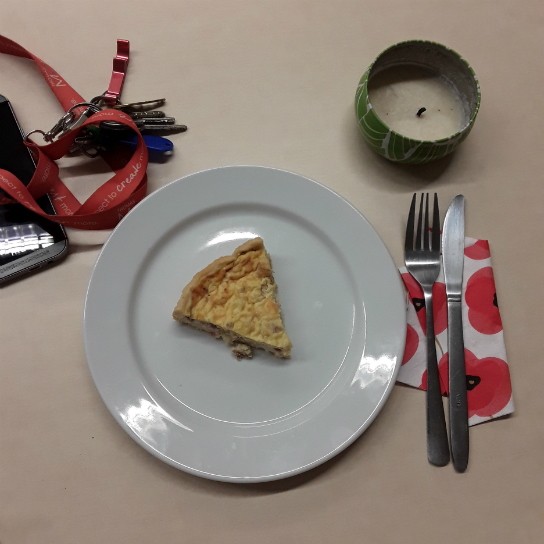

Supplement: Multimedia Appendix 2 [file formative_v4i12e15602_app2.zip › R Quiche clutter.jpg]

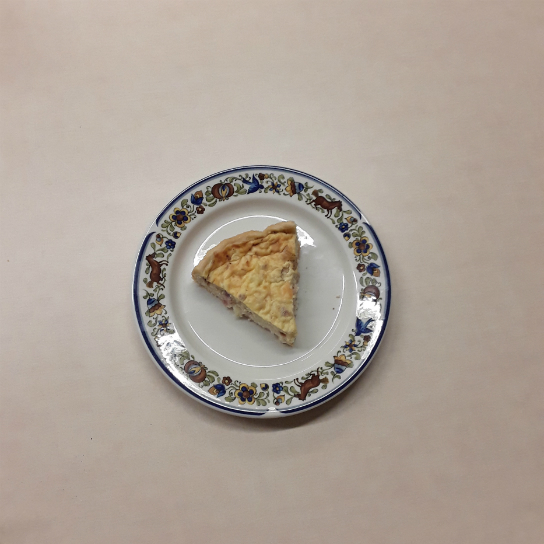

Supplement: Multimedia Appendix 2 [file formative_v4i12e15602_app2.zip › R Quiche container.jpg]

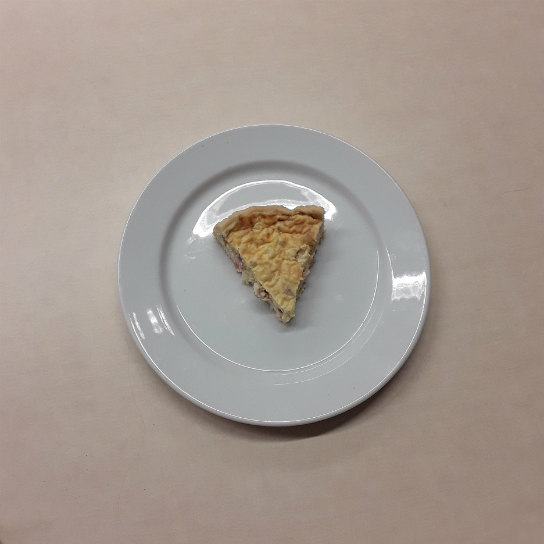

Supplement: Multimedia Appendix 2 [file formative_v4i12e15602_app2.zip › R Quiche ideal.jpg]

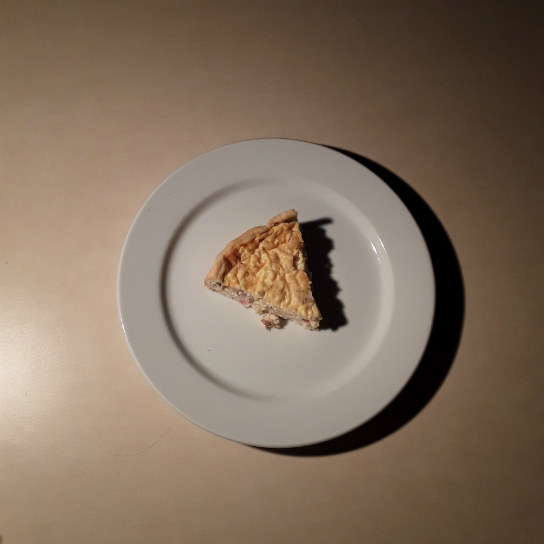

Supplement: Multimedia Appendix 2 [file formative_v4i12e15602_app2.zip › R Quiche light.jpg]

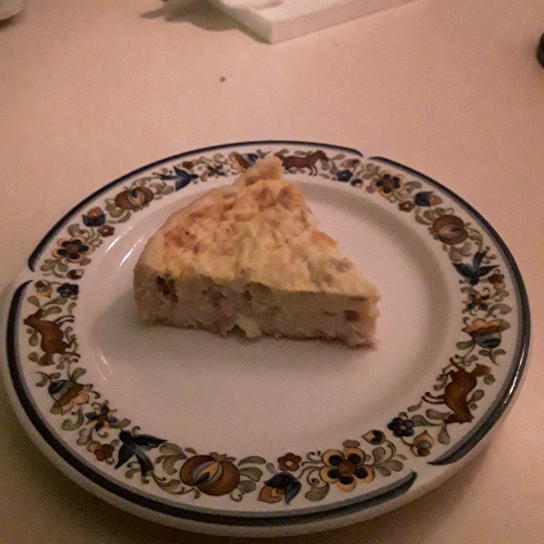

Supplement: Multimedia Appendix 2 [file formative_v4i12e15602_app2.zip › R Quiche real life.jpg]

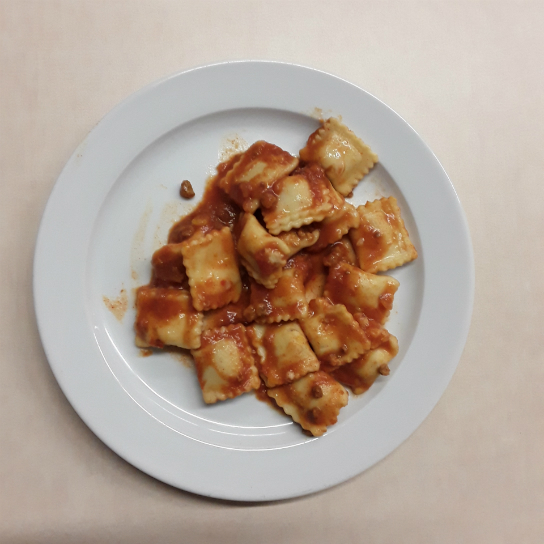

Supplement: Multimedia Appendix 2 [file formative_v4i12e15602_app2.zip › R Ravioli angle.jpg]

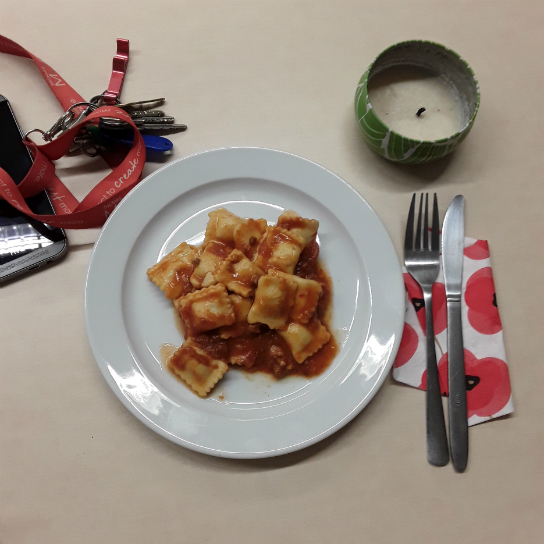

Supplement: Multimedia Appendix 2 [file formative_v4i12e15602_app2.zip › R Ravioli clutter.jpg]

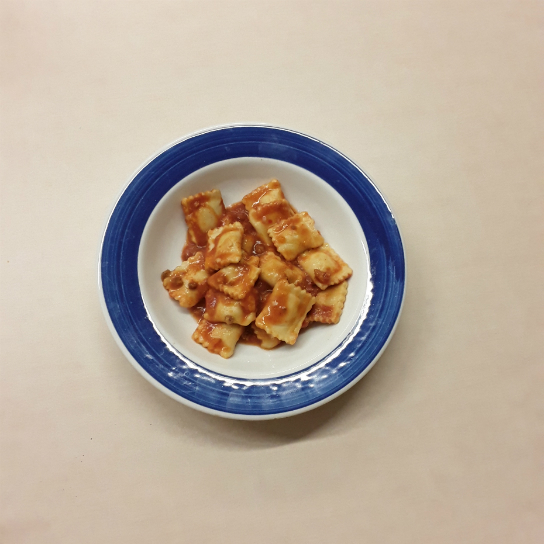

Supplement: Multimedia Appendix 2 [file formative_v4i12e15602_app2.zip › R Ravioli container.jpg]

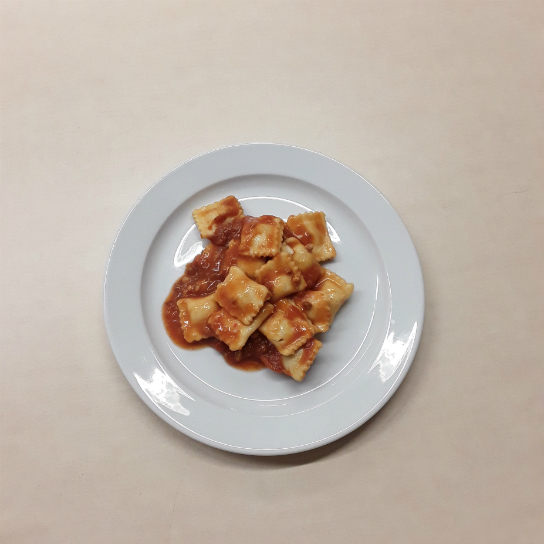

Supplement: Multimedia Appendix 2 [file formative_v4i12e15602_app2.zip › R Ravioli ideal.jpg]

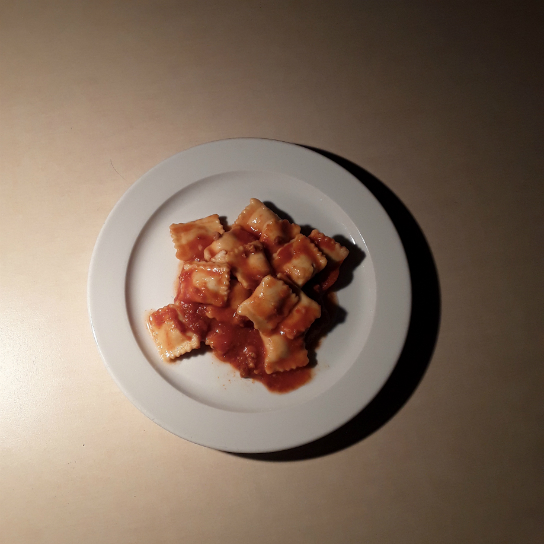

Supplement: Multimedia Appendix 2 [file formative_v4i12e15602_app2.zip › R Ravioli light.jpg]

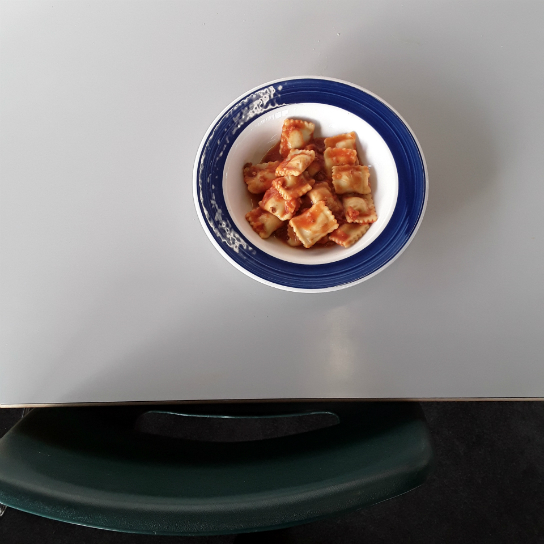

Supplement: Multimedia Appendix 2 [file formative_v4i12e15602_app2.zip › R Ravioli real life.jpg]

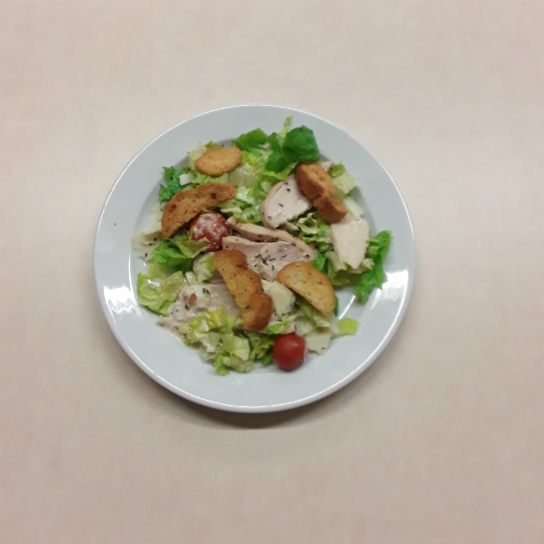

Supplement: Multimedia Appendix 2 [file formative_v4i12e15602_app2.zip › R Salad angle.jpg]

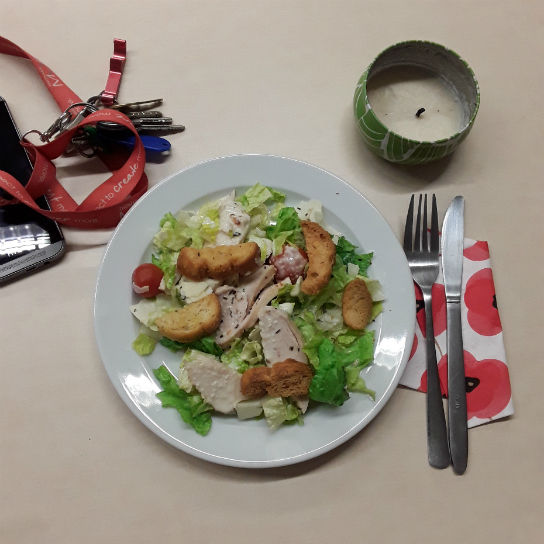

Supplement: Multimedia Appendix 2 [file formative_v4i12e15602_app2.zip › R Salad clutter.jpg]

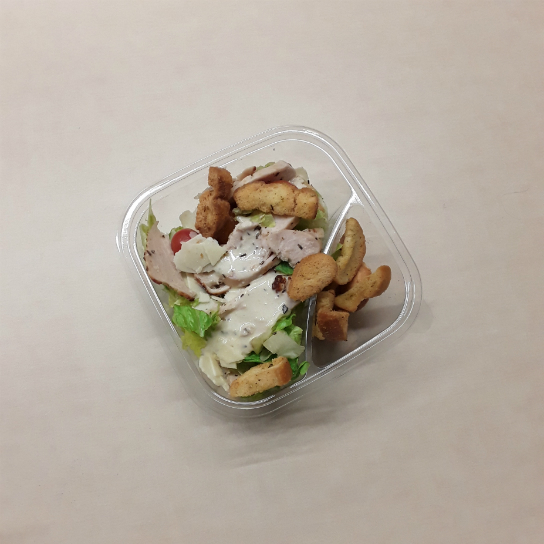

Supplement: Multimedia Appendix 2 [file formative_v4i12e15602_app2.zip › R Salad container.jpg]

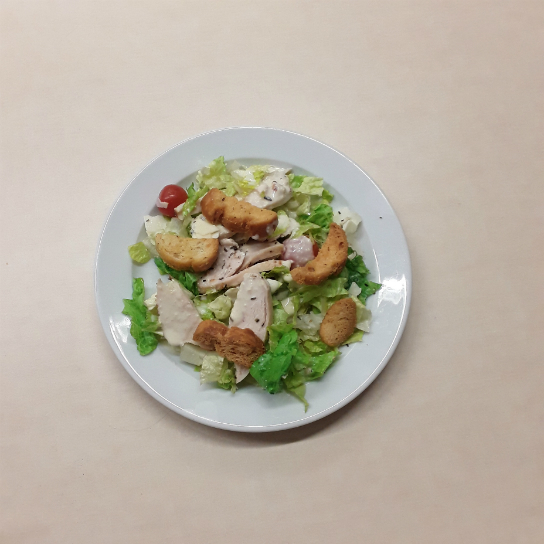

Supplement: Multimedia Appendix 2 [file formative_v4i12e15602_app2.zip › R Salad ideal.jpg]

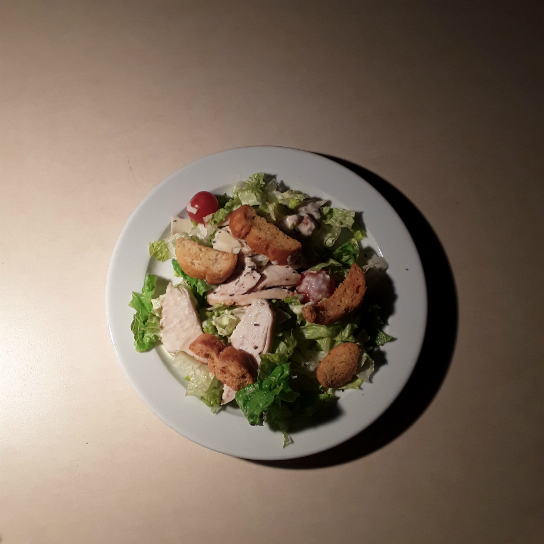

Supplement: Multimedia Appendix 2 [file formative_v4i12e15602_app2.zip › R Salad light.jpg]

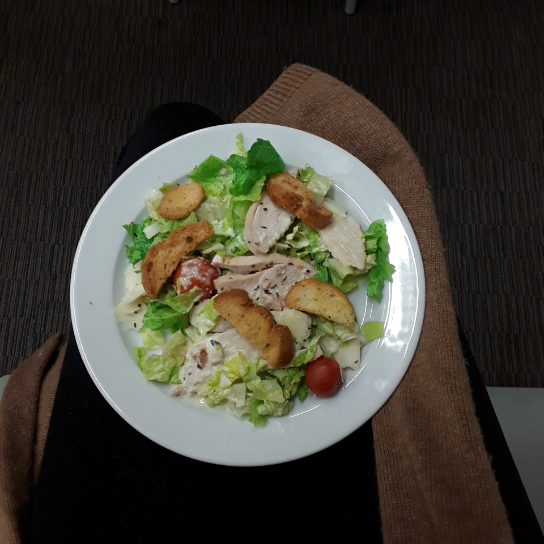

Supplement: Multimedia Appendix 2 [file formative_v4i12e15602_app2.zip › R Salad real life.jpg]

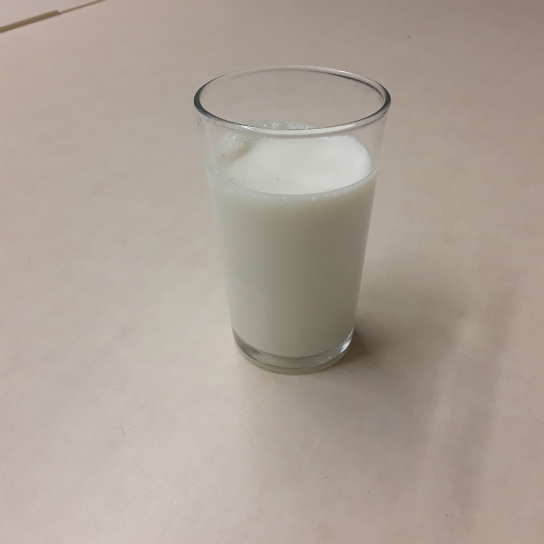

Supplement: Multimedia Appendix 2 [file formative_v4i12e15602_app2.zip › R Semi-skimmed milk angle.jpg]

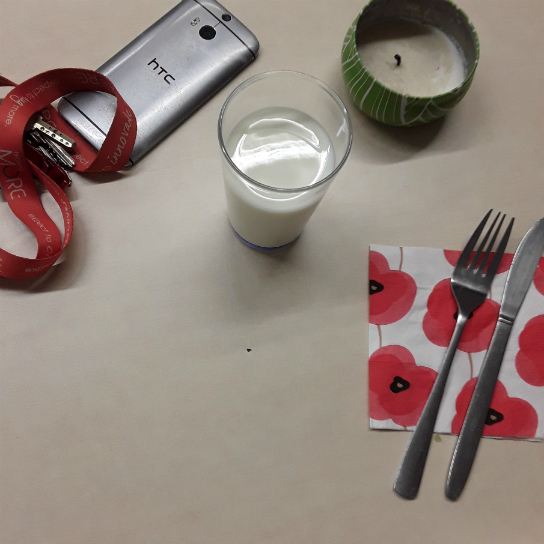

Supplement: Multimedia Appendix 2 [file formative_v4i12e15602_app2.zip › R Semi-skimmed milk clutter.jpg]

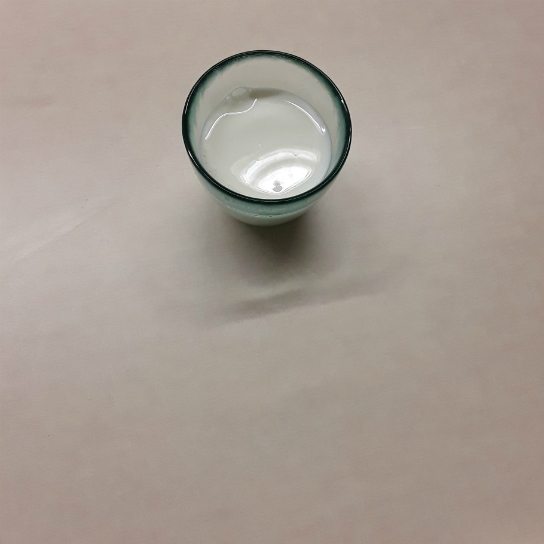

Supplement: Multimedia Appendix 2 [file formative_v4i12e15602_app2.zip › R Semi-skimmed milk container.jpg]

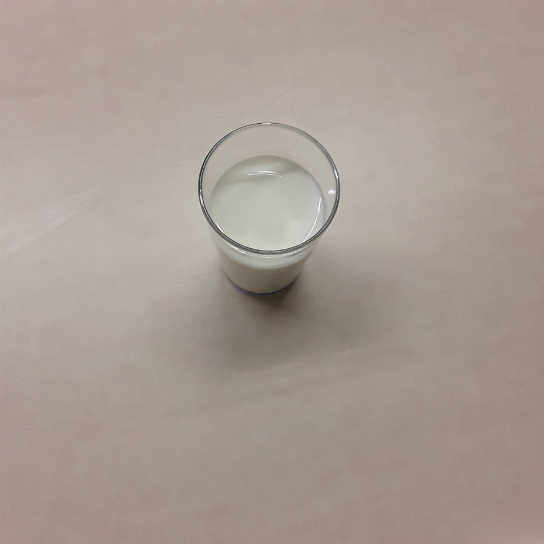

Supplement: Multimedia Appendix 2 [file formative_v4i12e15602_app2.zip › R Semi-skimmed milk ideal.jpg]

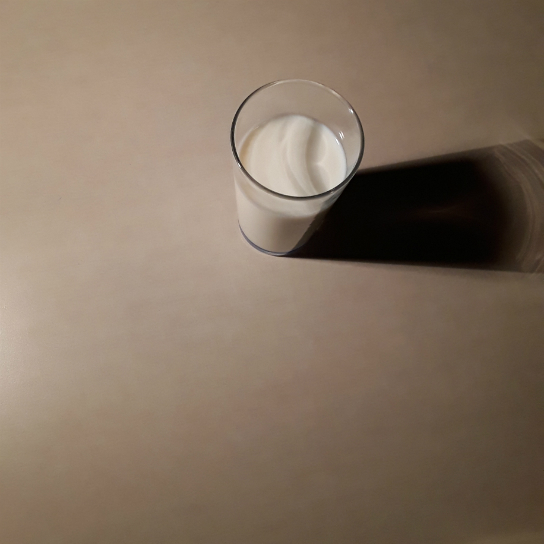

Supplement: Multimedia Appendix 2 [file formative_v4i12e15602_app2.zip › R Semi-skimmed milk light.jpg]

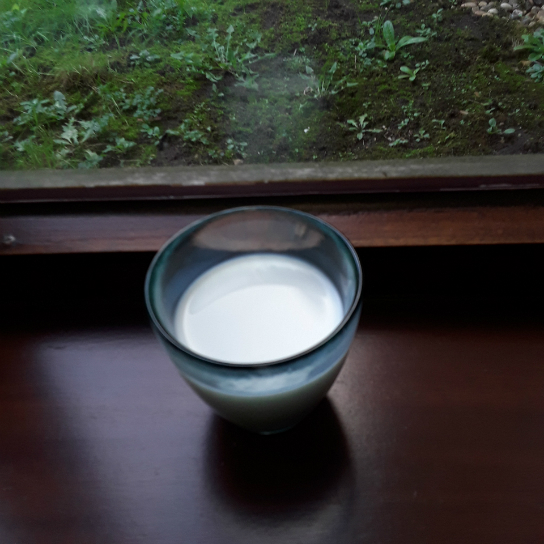

Supplement: Multimedia Appendix 2 [file formative_v4i12e15602_app2.zip › R Semi-skimmed milk real life.jpg]

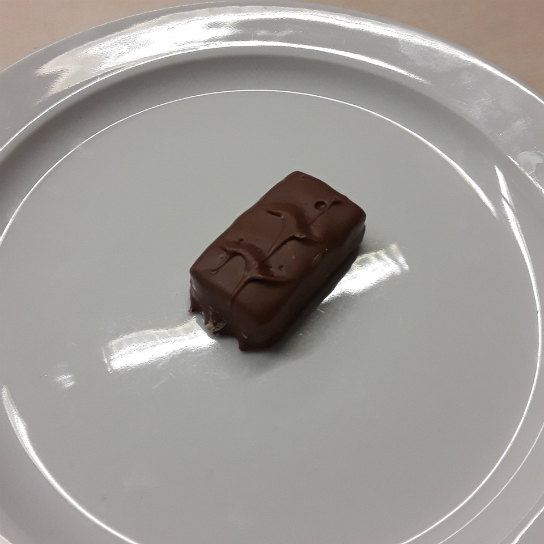

Supplement: Multimedia Appendix 2 [file formative_v4i12e15602_app2.zip › R Snickers angle.jpg]

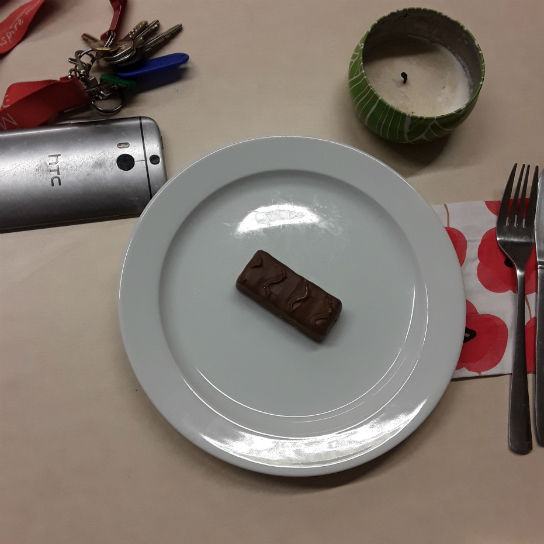

Supplement: Multimedia Appendix 2 [file formative_v4i12e15602_app2.zip › R Snickers clutter.jpg]

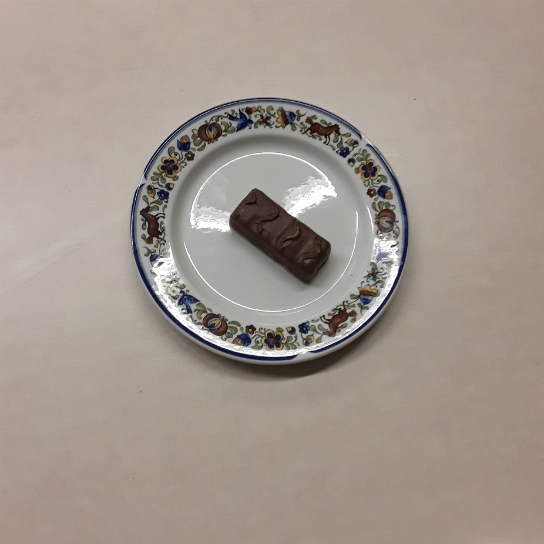

Supplement: Multimedia Appendix 2 [file formative_v4i12e15602_app2.zip › R Snickers container.jpg]

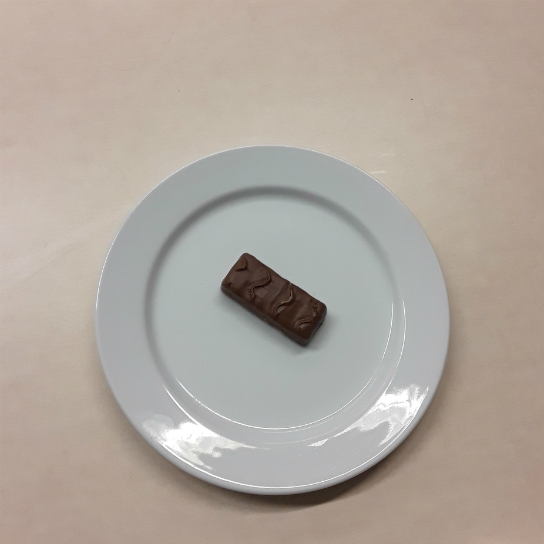

Supplement: Multimedia Appendix 2 [file formative_v4i12e15602_app2.zip › R Snickers ideal.jpg]

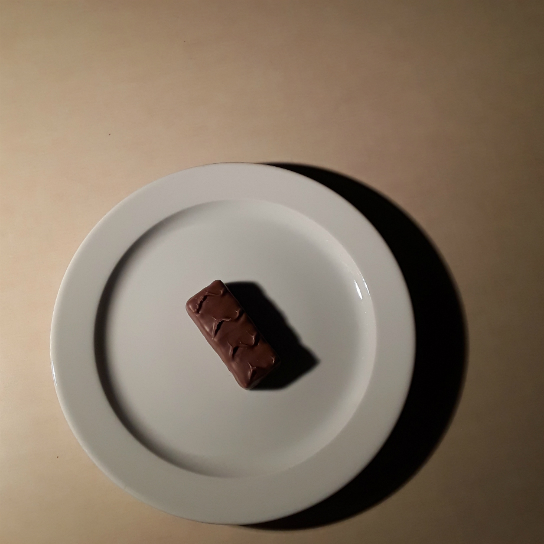

Supplement: Multimedia Appendix 2 [file formative_v4i12e15602_app2.zip › R Snickers light.jpg]

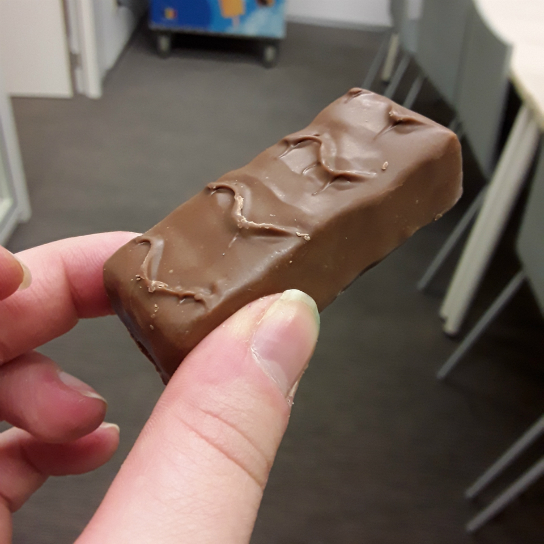

Supplement: Multimedia Appendix 2 [file formative_v4i12e15602_app2.zip › R Snickers real life.jpg]

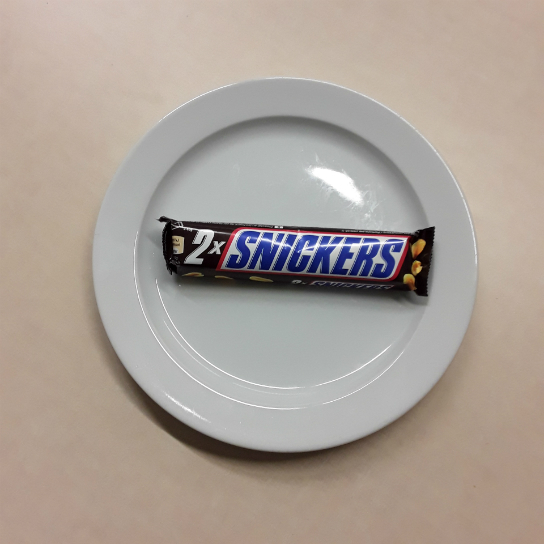

Supplement: Multimedia Appendix 2 [file formative_v4i12e15602_app2.zip › R Snickers with wrapping angle.jpg]

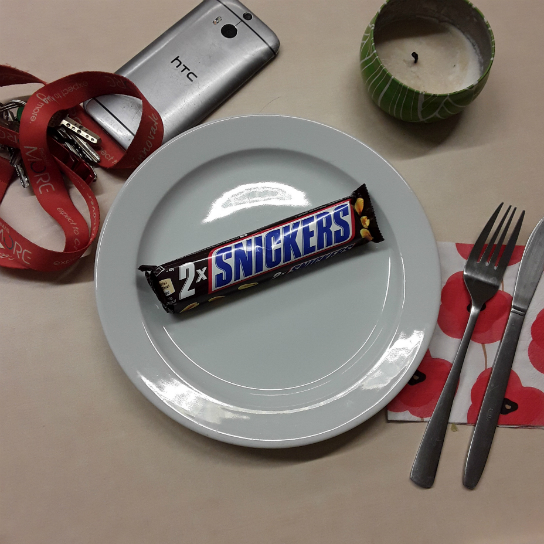

Supplement: Multimedia Appendix 2 [file formative_v4i12e15602_app2.zip › R Snickers with wrapping clutter.jpg]

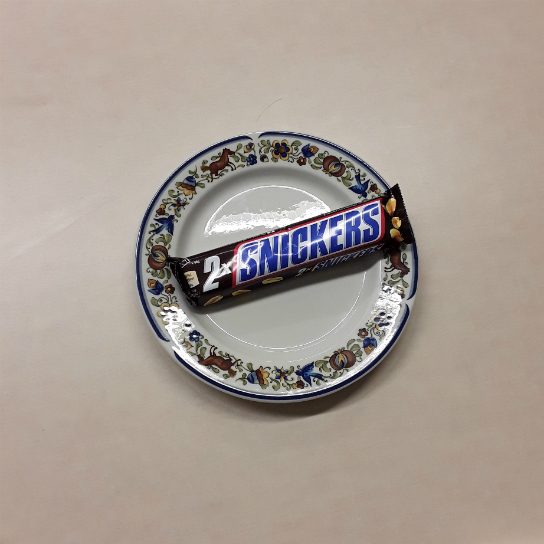

Supplement: Multimedia Appendix 2 [file formative_v4i12e15602_app2.zip › R Snickers with wrapping container.jpg]

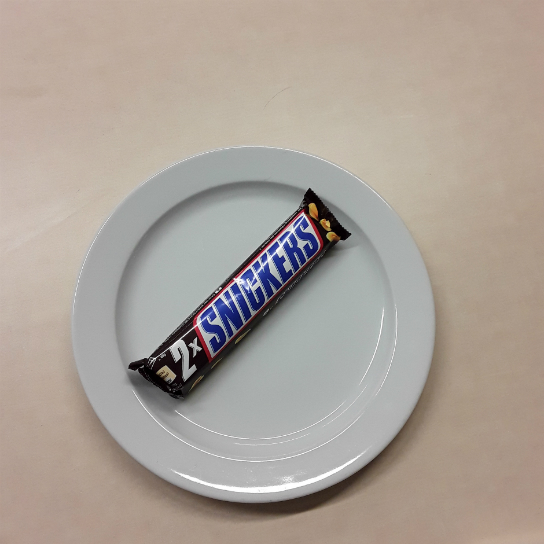

Supplement: Multimedia Appendix 2 [file formative_v4i12e15602_app2.zip › R Snickers with wrapping ideal.jpg]

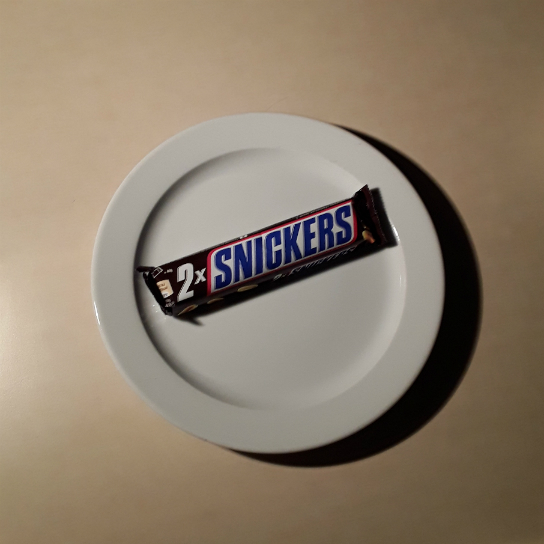

Supplement: Multimedia Appendix 2 [file formative_v4i12e15602_app2.zip › R Snickers with wrapping light.jpg]

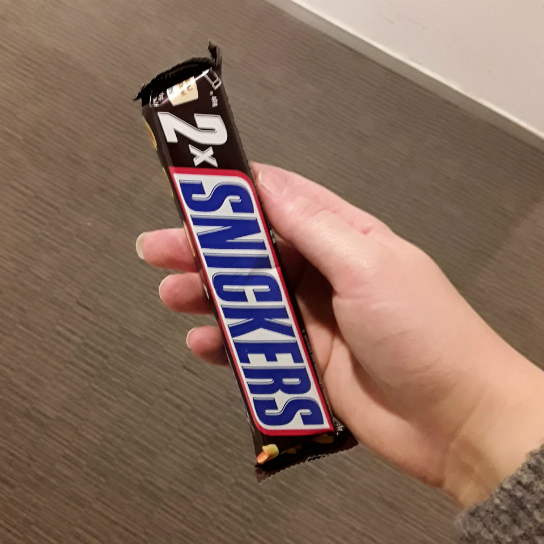

Supplement: Multimedia Appendix 2 [file formative_v4i12e15602_app2.zip › R Snickers with wrapping real life.jpg]

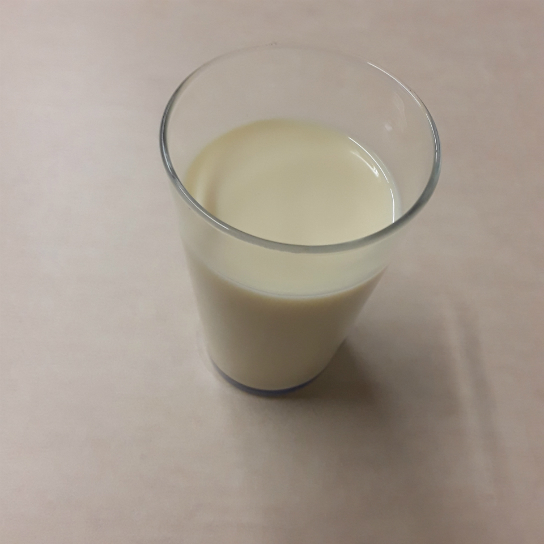

Supplement: Multimedia Appendix 2 [file formative_v4i12e15602_app2.zip › R Soy milk angle.jpg]

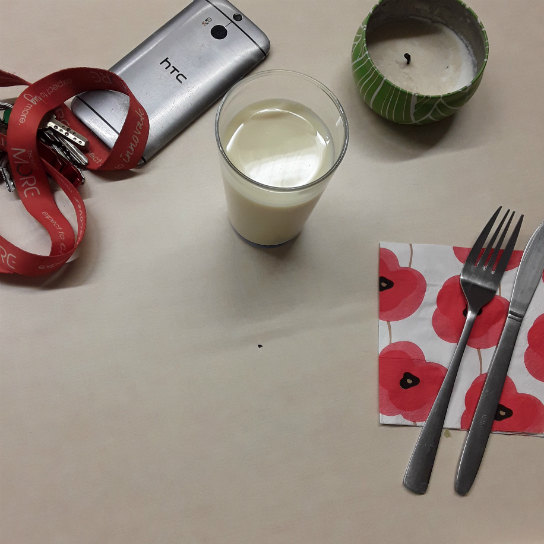

Supplement: Multimedia Appendix 2 [file formative_v4i12e15602_app2.zip › R Soy milk clutter.jpg]

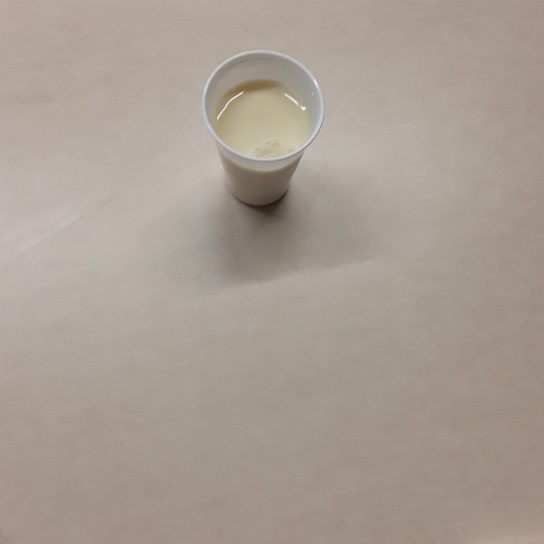

Supplement: Multimedia Appendix 2 [file formative_v4i12e15602_app2.zip › R Soy milk container.jpg]

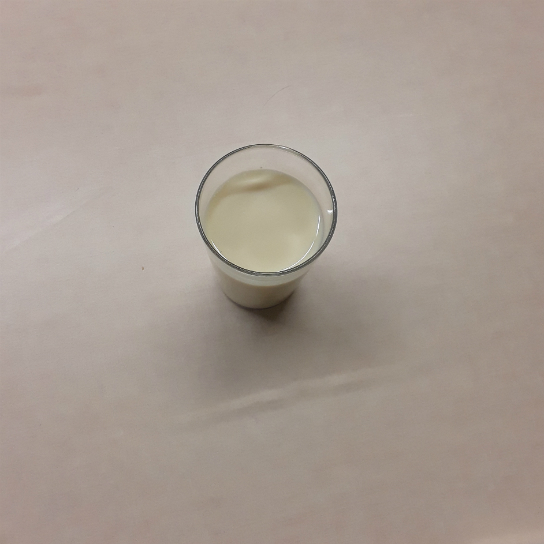

Supplement: Multimedia Appendix 2 [file formative_v4i12e15602_app2.zip › R Soy milk ideal.jpg]

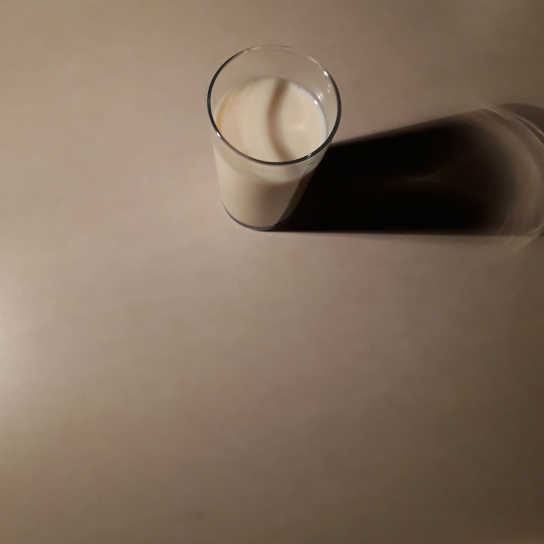

Supplement: Multimedia Appendix 2 [file formative_v4i12e15602_app2.zip › R Soy milk light.jpg]

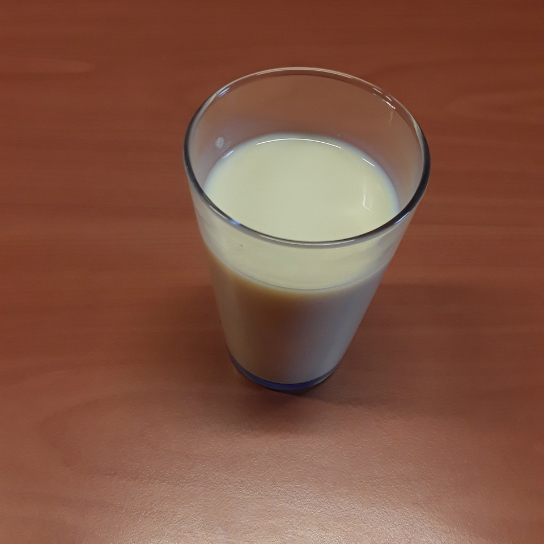

Supplement: Multimedia Appendix 2 [file formative_v4i12e15602_app2.zip › R Soy milk real life.jpg]

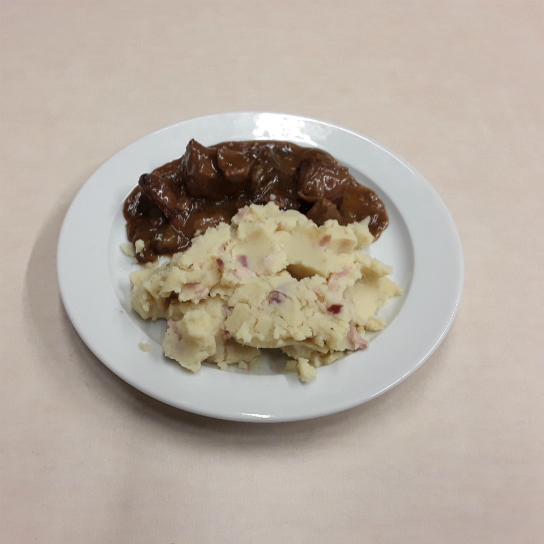

Supplement: Multimedia Appendix 2 [file formative_v4i12e15602_app2.zip › R Stew angle.jpg]

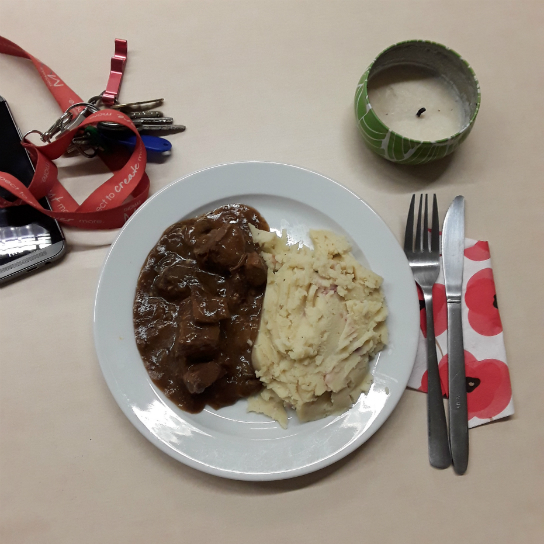

Supplement: Multimedia Appendix 2 [file formative_v4i12e15602_app2.zip › R Stew clutter.jpg]

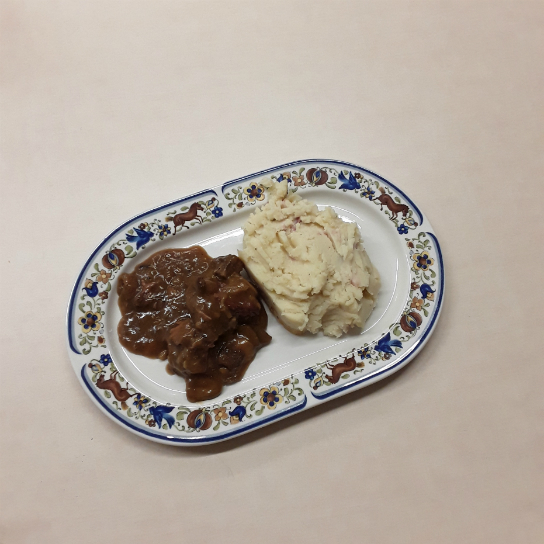

Supplement: Multimedia Appendix 2 [file formative_v4i12e15602_app2.zip › R Stew container.jpg]

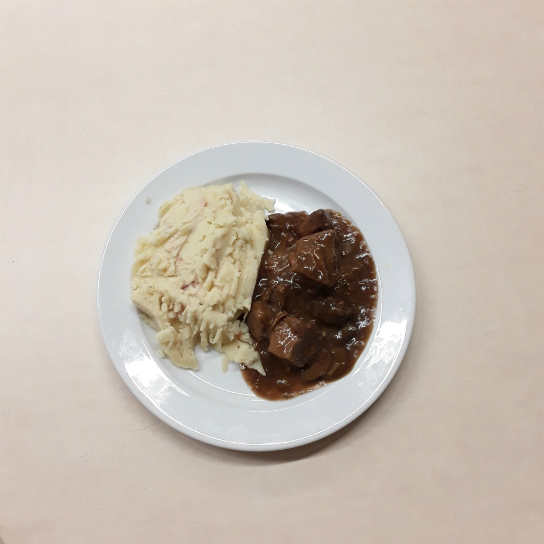

Supplement: Multimedia Appendix 2 [file formative_v4i12e15602_app2.zip › R Stew ideal.jpg]

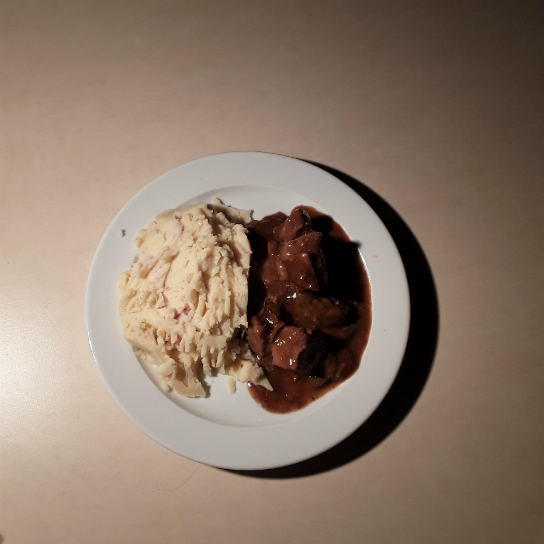

Supplement: Multimedia Appendix 2 [file formative_v4i12e15602_app2.zip › R Stew light.jpg]

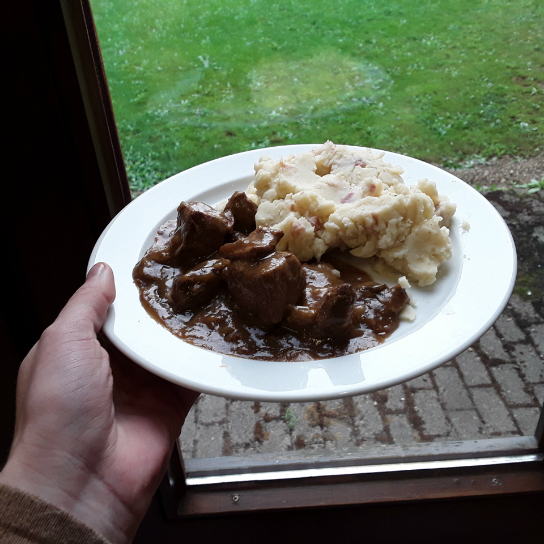

Supplement: Multimedia Appendix 2 [file formative_v4i12e15602_app2.zip › R Stew real life.jpg]

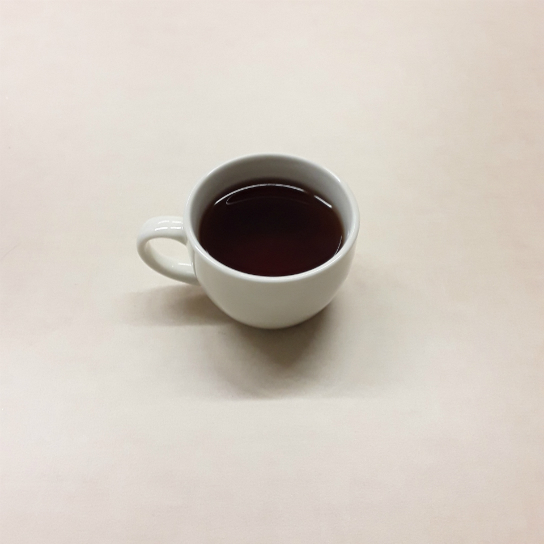

Supplement: Multimedia Appendix 2 [file formative_v4i12e15602_app2.zip › R Tea angle.jpg]

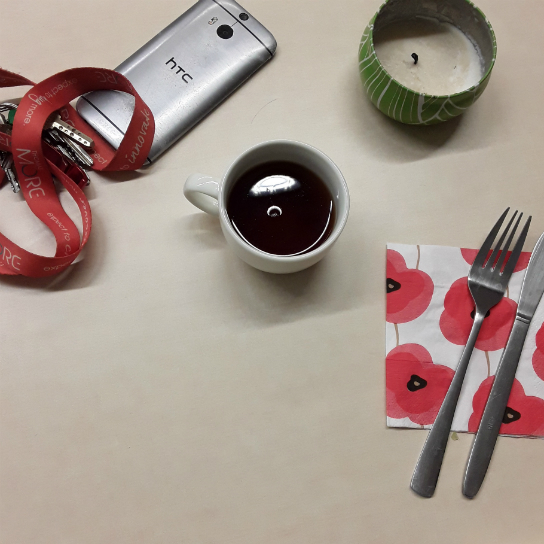

Supplement: Multimedia Appendix 2 [file formative_v4i12e15602_app2.zip › R Tea clutter.jpg]

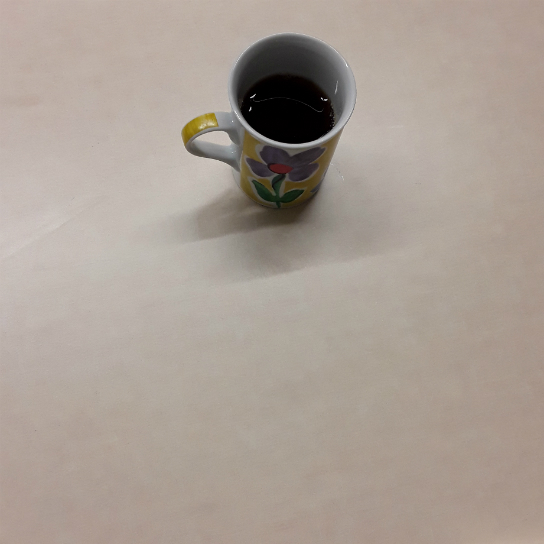

Supplement: Multimedia Appendix 2 [file formative_v4i12e15602_app2.zip › R Tea container.jpg]

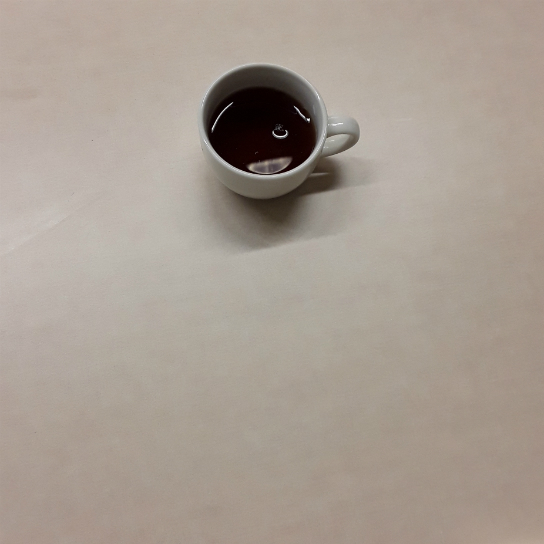

Supplement: Multimedia Appendix 2 [file formative_v4i12e15602_app2.zip › R Tea ideal.jpg]

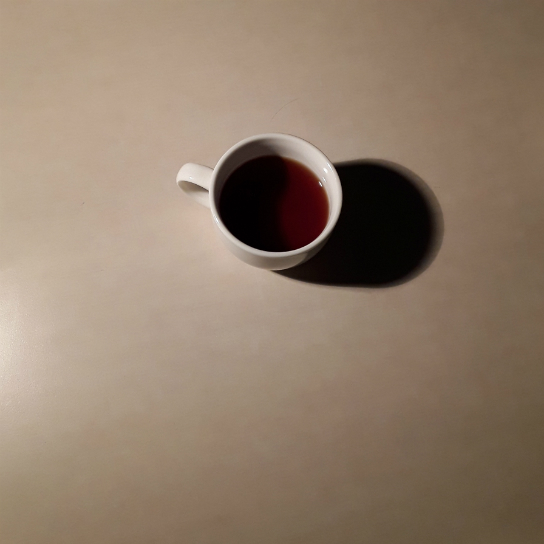

Supplement: Multimedia Appendix 2 [file formative_v4i12e15602_app2.zip › R Tea light.jpg]

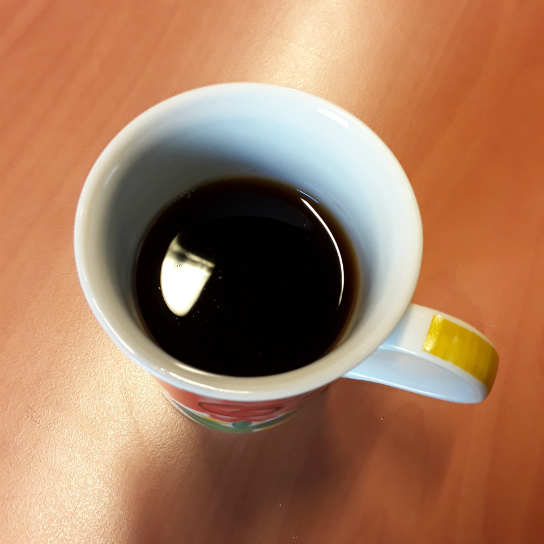

Supplement: Multimedia Appendix 2 [file formative_v4i12e15602_app2.zip › R Tea real life.jpg]

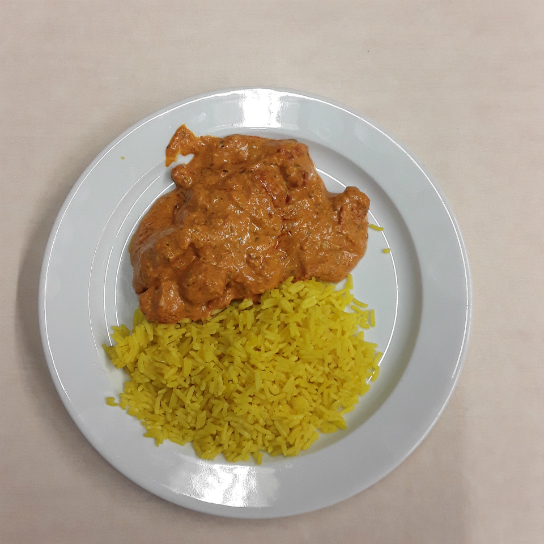

Supplement: Multimedia Appendix 2 [file formative_v4i12e15602_app2.zip › R Tikka masala angle.jpg]

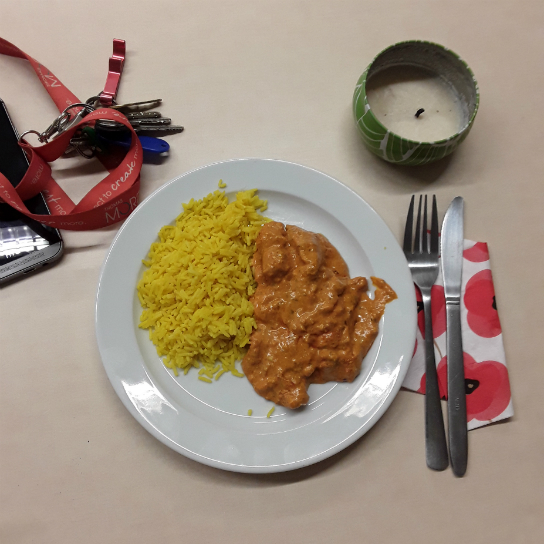

Supplement: Multimedia Appendix 2 [file formative_v4i12e15602_app2.zip › R Tikka masala clutter.jpg]

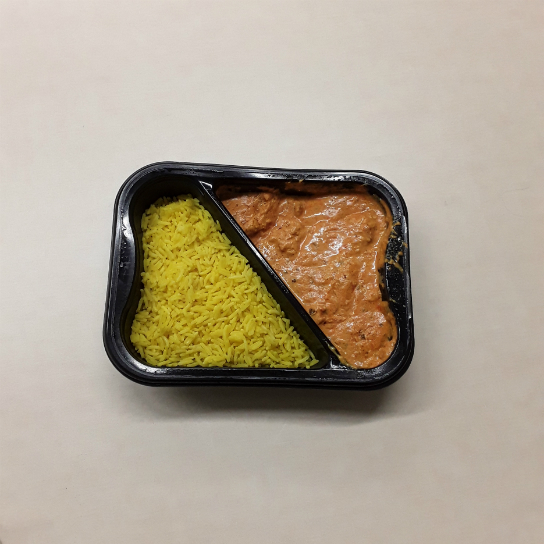

Supplement: Multimedia Appendix 2 [file formative_v4i12e15602_app2.zip › R Tikka masala container.jpg]

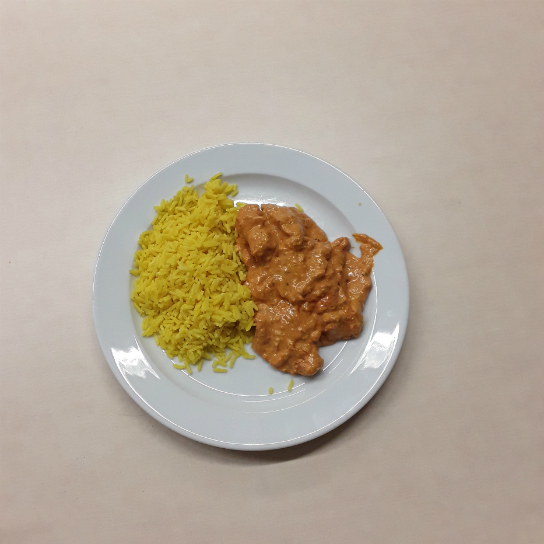

Supplement: Multimedia Appendix 2 [file formative_v4i12e15602_app2.zip › R Tikka masala ideal.jpg]

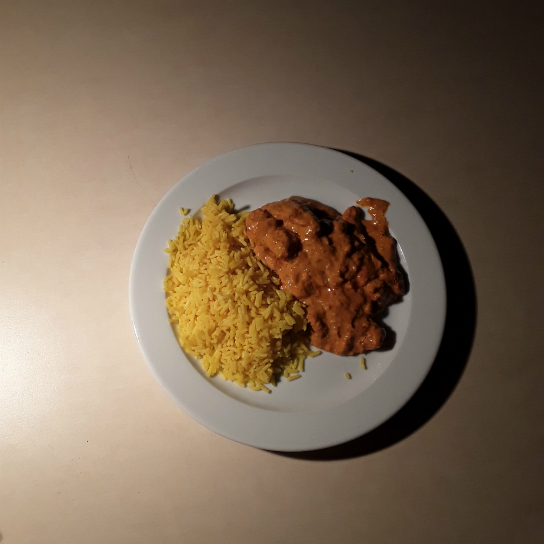

Supplement: Multimedia Appendix 2 [file formative_v4i12e15602_app2.zip › R Tikka masala light.jpg]

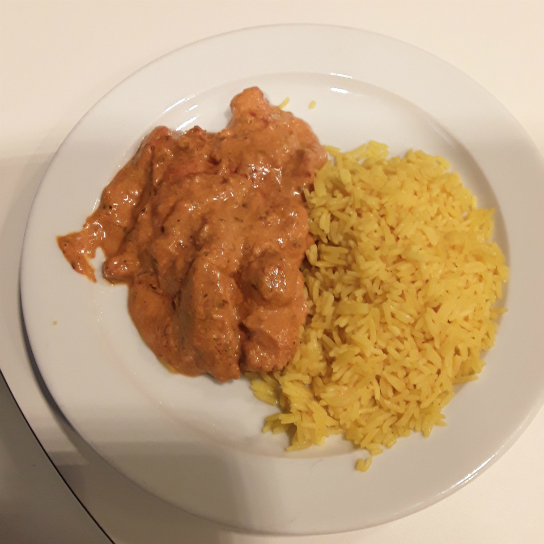

Supplement: Multimedia Appendix 2 [file formative_v4i12e15602_app2.zip › R Tikka masala real life.jpg]

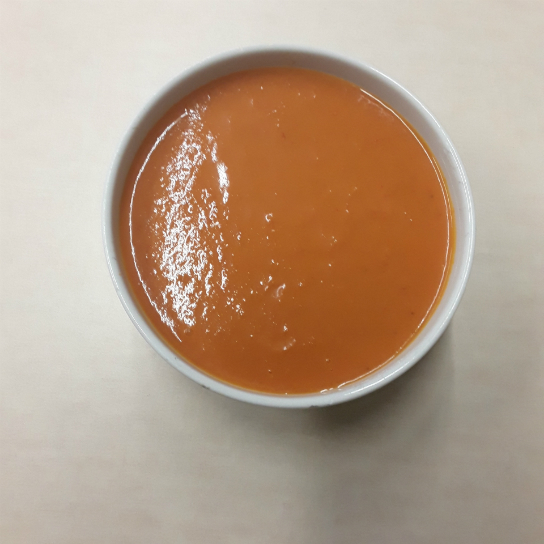

Supplement: Multimedia Appendix 2 [file formative_v4i12e15602_app2.zip › R Tomatosoup angle.jpg]

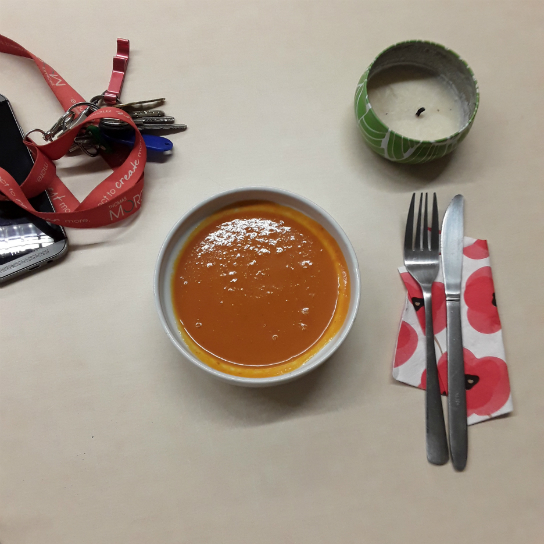

Supplement: Multimedia Appendix 2 [file formative_v4i12e15602_app2.zip › R Tomatosoup clutter.jpg]

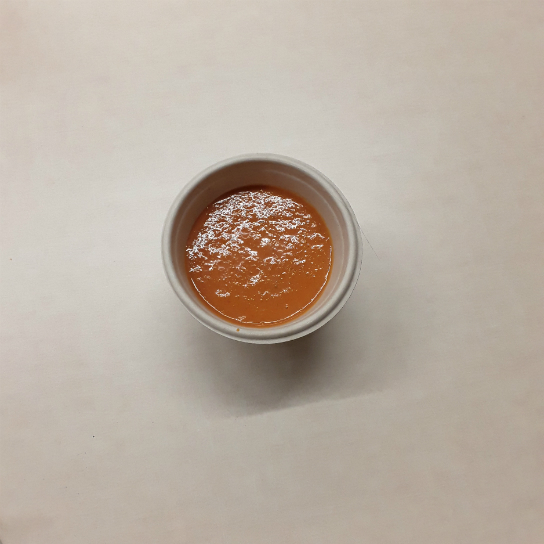

Supplement: Multimedia Appendix 2 [file formative_v4i12e15602_app2.zip › R Tomatosoup container.jpg]

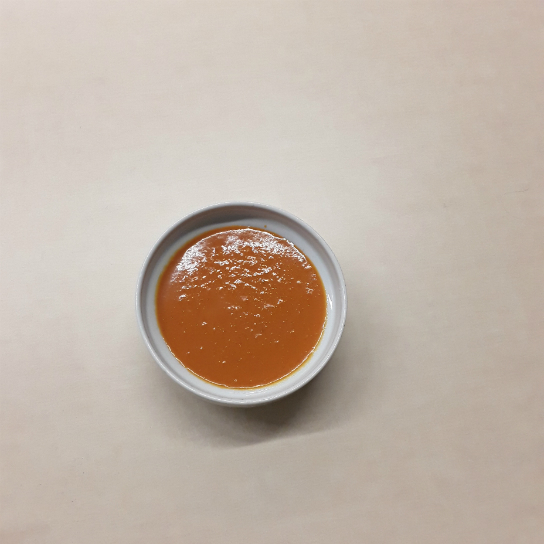

Supplement: Multimedia Appendix 2 [file formative_v4i12e15602_app2.zip › R Tomatosoup ideal.jpg]

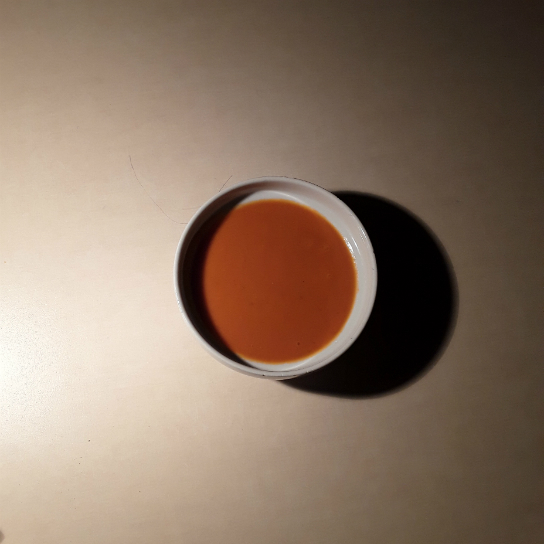

Supplement: Multimedia Appendix 2 [file formative_v4i12e15602_app2.zip › R Tomatosoup light.jpg]

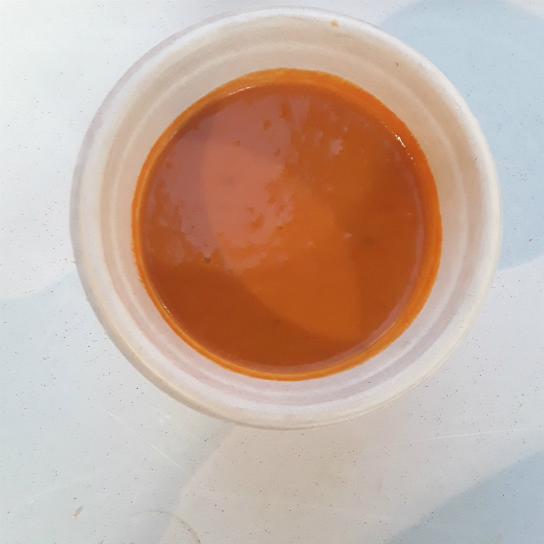

Supplement: Multimedia Appendix 2 [file formative_v4i12e15602_app2.zip › R Tomatosoup real life.jpg]

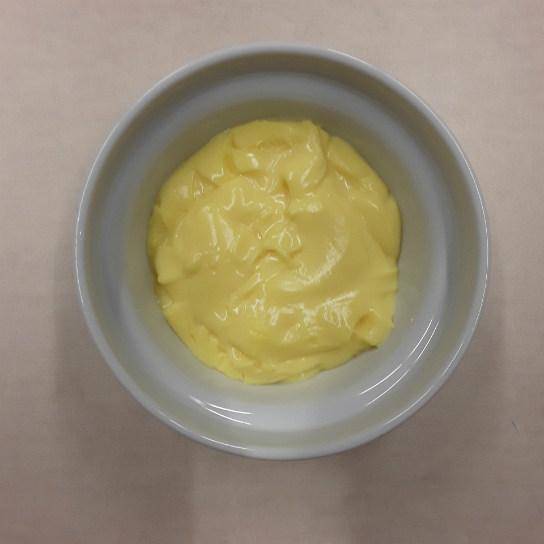

Supplement: Multimedia Appendix 2 [file formative_v4i12e15602_app2.zip › R Vanilla pudding angle.jpg]

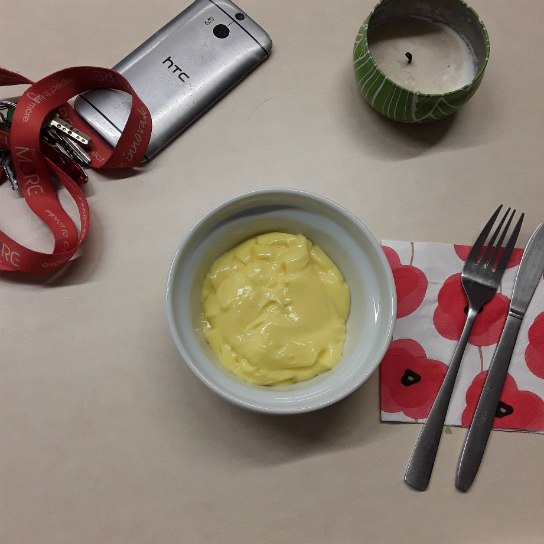

Supplement: Multimedia Appendix 2 [file formative_v4i12e15602_app2.zip › R Vanilla pudding clutter.jpg]

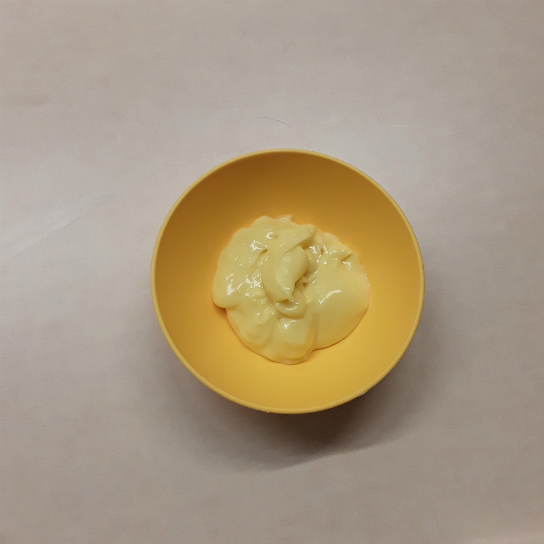

Supplement: Multimedia Appendix 2 [file formative_v4i12e15602_app2.zip › R Vanilla pudding container.jpg]

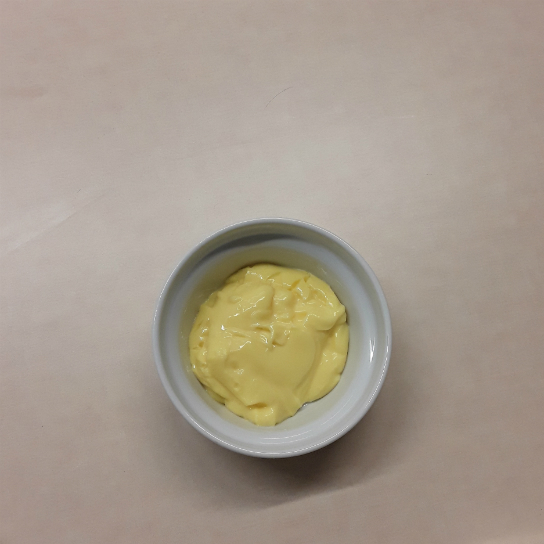

Supplement: Multimedia Appendix 2 [file formative_v4i12e15602_app2.zip › R Vanilla pudding ideal.jpg]

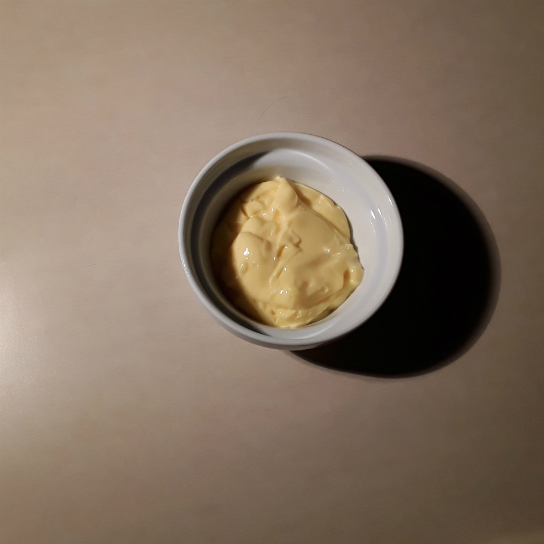

Supplement: Multimedia Appendix 2 [file formative_v4i12e15602_app2.zip › R Vanilla pudding light.jpg]

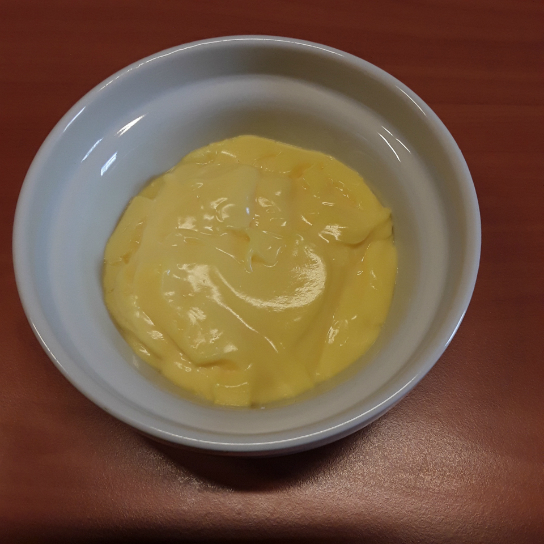

Supplement: Multimedia Appendix 2 [file formative_v4i12e15602_app2.zip › R Vanilla pudding real life.jpg]

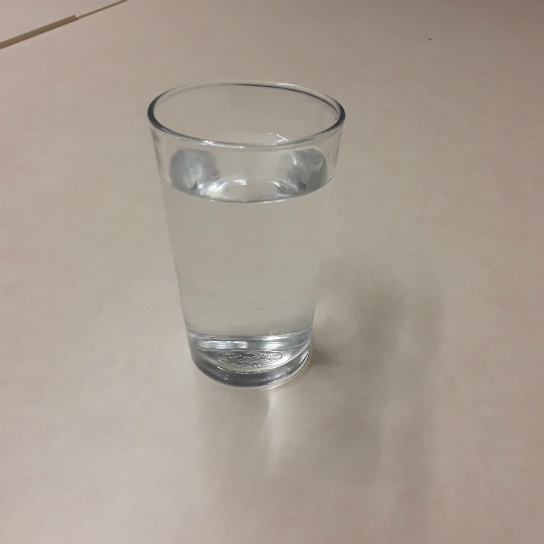

Supplement: Multimedia Appendix 2 [file formative_v4i12e15602_app2.zip › R Water angle.jpg]

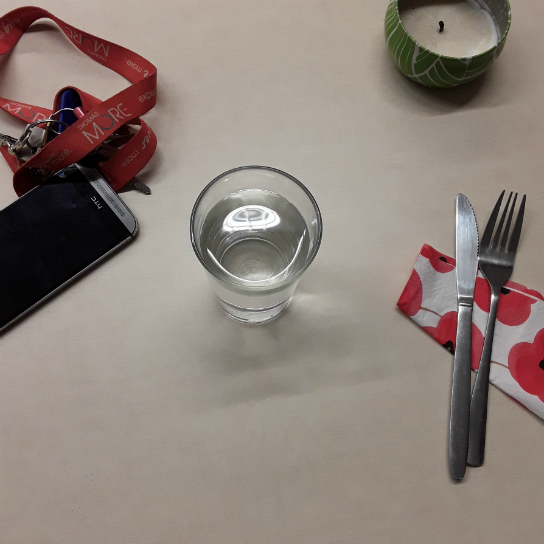

Supplement: Multimedia Appendix 2 [file formative_v4i12e15602_app2.zip › R Water clutter.jpg]

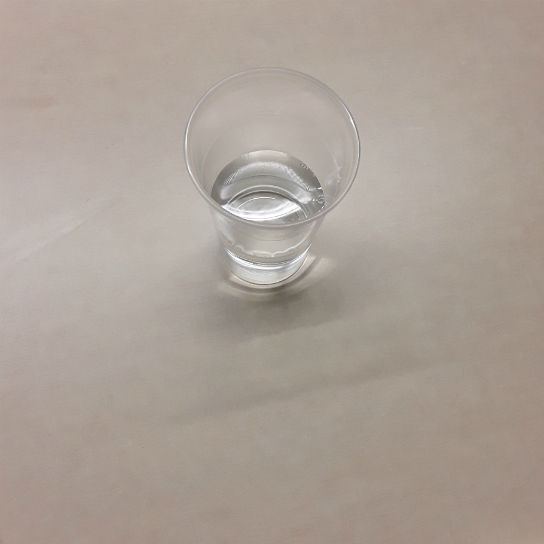

Supplement: Multimedia Appendix 2 [file formative_v4i12e15602_app2.zip › R Water container.jpg]

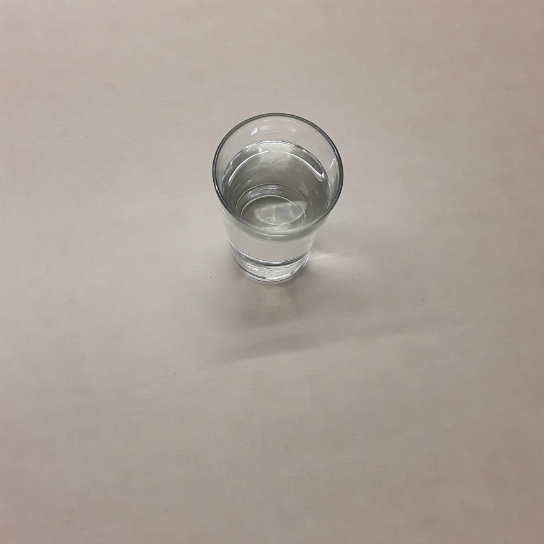

Supplement: Multimedia Appendix 2 [file formative_v4i12e15602_app2.zip › R Water ideal.jpg]

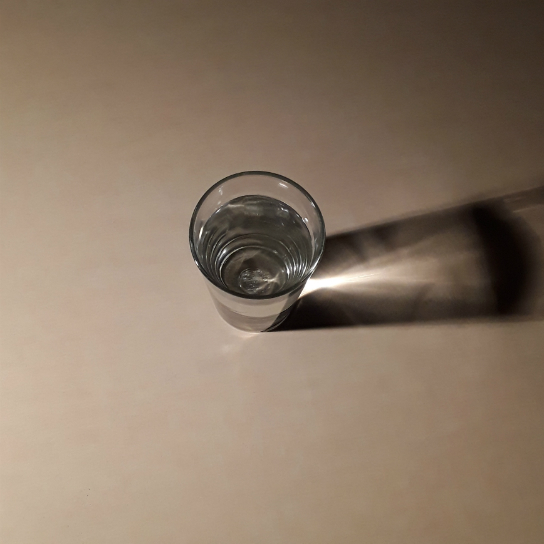

Supplement: Multimedia Appendix 2 [file formative_v4i12e15602_app2.zip › R Water light.jpg]

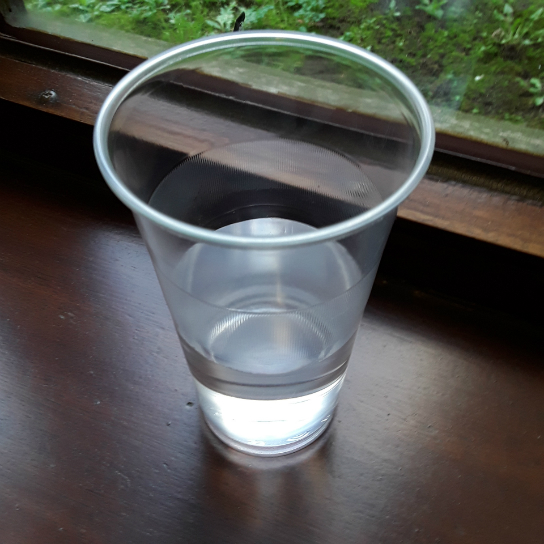

Supplement: Multimedia Appendix 2 [file formative_v4i12e15602_app2.zip › R Water real life.jpg]
