# Supplementary figures and images for: Use of Different Food Image Recognition Platforms in Dietary Assessment: Comparison Study (part 1 of 2)
Source: JMIR Form Res. 2020 Dec 7;4(12):e15602. doi: 10.2196/15602 (PMC7752530; doi:10.2196/15602)

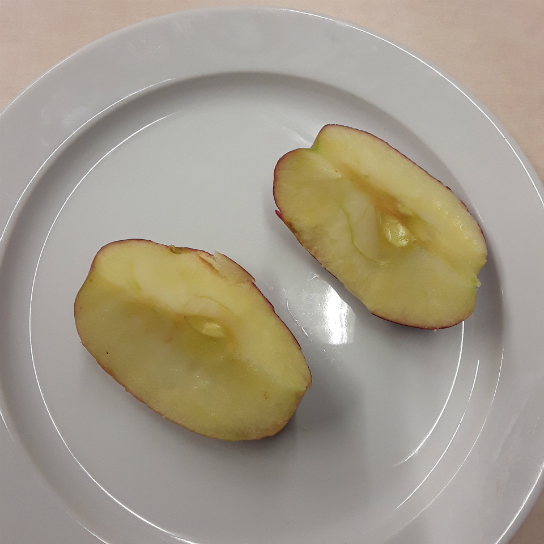

Supplement: Multimedia Appendix 2 [file formative_v4i12e15602_app2.zip › Apple angle.jpg]

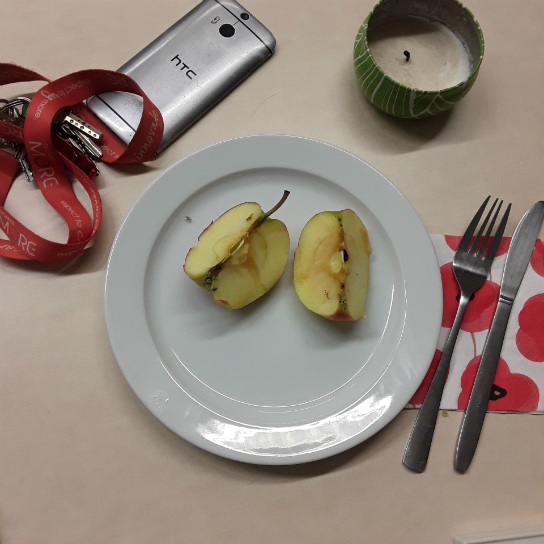

Supplement: Multimedia Appendix 2 [file formative_v4i12e15602_app2.zip › Apple clutter.jpg]

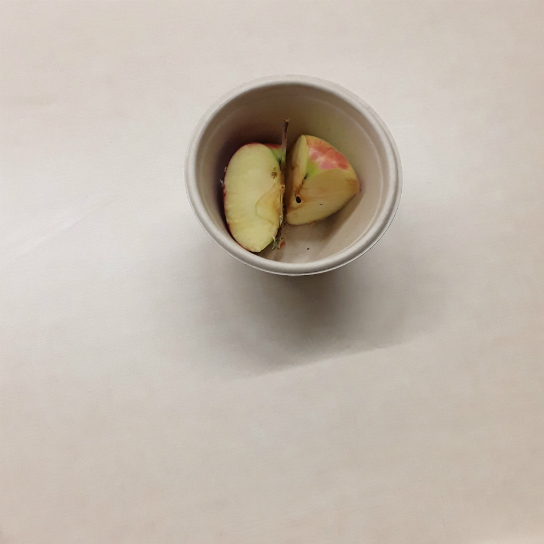

Supplement: Multimedia Appendix 2 [file formative_v4i12e15602_app2.zip › Apple container.jpg]

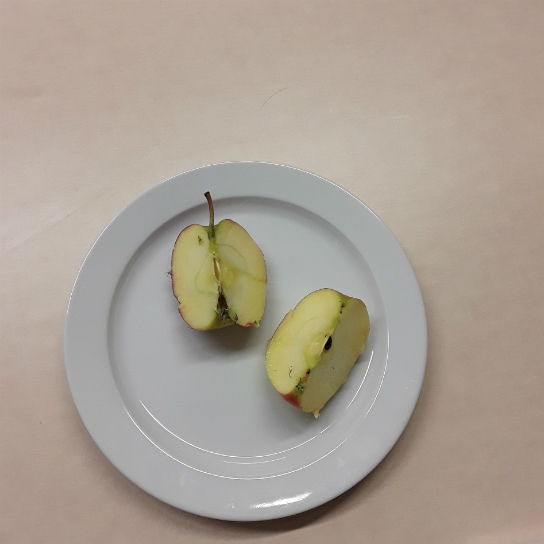

Supplement: Multimedia Appendix 2 [file formative_v4i12e15602_app2.zip › R Apple ideal.jpg]

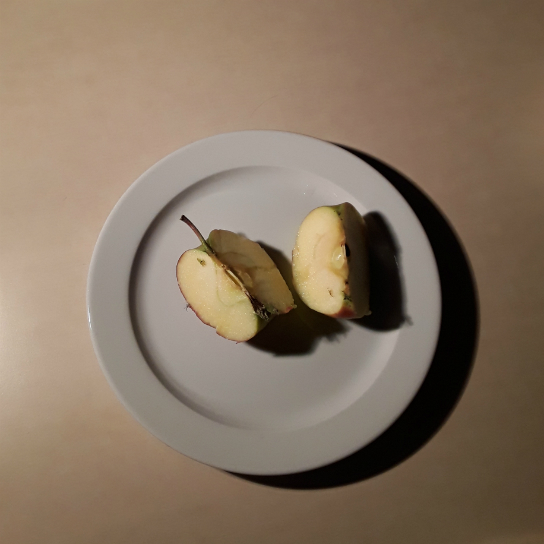

Supplement: Multimedia Appendix 2 [file formative_v4i12e15602_app2.zip › R Apple light.jpg]

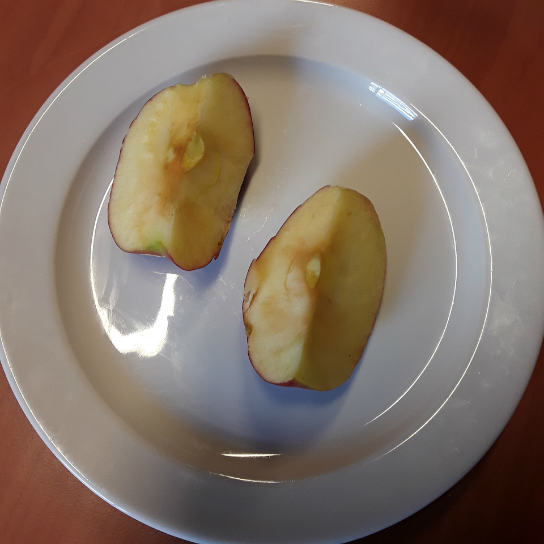

Supplement: Multimedia Appendix 2 [file formative_v4i12e15602_app2.zip › R Apple real life.jpg]

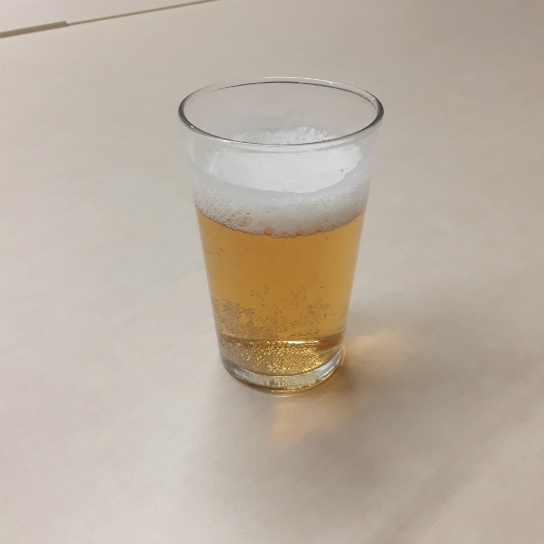

Supplement: Multimedia Appendix 2 [file formative_v4i12e15602_app2.zip › R Beer angle.jpg]

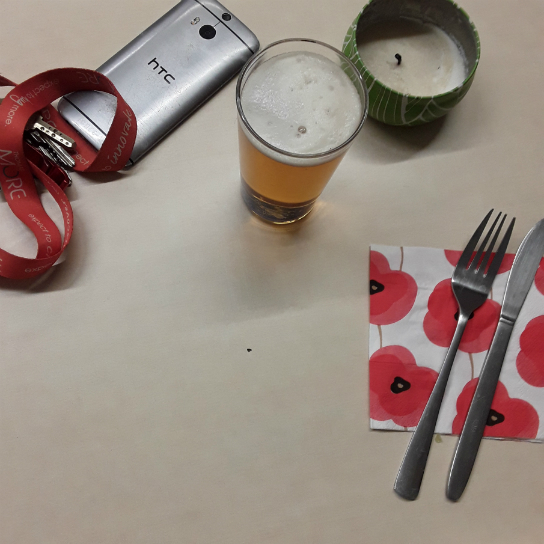

Supplement: Multimedia Appendix 2 [file formative_v4i12e15602_app2.zip › R Beer clutter.jpg]

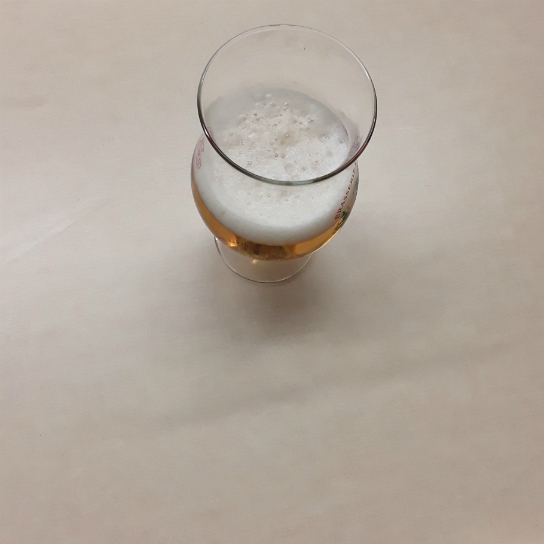

Supplement: Multimedia Appendix 2 [file formative_v4i12e15602_app2.zip › R Beer container.jpg]

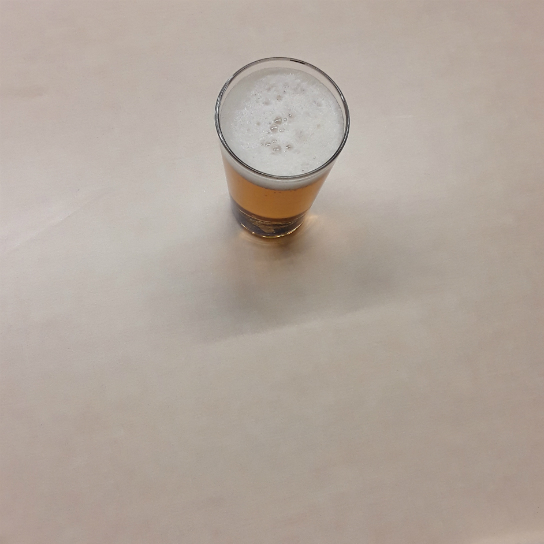

Supplement: Multimedia Appendix 2 [file formative_v4i12e15602_app2.zip › R Beer ideal.jpg]

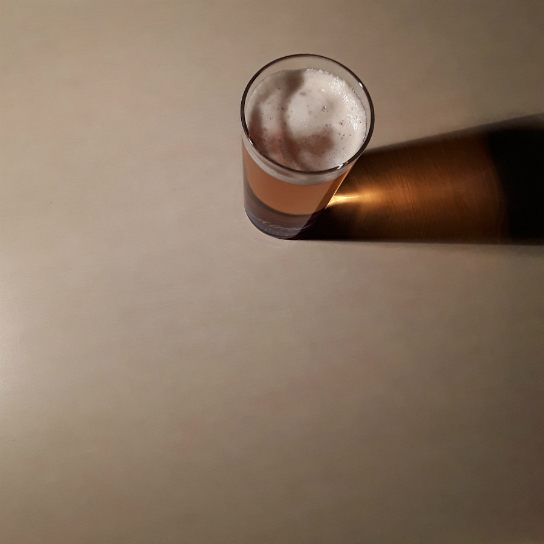

Supplement: Multimedia Appendix 2 [file formative_v4i12e15602_app2.zip › R Beer light.jpg]

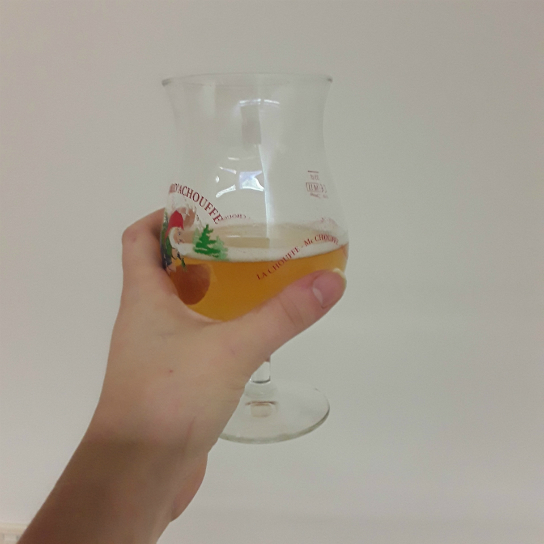

Supplement: Multimedia Appendix 2 [file formative_v4i12e15602_app2.zip › R Beer real life.jpg]

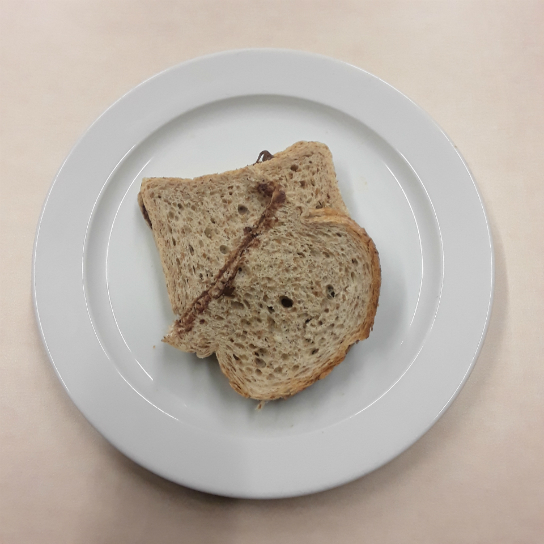

Supplement: Multimedia Appendix 2 [file formative_v4i12e15602_app2.zip › R Bread angle.jpg]

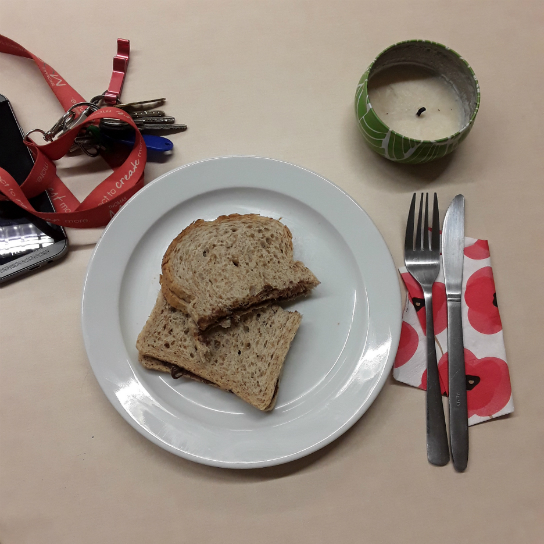

Supplement: Multimedia Appendix 2 [file formative_v4i12e15602_app2.zip › R Bread clutter.jpg]

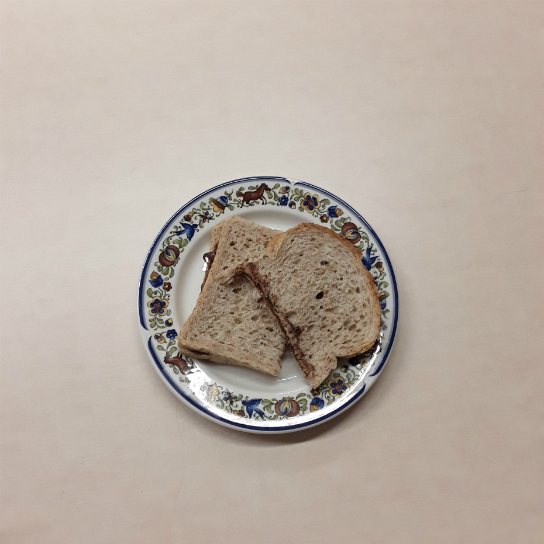

Supplement: Multimedia Appendix 2 [file formative_v4i12e15602_app2.zip › R Bread container.jpg]

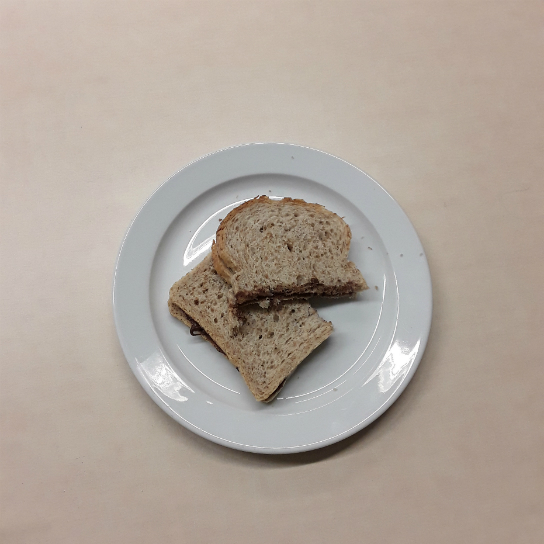

Supplement: Multimedia Appendix 2 [file formative_v4i12e15602_app2.zip › R Bread ideal.jpg]

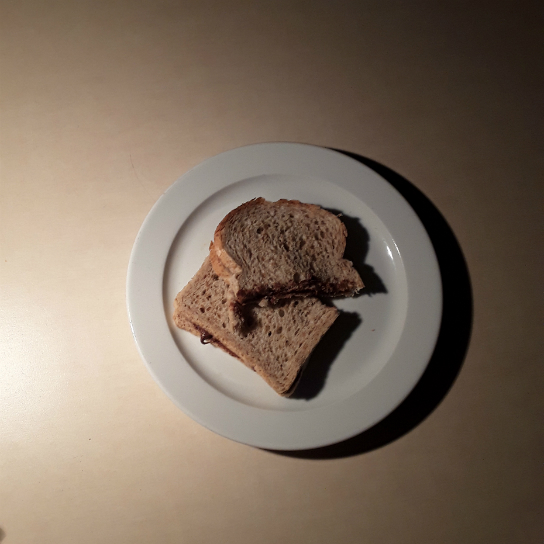

Supplement: Multimedia Appendix 2 [file formative_v4i12e15602_app2.zip › R Bread light.jpg]

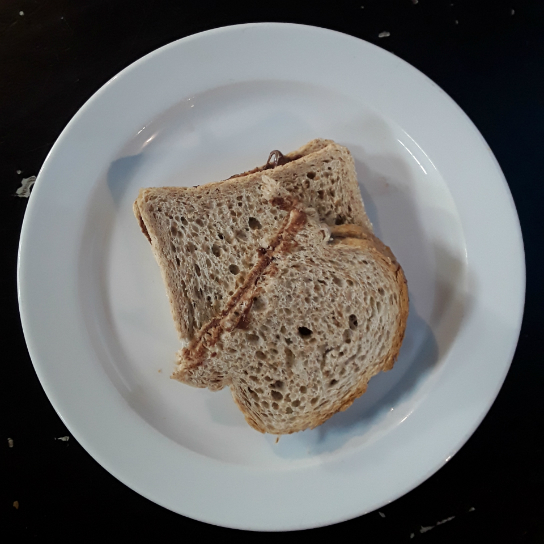

Supplement: Multimedia Appendix 2 [file formative_v4i12e15602_app2.zip › R Bread real life.jpg]

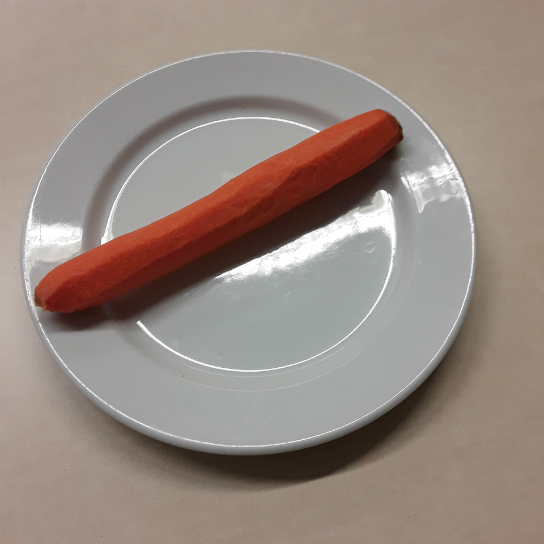

Supplement: Multimedia Appendix 2 [file formative_v4i12e15602_app2.zip › R Carrot angle.jpg]

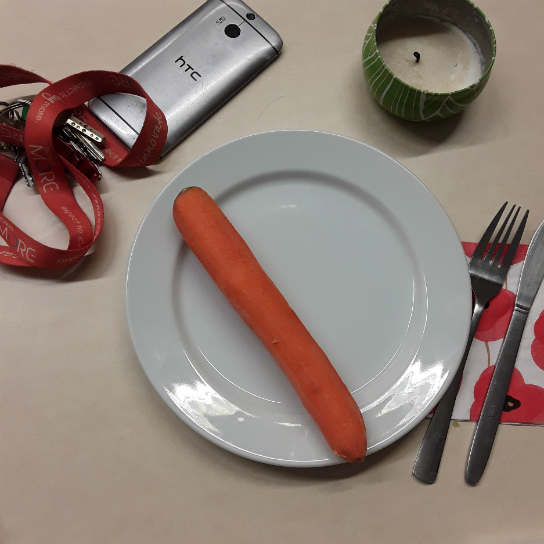

Supplement: Multimedia Appendix 2 [file formative_v4i12e15602_app2.zip › R Carrot clutter.jpg]

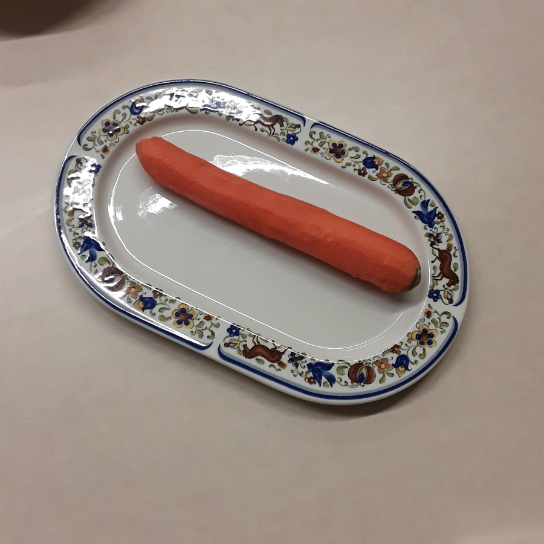

Supplement: Multimedia Appendix 2 [file formative_v4i12e15602_app2.zip › R Carrot container.jpg]

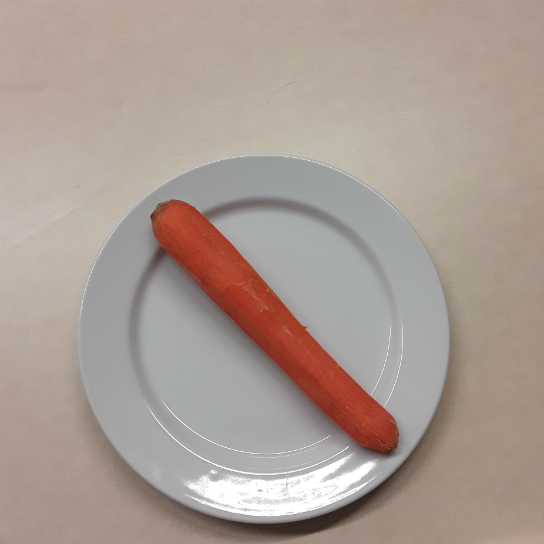

Supplement: Multimedia Appendix 2 [file formative_v4i12e15602_app2.zip › R Carrot ideal.jpg]

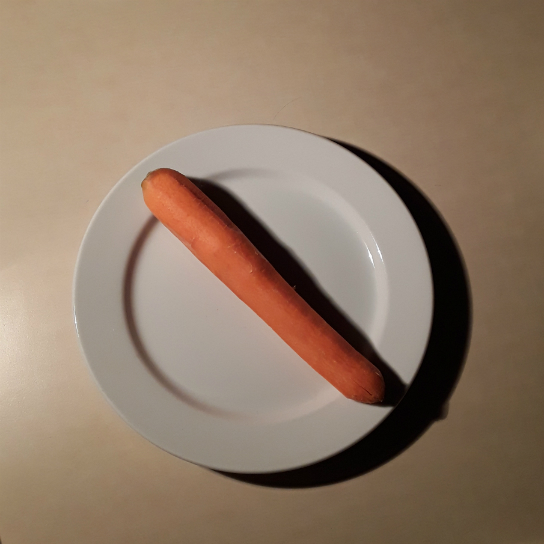

Supplement: Multimedia Appendix 2 [file formative_v4i12e15602_app2.zip › R Carrot light.jpg]

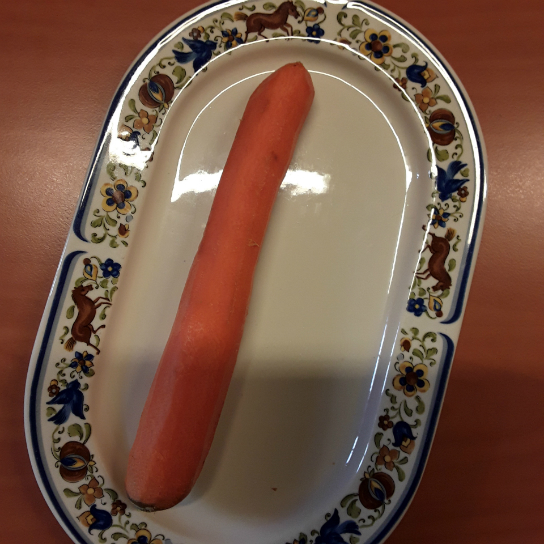

Supplement: Multimedia Appendix 2 [file formative_v4i12e15602_app2.zip › R Carrot real life.jpg]

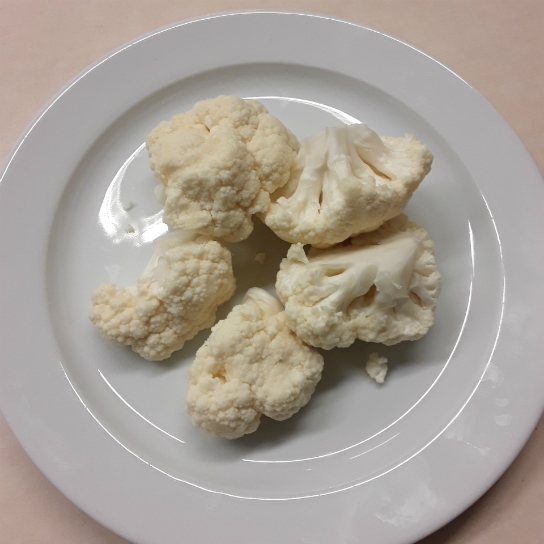

Supplement: Multimedia Appendix 2 [file formative_v4i12e15602_app2.zip › R Cauliflower angle.jpg]

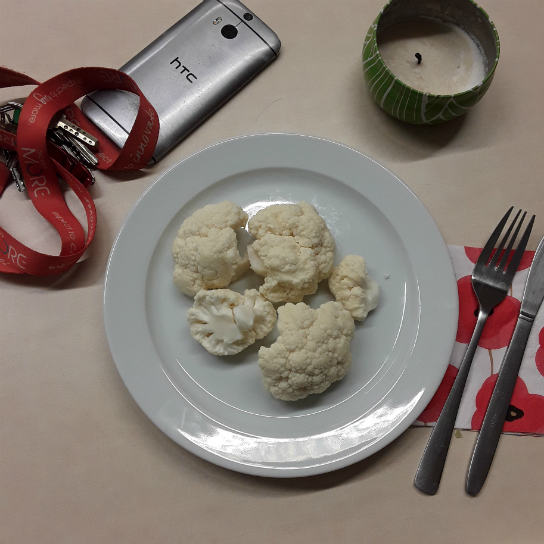

Supplement: Multimedia Appendix 2 [file formative_v4i12e15602_app2.zip › R Cauliflower clutter.jpg]

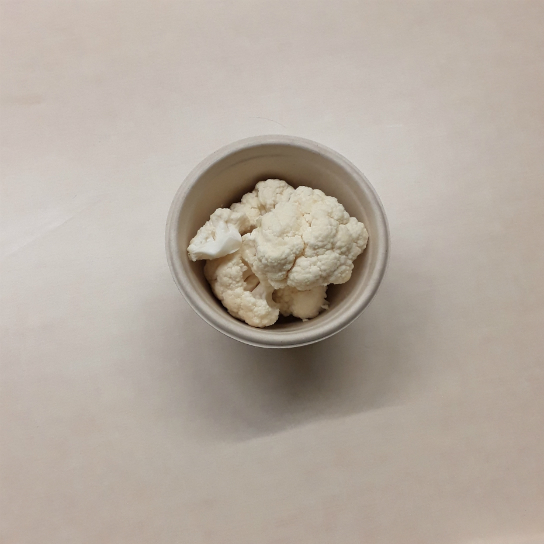

Supplement: Multimedia Appendix 2 [file formative_v4i12e15602_app2.zip › R Cauliflower container.jpg]

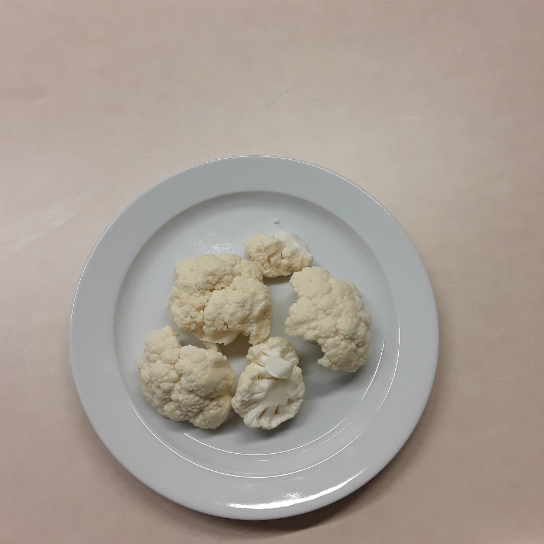

Supplement: Multimedia Appendix 2 [file formative_v4i12e15602_app2.zip › R Cauliflower ideal.jpg]

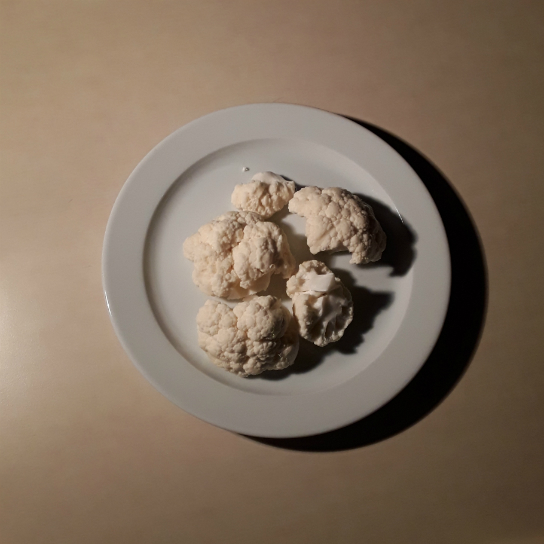

Supplement: Multimedia Appendix 2 [file formative_v4i12e15602_app2.zip › R Cauliflower light.jpg]

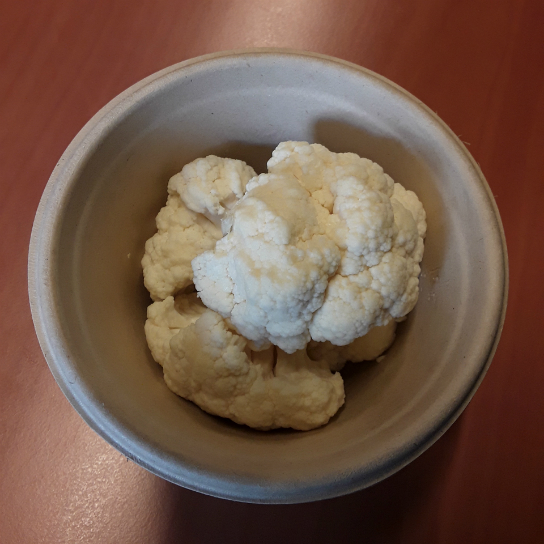

Supplement: Multimedia Appendix 2 [file formative_v4i12e15602_app2.zip › R Cauliflower real life.jpg]

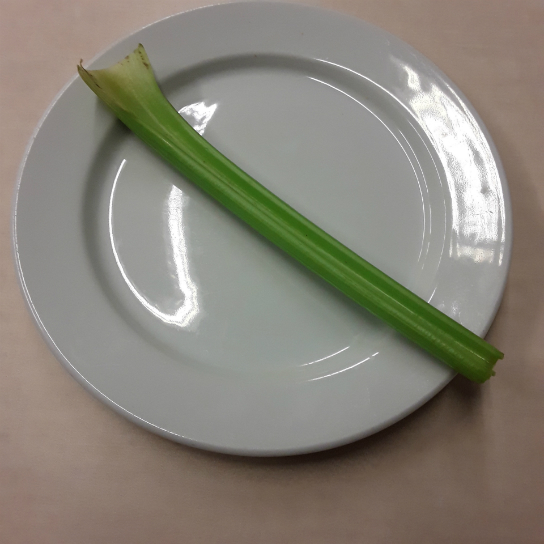

Supplement: Multimedia Appendix 2 [file formative_v4i12e15602_app2.zip › R Celery angle.jpg]

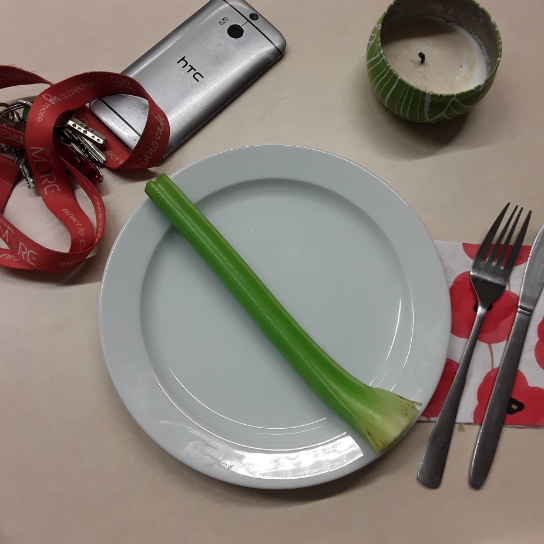

Supplement: Multimedia Appendix 2 [file formative_v4i12e15602_app2.zip › R Celery clutter.jpg]

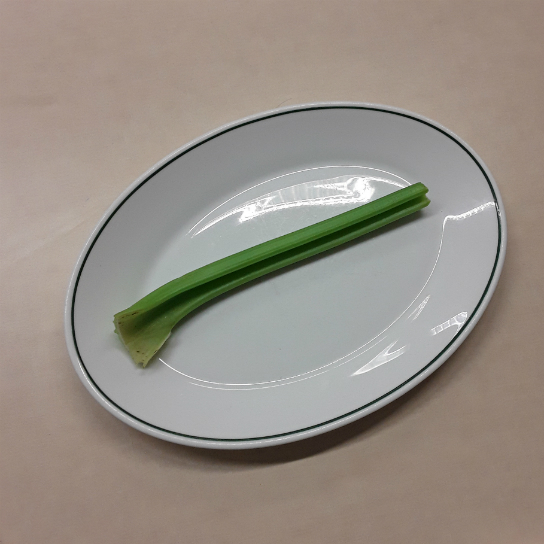

Supplement: Multimedia Appendix 2 [file formative_v4i12e15602_app2.zip › R Celery container.jpg]

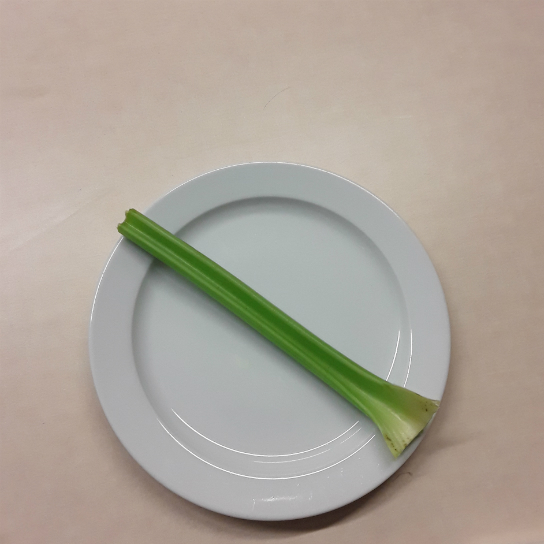

Supplement: Multimedia Appendix 2 [file formative_v4i12e15602_app2.zip › R Celery ideal.jpg]

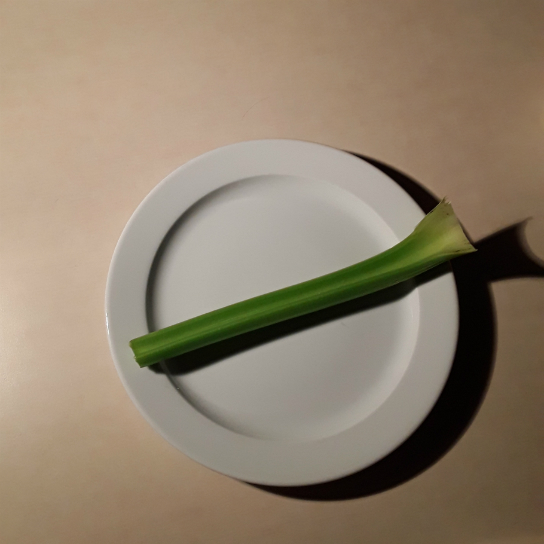

Supplement: Multimedia Appendix 2 [file formative_v4i12e15602_app2.zip › R Celery light.jpg]

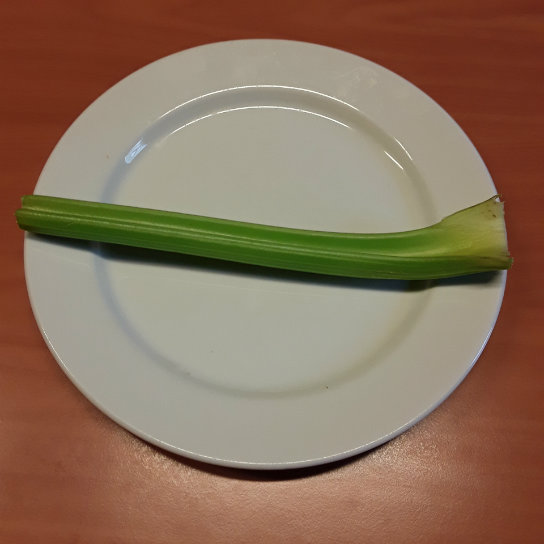

Supplement: Multimedia Appendix 2 [file formative_v4i12e15602_app2.zip › R Celery real life.jpg]

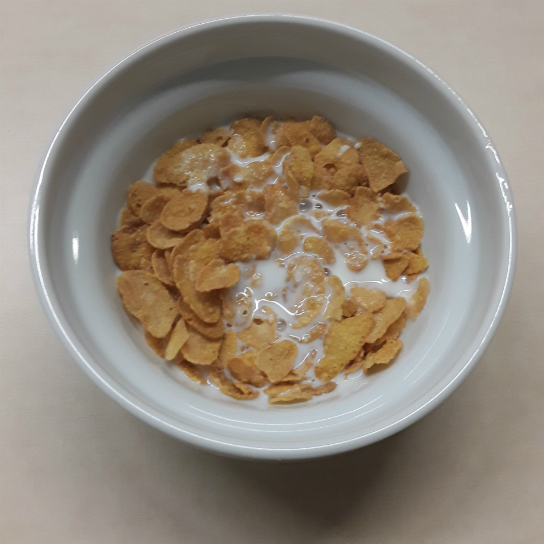

Supplement: Multimedia Appendix 2 [file formative_v4i12e15602_app2.zip › R Cereal angle.jpg]

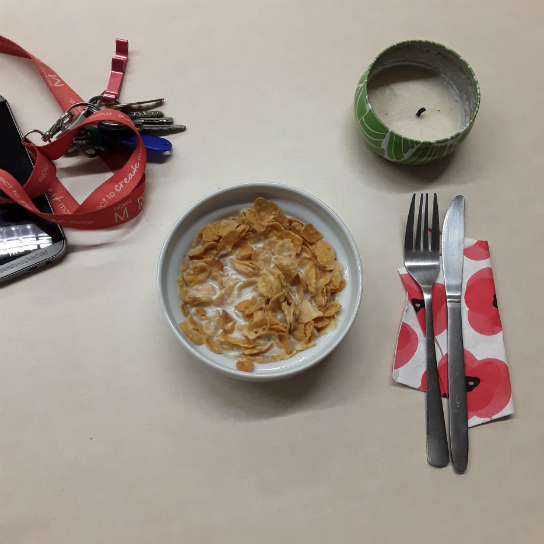

Supplement: Multimedia Appendix 2 [file formative_v4i12e15602_app2.zip › R Cereal clutter.jpg]

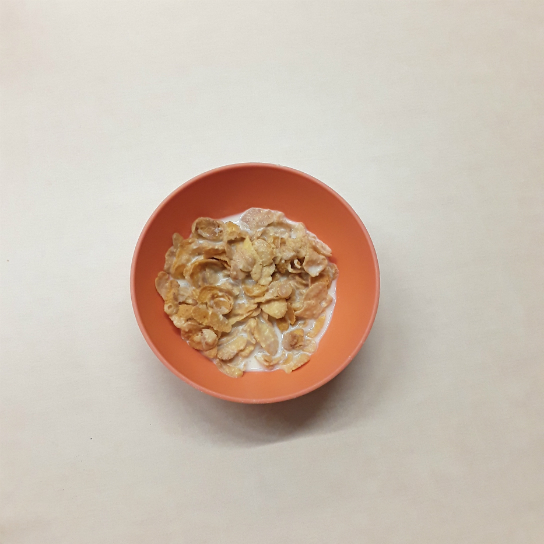

Supplement: Multimedia Appendix 2 [file formative_v4i12e15602_app2.zip › R Cereal container.jpg]

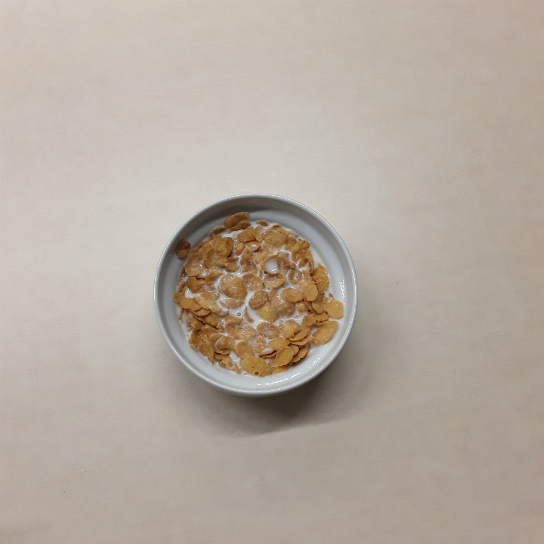

Supplement: Multimedia Appendix 2 [file formative_v4i12e15602_app2.zip › R Cereal ideal.jpg]

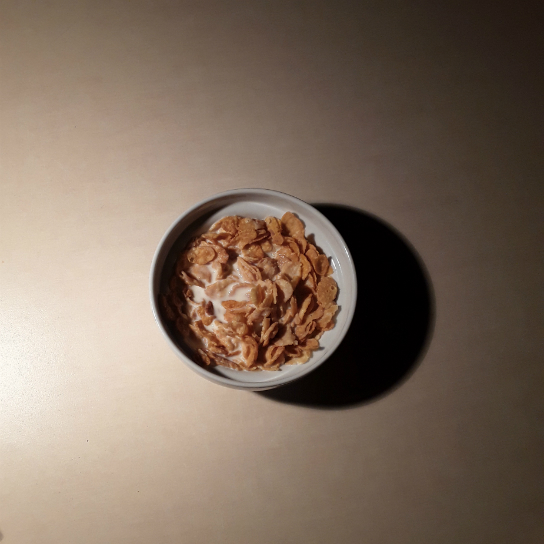

Supplement: Multimedia Appendix 2 [file formative_v4i12e15602_app2.zip › R Cereal light.jpg]

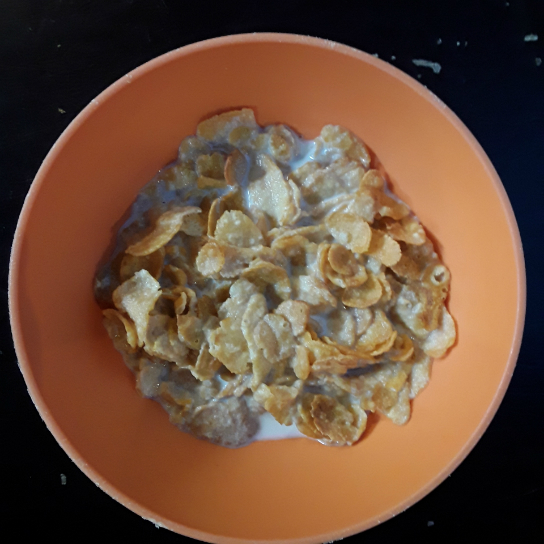

Supplement: Multimedia Appendix 2 [file formative_v4i12e15602_app2.zip › R Cereal real life.jpg]

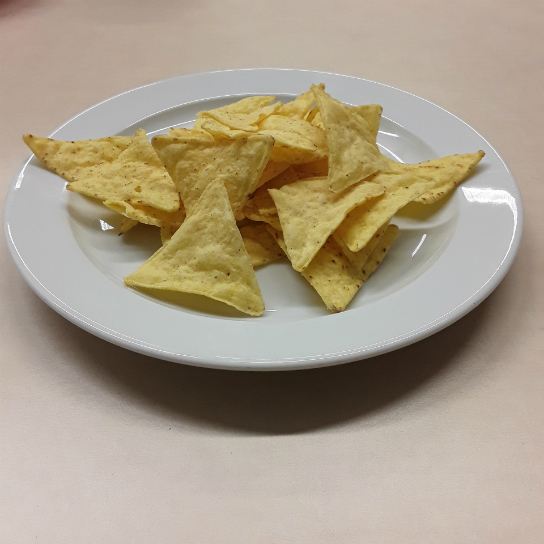

Supplement: Multimedia Appendix 2 [file formative_v4i12e15602_app2.zip › R Chips angle.jpg]

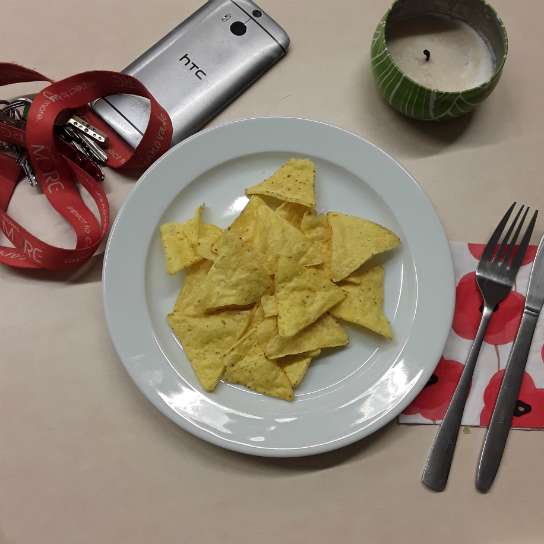

Supplement: Multimedia Appendix 2 [file formative_v4i12e15602_app2.zip › R Chips clutter.jpg]

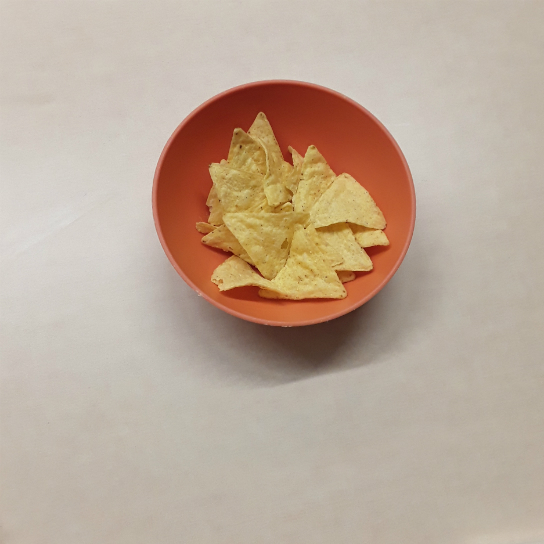

Supplement: Multimedia Appendix 2 [file formative_v4i12e15602_app2.zip › R Chips container.jpg]

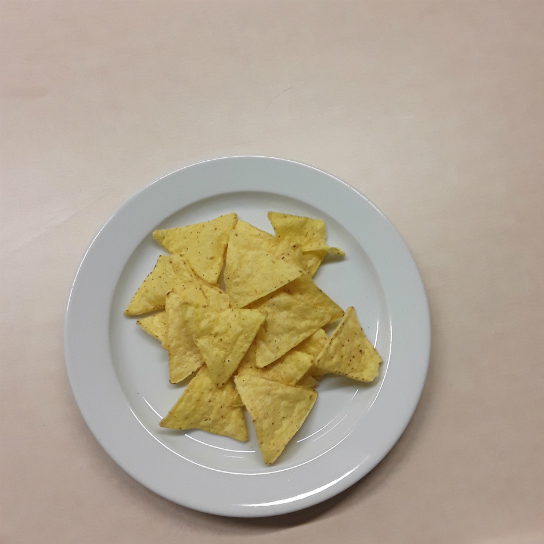

Supplement: Multimedia Appendix 2 [file formative_v4i12e15602_app2.zip › R Chips ideal.jpg]

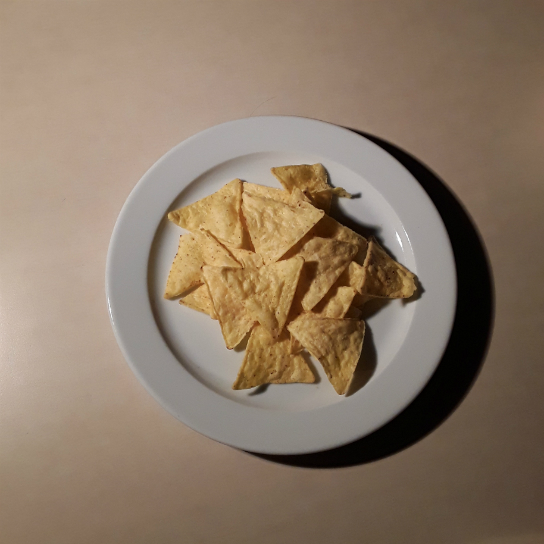

Supplement: Multimedia Appendix 2 [file formative_v4i12e15602_app2.zip › R Chips light.jpg]

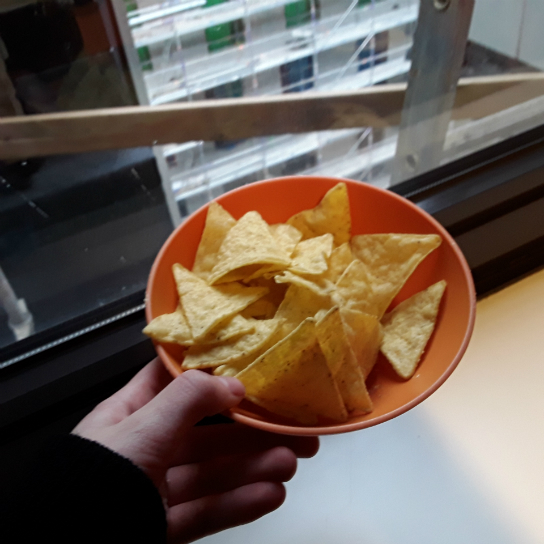

Supplement: Multimedia Appendix 2 [file formative_v4i12e15602_app2.zip › R Chips real life.jpg]

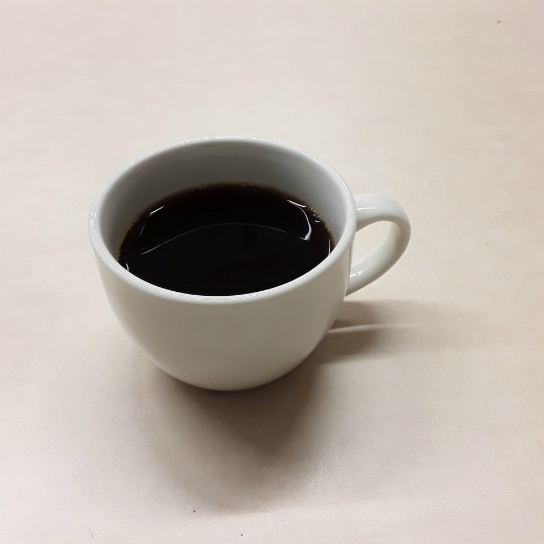

Supplement: Multimedia Appendix 2 [file formative_v4i12e15602_app2.zip › R Coffee angle.jpg]

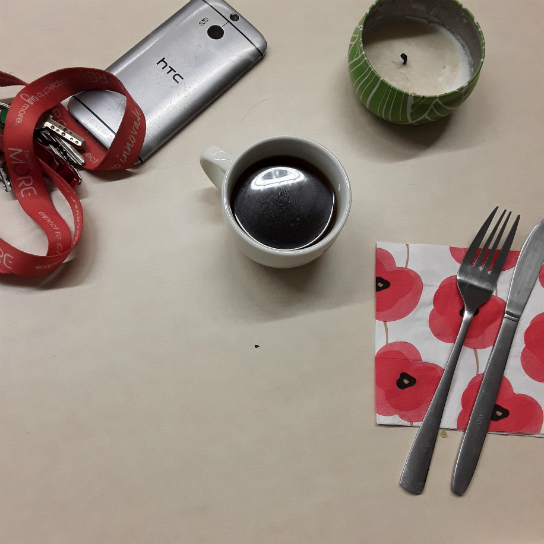

Supplement: Multimedia Appendix 2 [file formative_v4i12e15602_app2.zip › R Coffee clutter.jpg]

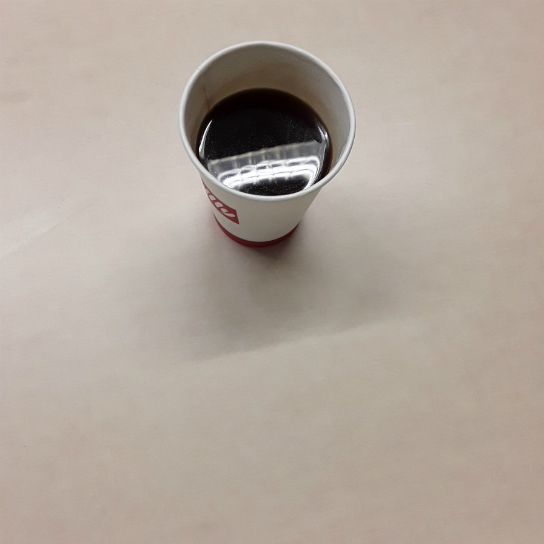

Supplement: Multimedia Appendix 2 [file formative_v4i12e15602_app2.zip › R Coffee container.jpg]

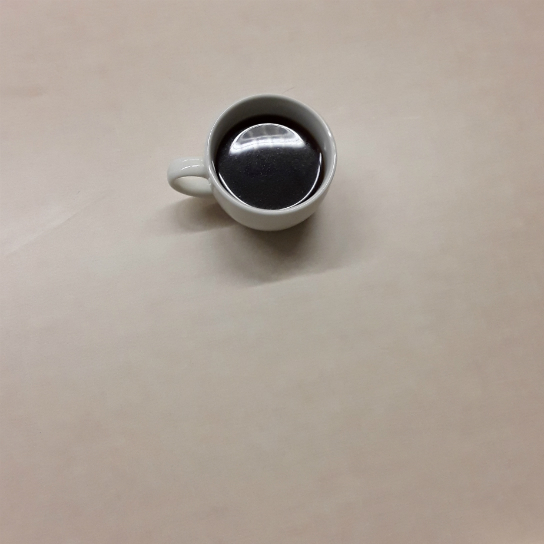

Supplement: Multimedia Appendix 2 [file formative_v4i12e15602_app2.zip › R Coffee ideal.jpg]

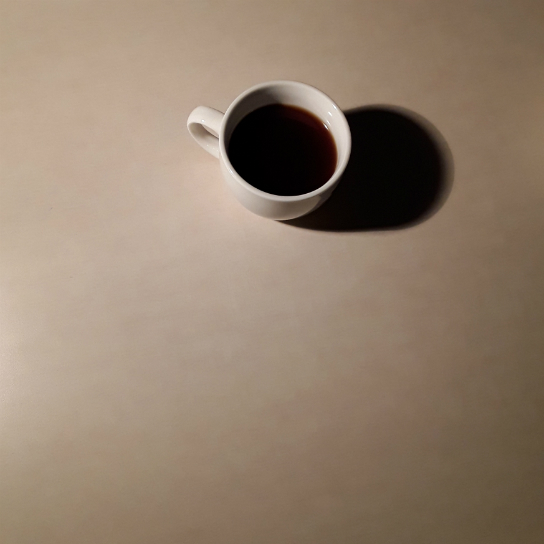

Supplement: Multimedia Appendix 2 [file formative_v4i12e15602_app2.zip › R Coffee light.jpg]

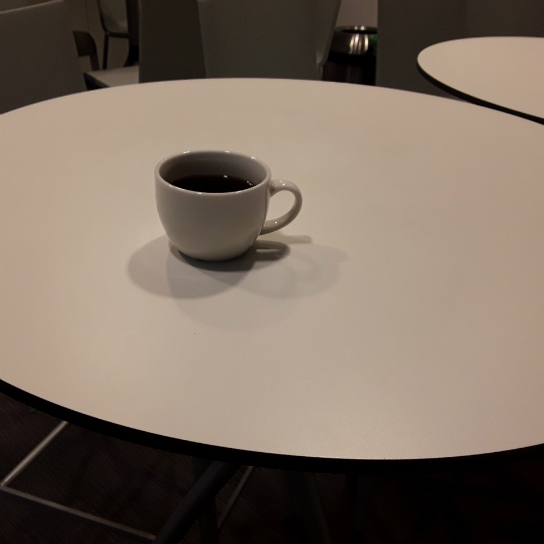

Supplement: Multimedia Appendix 2 [file formative_v4i12e15602_app2.zip › R Coffee real life.jpg]

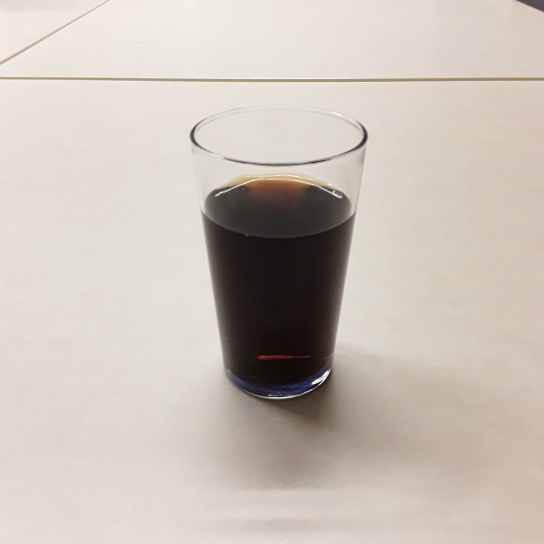

Supplement: Multimedia Appendix 2 [file formative_v4i12e15602_app2.zip › R Coke angle.jpg]

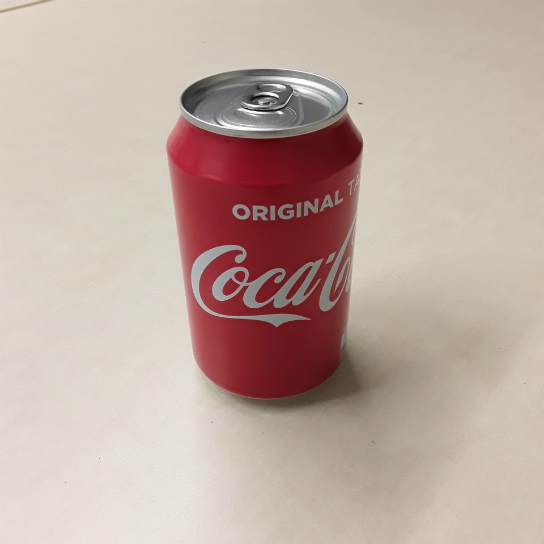

Supplement: Multimedia Appendix 2 [file formative_v4i12e15602_app2.zip › R Coke can angle.jpg]

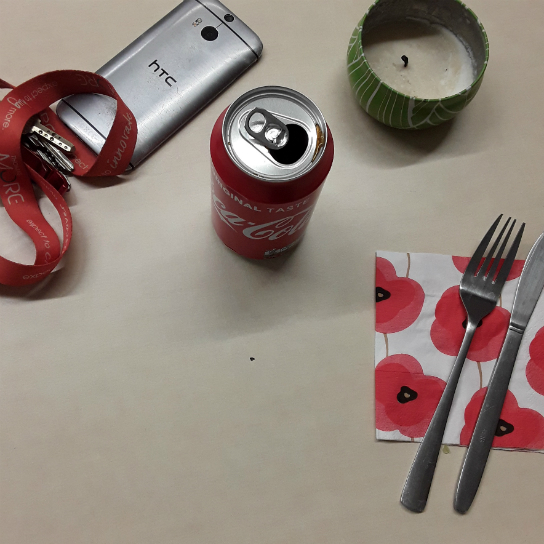

Supplement: Multimedia Appendix 2 [file formative_v4i12e15602_app2.zip › R Coke can clutter.jpg]

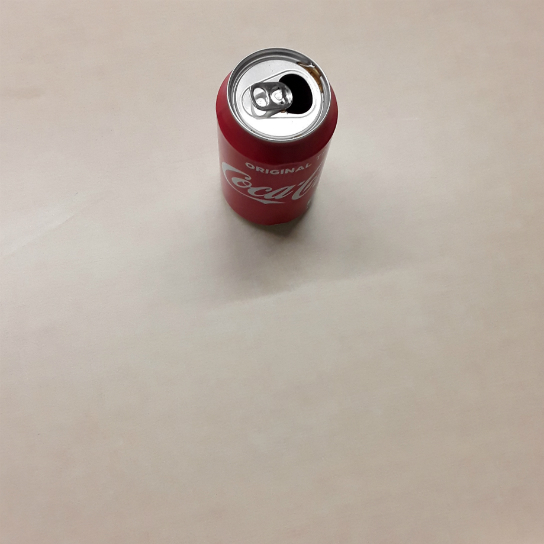

Supplement: Multimedia Appendix 2 [file formative_v4i12e15602_app2.zip › R Coke can ideal.jpg]

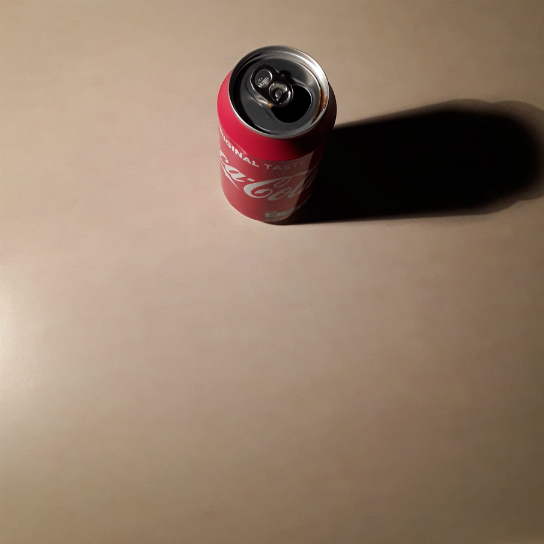

Supplement: Multimedia Appendix 2 [file formative_v4i12e15602_app2.zip › R Coke can light.jpg]

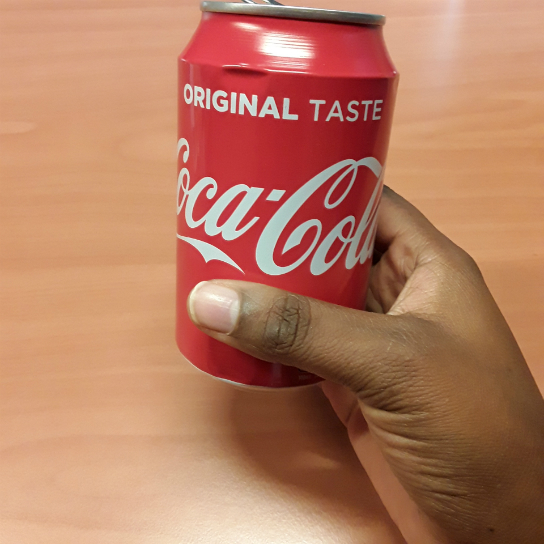

Supplement: Multimedia Appendix 2 [file formative_v4i12e15602_app2.zip › R Coke can real life.jpg]

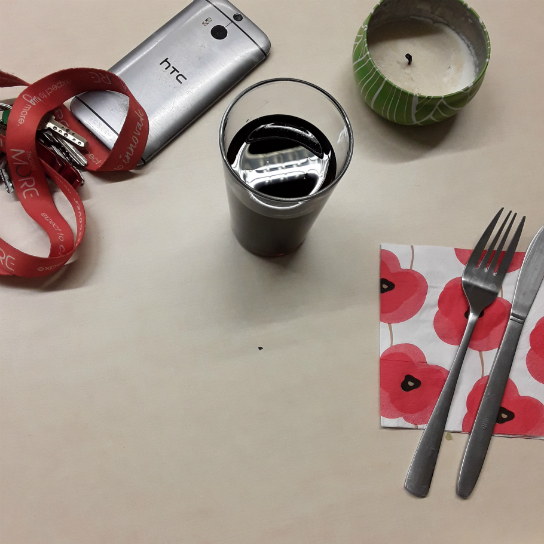

Supplement: Multimedia Appendix 2 [file formative_v4i12e15602_app2.zip › R Coke clutter.jpg]

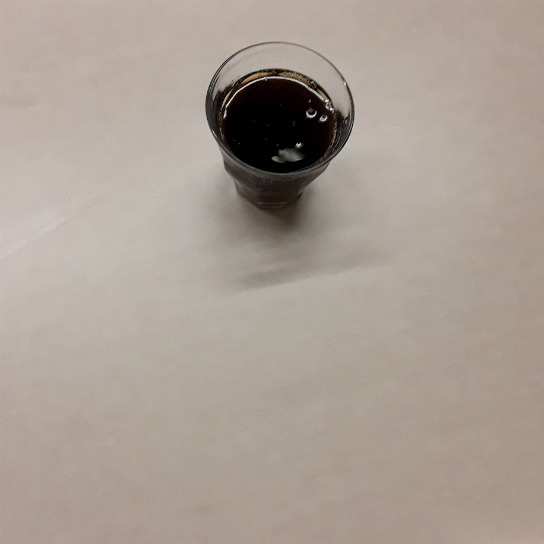

Supplement: Multimedia Appendix 2 [file formative_v4i12e15602_app2.zip › R Coke container.jpg]

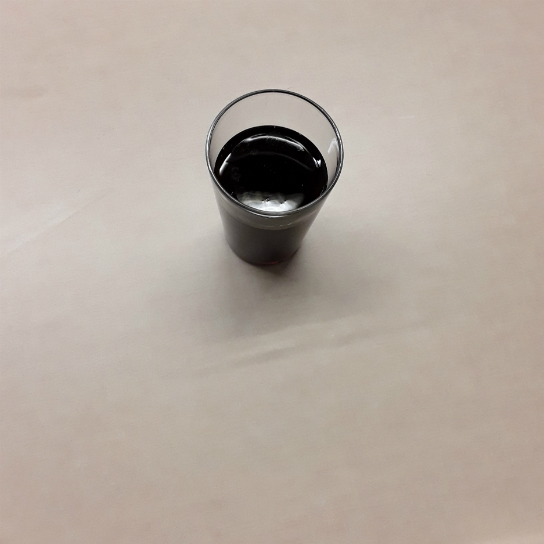

Supplement: Multimedia Appendix 2 [file formative_v4i12e15602_app2.zip › R Coke ideal.jpg]

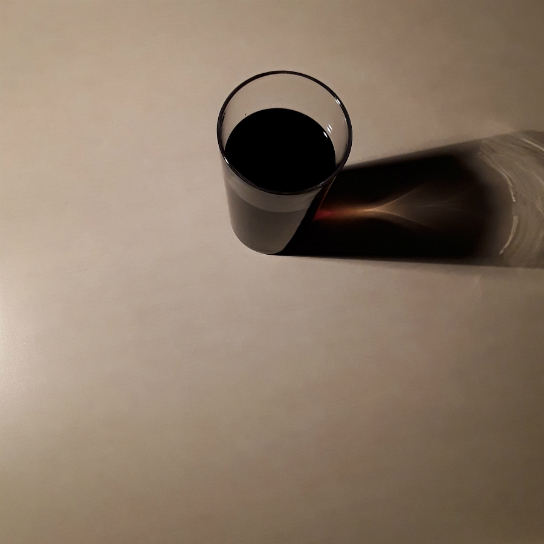

Supplement: Multimedia Appendix 2 [file formative_v4i12e15602_app2.zip › R Coke light.jpg]

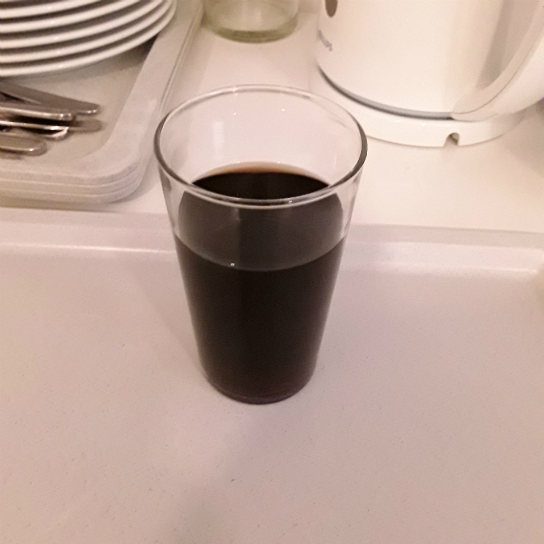

Supplement: Multimedia Appendix 2 [file formative_v4i12e15602_app2.zip › R Coke real life.jpg]

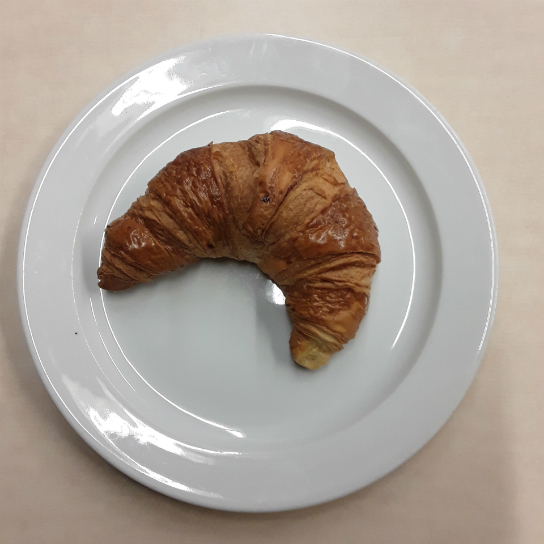

Supplement: Multimedia Appendix 2 [file formative_v4i12e15602_app2.zip › R Croissant angle.jpg]

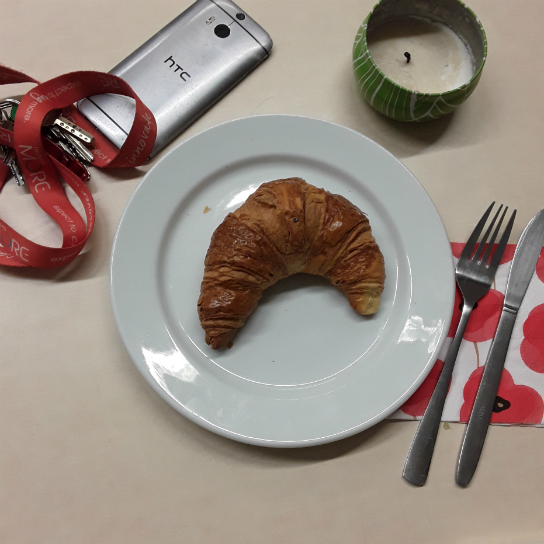

Supplement: Multimedia Appendix 2 [file formative_v4i12e15602_app2.zip › R Croissant clutter.jpg]

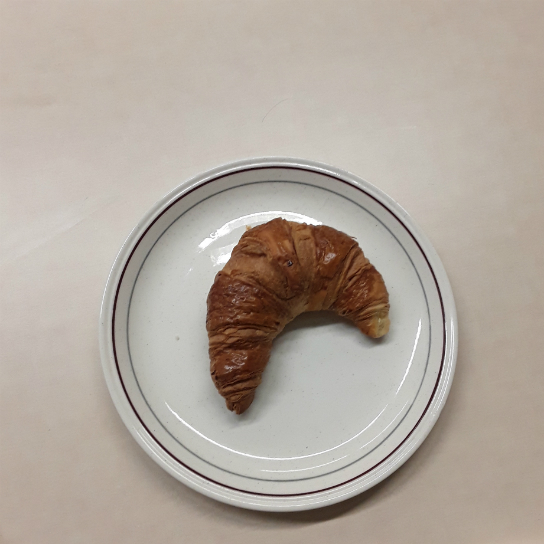

Supplement: Multimedia Appendix 2 [file formative_v4i12e15602_app2.zip › R Croissant container.jpg]

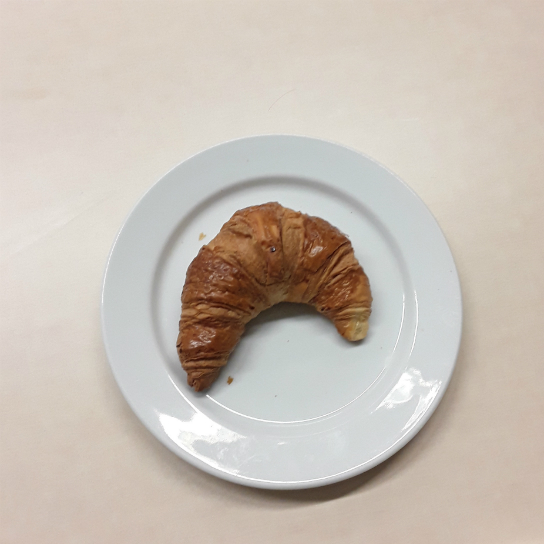

Supplement: Multimedia Appendix 2 [file formative_v4i12e15602_app2.zip › R Croissant ideal.jpg]

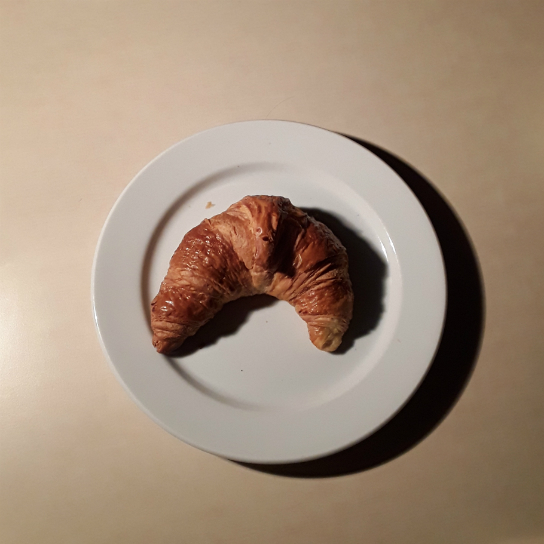

Supplement: Multimedia Appendix 2 [file formative_v4i12e15602_app2.zip › R Croissant light.jpg]

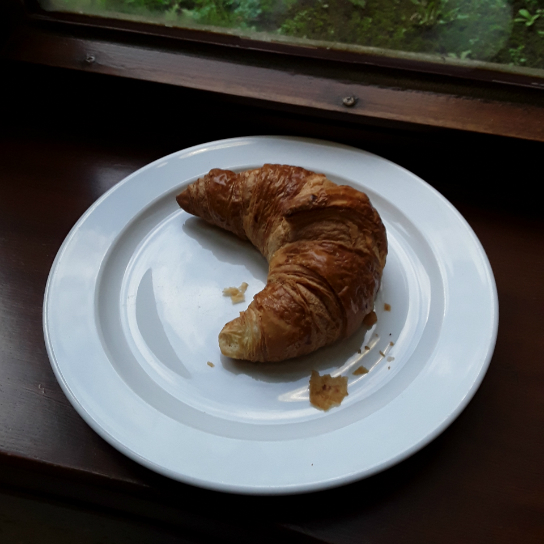

Supplement: Multimedia Appendix 2 [file formative_v4i12e15602_app2.zip › R Croissant real life.jpg]

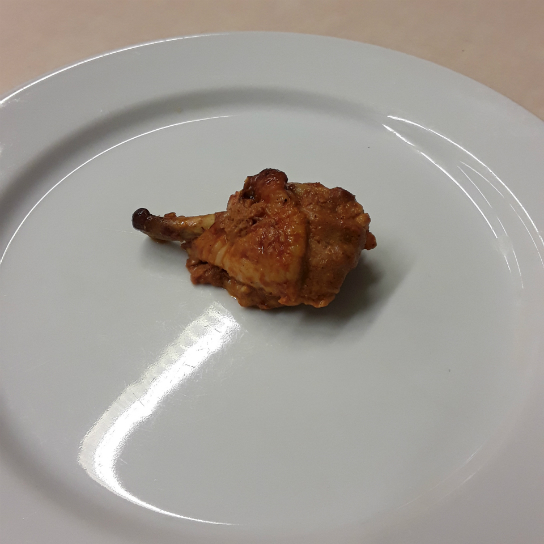

Supplement: Multimedia Appendix 2 [file formative_v4i12e15602_app2.zip › R Drumstick angle.jpg]

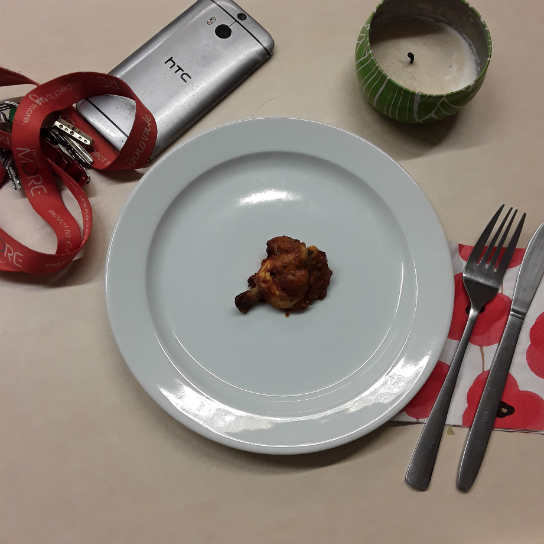

Supplement: Multimedia Appendix 2 [file formative_v4i12e15602_app2.zip › R Drumstick clutter.jpg]

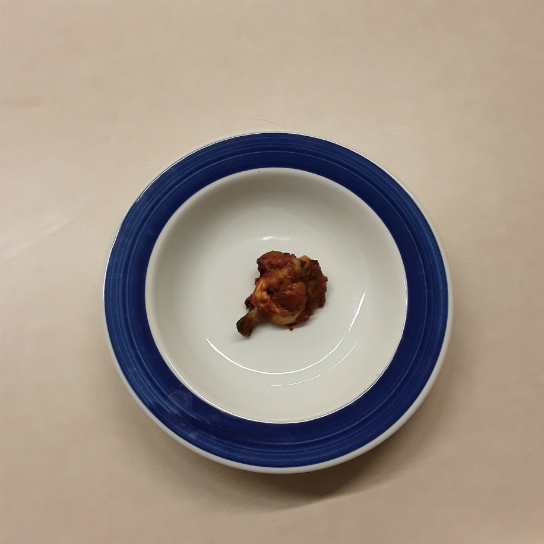

Supplement: Multimedia Appendix 2 [file formative_v4i12e15602_app2.zip › R Drumstick container.jpg]

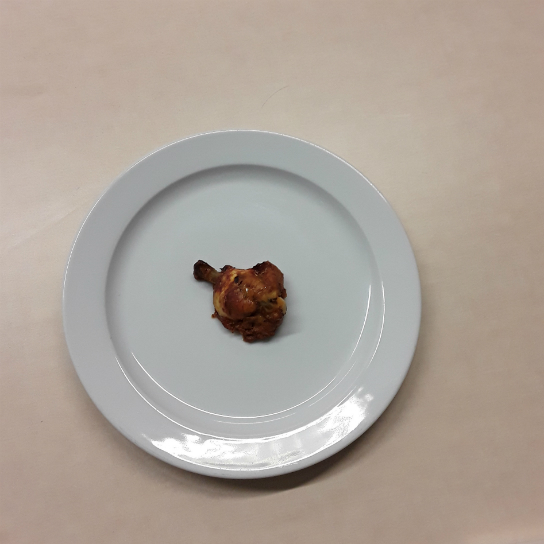

Supplement: Multimedia Appendix 2 [file formative_v4i12e15602_app2.zip › R Drumstick ideal.jpg]

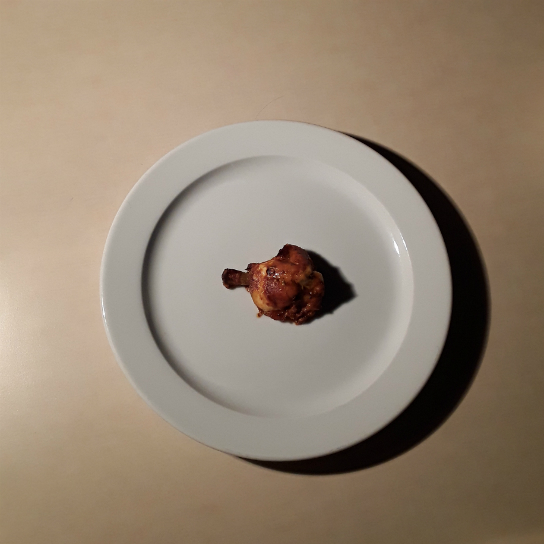

Supplement: Multimedia Appendix 2 [file formative_v4i12e15602_app2.zip › R Drumstick light.jpg]

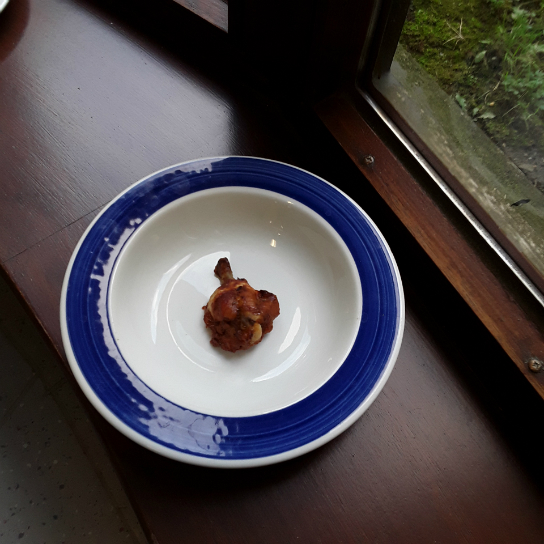

Supplement: Multimedia Appendix 2 [file formative_v4i12e15602_app2.zip › R Drumstick real life.jpg]

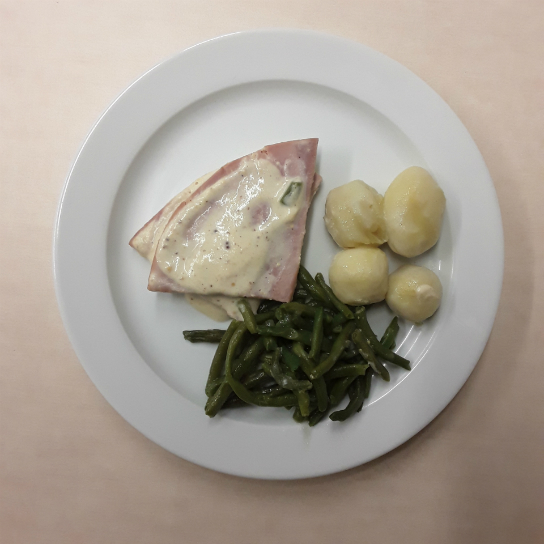

Supplement: Multimedia Appendix 2 [file formative_v4i12e15602_app2.zip › R Ham beans angle.jpg]

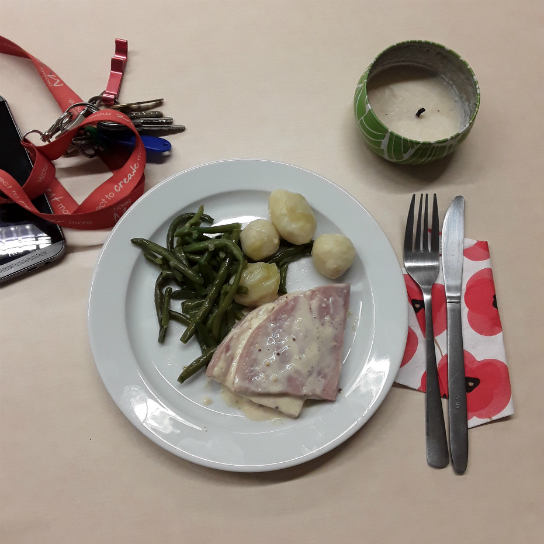

Supplement: Multimedia Appendix 2 [file formative_v4i12e15602_app2.zip › R Ham beans clutter.jpg]

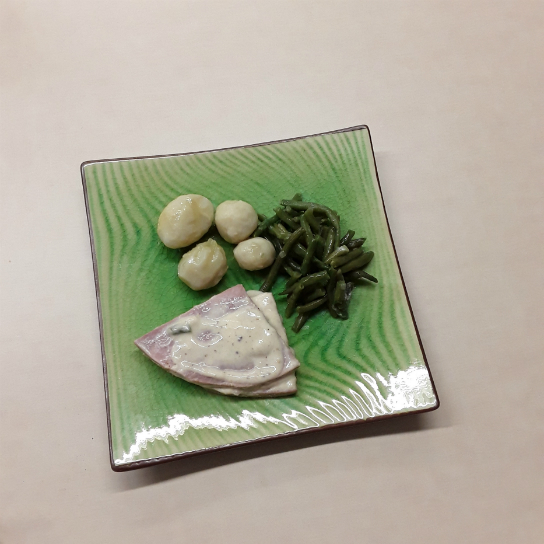

Supplement: Multimedia Appendix 2 [file formative_v4i12e15602_app2.zip › R Ham beans container.jpg]

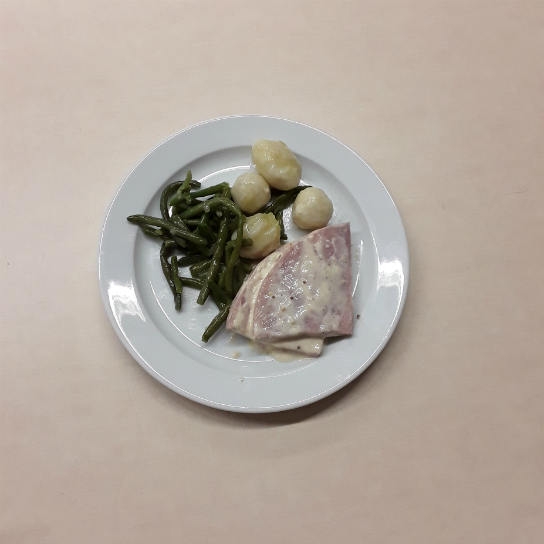

Supplement: Multimedia Appendix 2 [file formative_v4i12e15602_app2.zip › R Ham beans ideal.jpg]

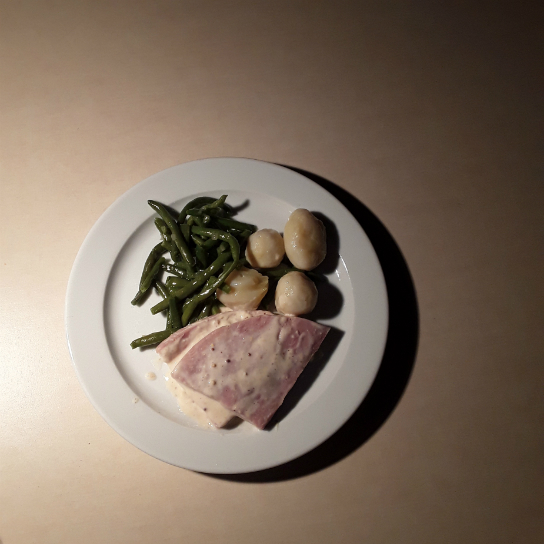

Supplement: Multimedia Appendix 2 [file formative_v4i12e15602_app2.zip › R Ham beans light.jpg]

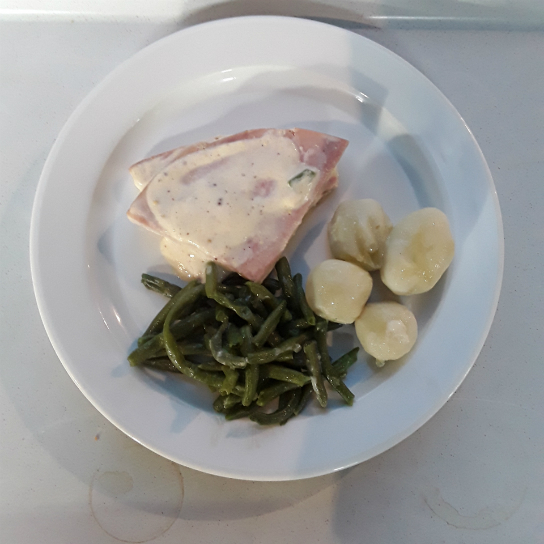

Supplement: Multimedia Appendix 2 [file formative_v4i12e15602_app2.zip › R Ham beans real life.jpg]

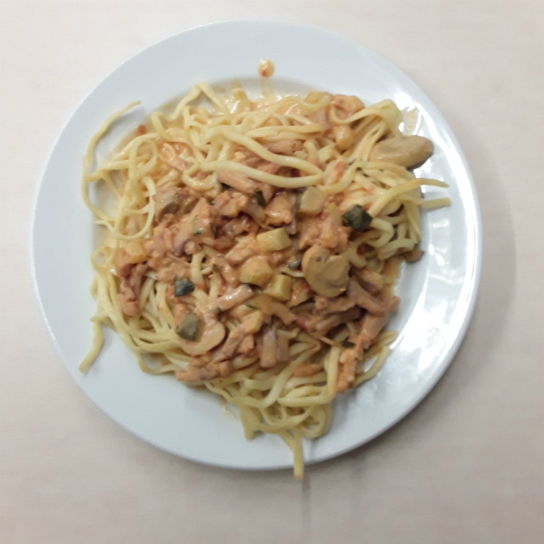

Supplement: Multimedia Appendix 2 [file formative_v4i12e15602_app2.zip › R Linguini angle.jpg]

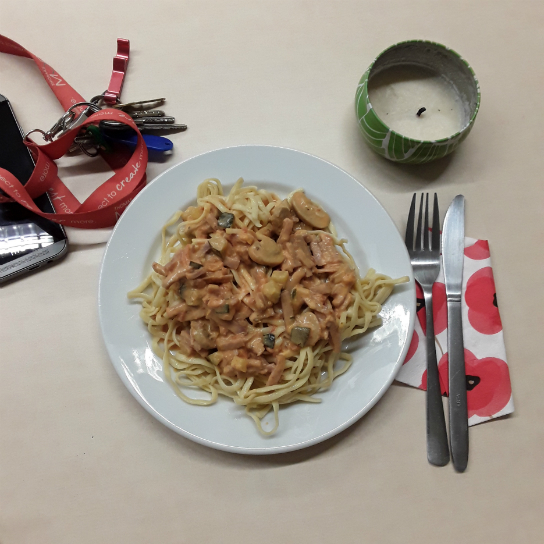

Supplement: Multimedia Appendix 2 [file formative_v4i12e15602_app2.zip › R Linguini clutter.jpg]

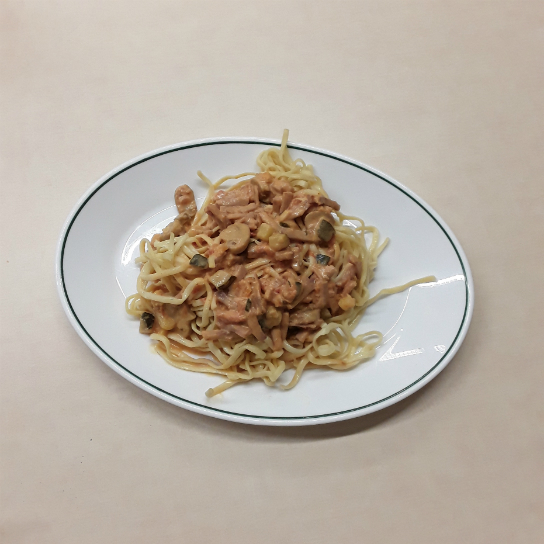

Supplement: Multimedia Appendix 2 [file formative_v4i12e15602_app2.zip › R Linguini container.jpg]

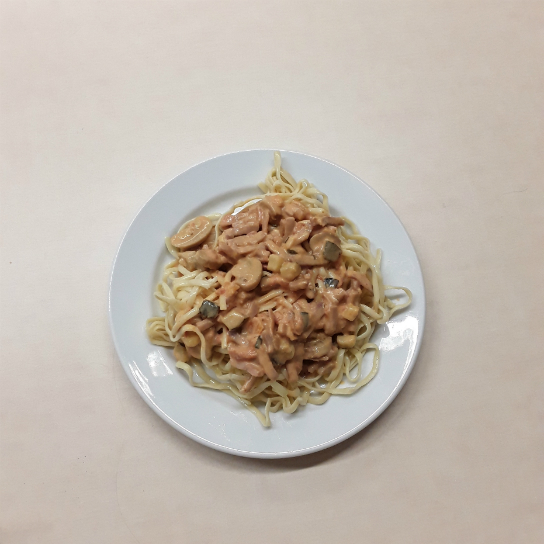

Supplement: Multimedia Appendix 2 [file formative_v4i12e15602_app2.zip › R Linguini ideal.jpg]

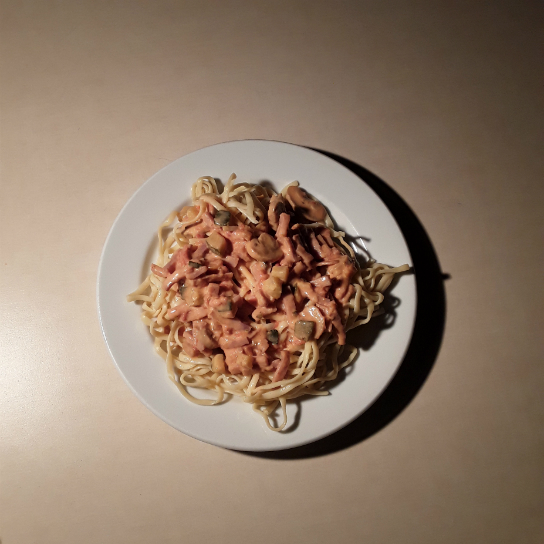

Supplement: Multimedia Appendix 2 [file formative_v4i12e15602_app2.zip › R Linguini light.jpg]

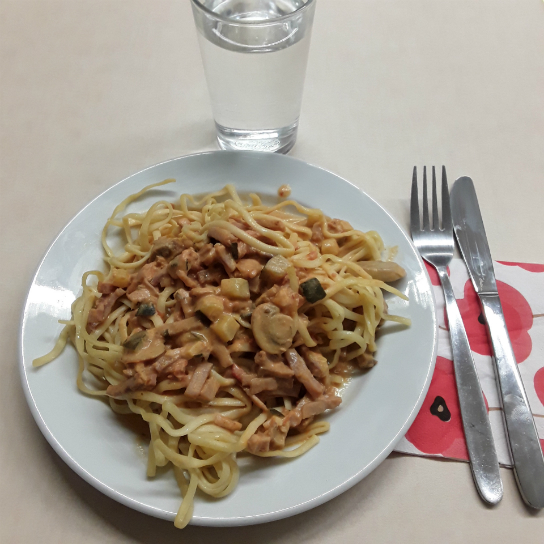

Supplement: Multimedia Appendix 2 [file formative_v4i12e15602_app2.zip › R Linguini real life.jpg]

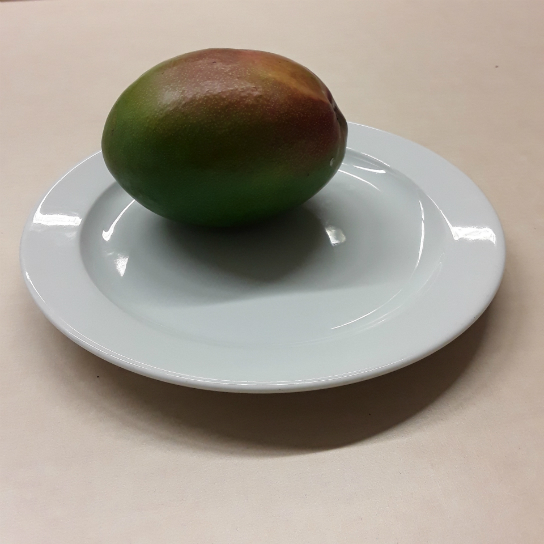

Supplement: Multimedia Appendix 2 [file formative_v4i12e15602_app2.zip › R Mango angle.jpg]

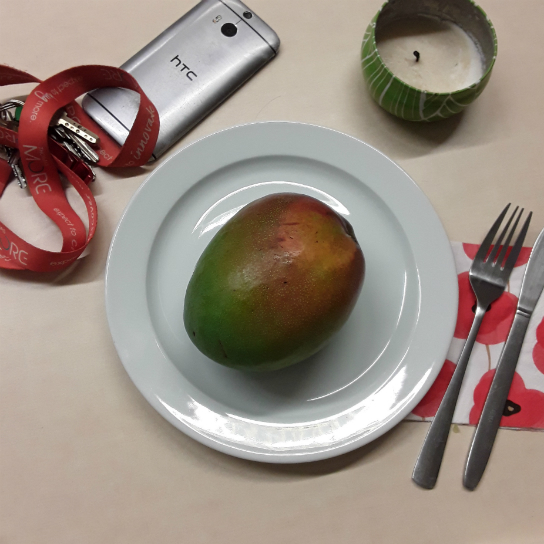

Supplement: Multimedia Appendix 2 [file formative_v4i12e15602_app2.zip › R Mango clutter.jpg]

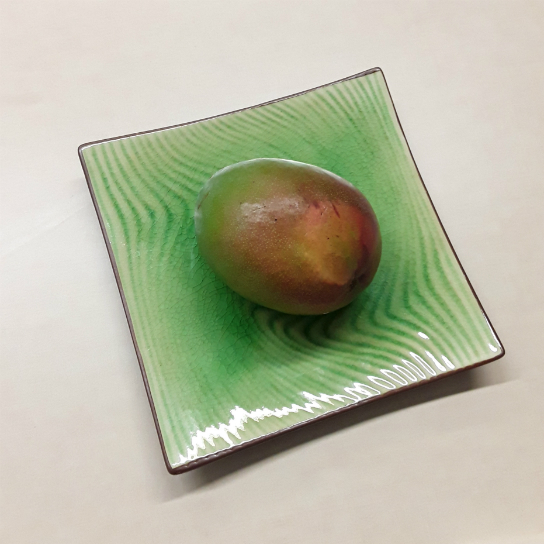

Supplement: Multimedia Appendix 2 [file formative_v4i12e15602_app2.zip › R Mango container.jpg]

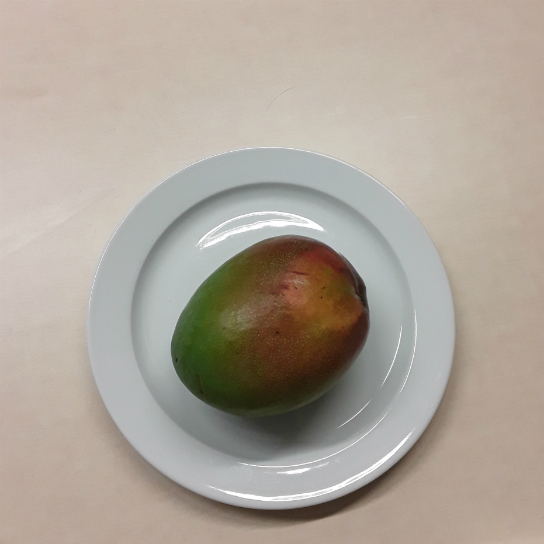

Supplement: Multimedia Appendix 2 [file formative_v4i12e15602_app2.zip › R Mango ideal.jpg]

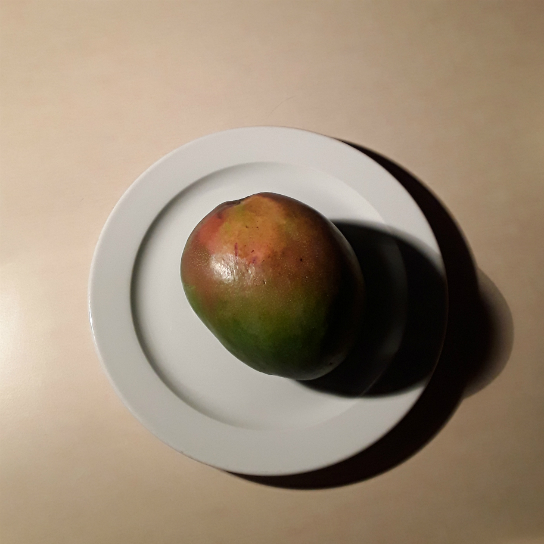

Supplement: Multimedia Appendix 2 [file formative_v4i12e15602_app2.zip › R Mango light.jpg]

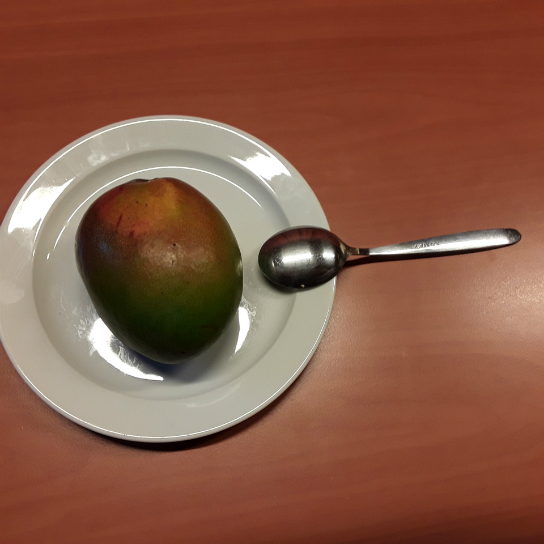

Supplement: Multimedia Appendix 2 [file formative_v4i12e15602_app2.zip › R Mango real life.jpg]

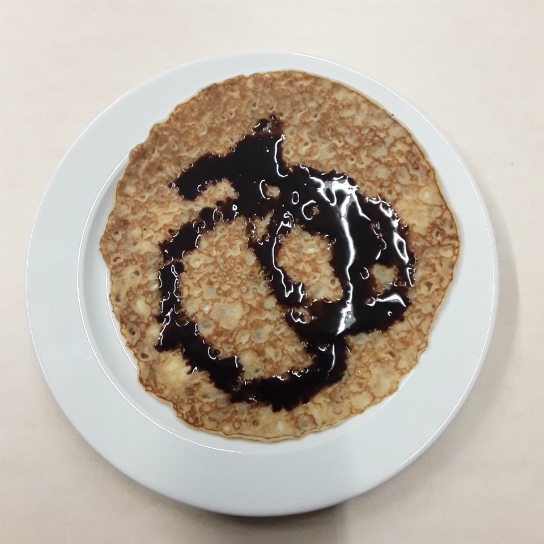

Supplement: Multimedia Appendix 2 [file formative_v4i12e15602_app2.zip › R Pancakes angle.jpg]

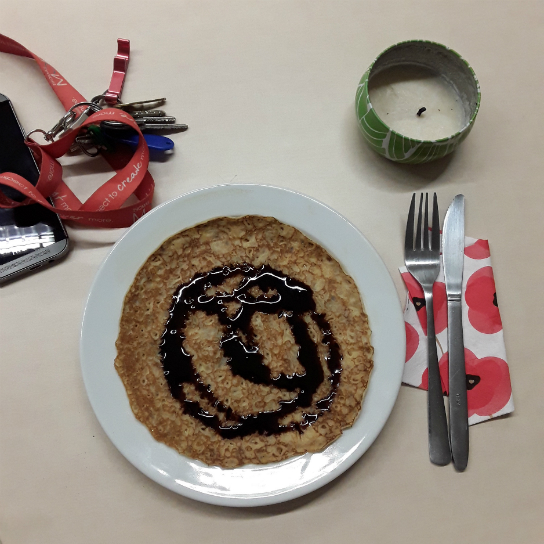

Supplement: Multimedia Appendix 2 [file formative_v4i12e15602_app2.zip › R Pancakes clutter.jpg]

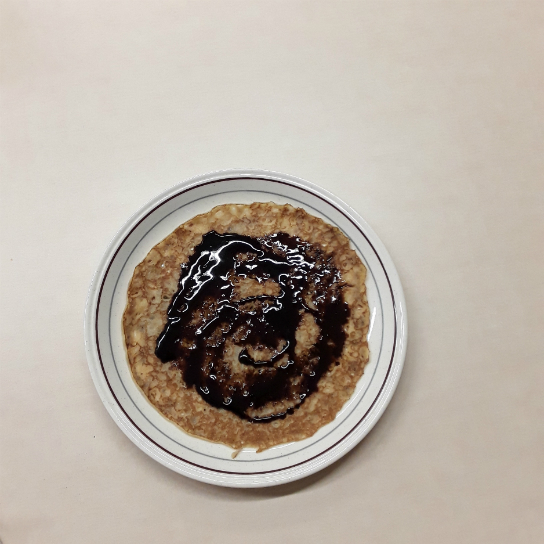

Supplement: Multimedia Appendix 2 [file formative_v4i12e15602_app2.zip › R Pancakes container.jpg]

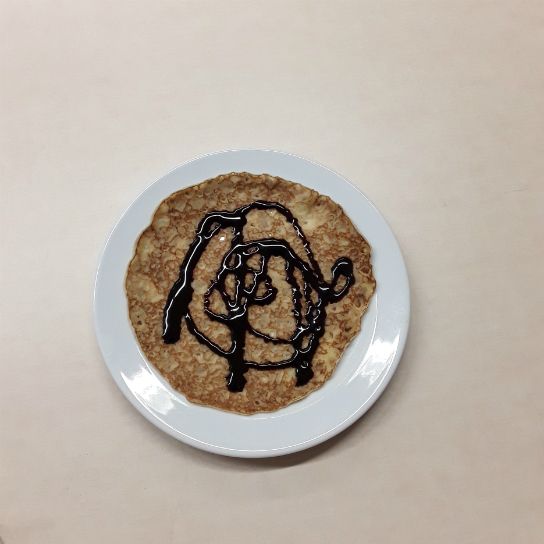

Supplement: Multimedia Appendix 2 [file formative_v4i12e15602_app2.zip › R Pancakes ideal.jpg]

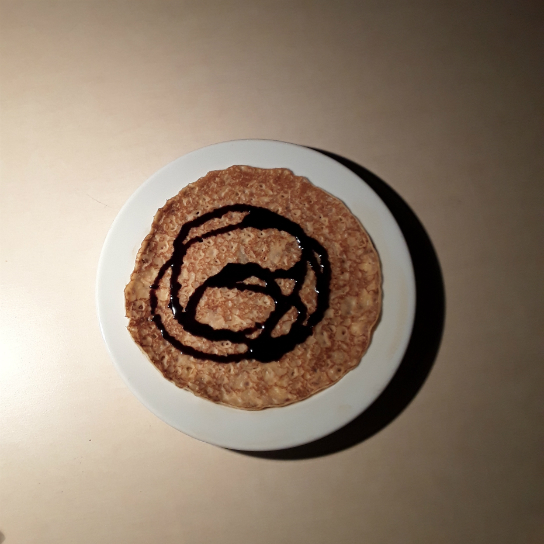

Supplement: Multimedia Appendix 2 [file formative_v4i12e15602_app2.zip › R Pancakes light.jpg]
