# Supplementary material for: Use of Different Food Image Recognition Platforms in Dietary Assessment: Comparison Study
Source: JMIR Form Res. 2020 Dec 7;4(12):e15602. doi: 10.2196/15602 (PMC7752530; doi:10.2196/15602)
Supplement: Multimedia Appendix 3 [file formative_v4i12e15602_app3.docx]

**Multimedia Appendix 3.** Mixed dishes composition.

| **Dish** | **Components** | **Dish** | **Components** |
| --- | --- | --- | --- |
| Beef stew with mashed potatoes | Stew or beef (gravy was assessed as being correct but would only account for ¼ in the totality score (equivalently ¼ gravy + ¼ beef) | Pancakes with syrup | Pancakes |
|  | Potatoes or mash |  | Syrup or molasses |
| Pork ham, beans and boiled potatoes | Ham or pork | Ravioli, could be estimated as such or as its different components | Pasta |
|  | Green beans |  | (Tomato) sauce |
|  | Potatoes | Linguini with cream sauce, mushrooms and ham | Pasta |
|  | Sauce |  | Mushrooms |
| Chicken tikka masala with rice | (Chicken) tikka masala or curry or stew |  | (Cream) sauce |
|  | Rice |  | Ham or pork |
| Caesar salad, could be estimated as such or as different separate components | Chicken | Pizza Quattro Formaggi | Pizza |
|  | Lettuce or salad | Breakfast cereal with milk | Cereal or cornflakes or muesli or granola |
|  | Croutons or bread |  | Milk |
|  | Cheese | Sandwich with chocolate spread | Bread or sandwich |
|  | Dressing or sauce |  | Chocolate or chocolate spread or Nutella |
| Tomato soup | Soup | Quiche Lorraine | Quiche |
|  | (estimate of tomatoes would count as ¼ in totality score, since it is not wrong but also is not a good estimate of what is shown in the picture) |  | |
